# In silico characterization, structural modeling, and molecular docking of *GabP* in citrus and its potential role in GABA uptake

**DOI:** 10.1038/s41598-025-07447-y

**Published:** 2025-07-04

**Authors:** Yasser Nehela, Nabil Killiny

**Affiliations:** 1 Department of Agricultural Botany, Faculty of Agriculture, Tanta University, Tanta, Egypt; 2 Department of Plant Pathology, Citrus Research and Education Center, University of Florida, 700 Experiment Station Rd., Lake Alfred, FL 33850 USA

**Keywords:** Citrus greening, TCA cycle, GABA shunt, *Gab* genes, GABA permease (*gabP*), In silico analysis, Physiology, Plant sciences

## Abstract

GABA shunt is an important metabolic hub that plays a key role in plant growth, development, and response. Although a functional connection between GABA shunt and the TCA cycle via a mitochondrial GABA permease (*gabP*) gene was reported previously from model plants, this connection in non-model plants, such as citrus, is poorly studied. Herein, we used bioinformatics and multi-omics for in-silico characterization, structural modeling, and ligand-receptor binding profile of GABA transport carrier (*CsgabP*) from the non-model plant, Valencia sweet orange (*Citrus sinensis*) and to explore their potential role(s) in GABA uptake and citrus response to Huanglongbing. Genome-wide analysis showed that the citrus genome encodes for two putative *CsgabP* proteins (*CsgabP*-1 [521 aa] and *CsgabP*-2 [419 aa]) that were relatively highly homologous to each other and homologous to amino acid permease BAT1 proteins from other plant species. Primary structures of both *CsgabPs* showed high similarity and conserved sequences between them. *CsgabPs* are highly hydrophobic integral transmembrane transporter proteins with internal hydrophilic N- and C-terminal ends and depend on 9–12 transmembrane segments. The crystallographic three-dimensional (3D) structures of both *CsgabP*s were predicted and confirmed using AlphaFold as a monomer with 100% coverage, but not experimentally determined crystallographic structures. Targeted metabolomics showed that *CsgabP* was upregulated upon GABA supplementation in healthy and ‘*Candidatus* Liberibacter asiaticus’-infected citrus plants. Likewise, *CsgabP* was involved in citrus response(s) to different biotic stress including infection with ‘*Ca*. L. asiaticus’ or infestation with its vector, *Diaphorina citri*. These findings probably clarify the molecular and functional connection between the GABA shunt and the TCA cycle in non-model plants such as citrus.

**Supplementary Information:**

The online version contains supplementary material available at 10.1038/s41598-025-07447-y.

## Introduction

Plants, unlike vertebrates, lack the somatic adaptive immune system, however, they rely on a two-branched innate immune system^1–3^. Pattern-triggered immunity (PTI) and effector-triggered immunity (ETI) are the two main components of this immune system that act successively to battle pathogen attacks^1–4^. PTI recognizes many microbes via responding to common molecules known as pathogen-associated molecular patterns (PAMPs)^1^ leading to a signaling cascade involving mitogen-activated protein kinases (MAPKs), the production of reactive oxygen species (ROS), and callose deposition^1–4^. On the other hand, ETI is a more robust response that recognizes specific pathogen virulence factors/effectors either directly or indirectly^3,5–7^. ETI triggers the hypersensitive reaction (HR), induces the biosynthesis of defense-related metabolites, and activates several defense-related genes, leading to localized cell death and/or systemic acquired resistance (SAR)^1,3^.

It is worth mentioning that both primary and secondary metabolites play an immunomodulatory role in the cross-talk between pathogenic microbes and the immune system^8–10^. For instance, the ubiquitous nonproteinogenic amino acid, *γ*-aminobutyric acid (GABA), was reported to play a key role in plant immune responses against phytopathogens^11,12^ via modulation of ROS homeostasis^11^. Additionally, GABA modulates plant growth, development, and senescence^13–15^ by directly regulating the activity of plant-specific anion transporters^13,14^. Moreover, the GABA shunt is vital for regular carbon metabolism and is functionally linked to the tricarboxylic acid (TCA) cycle^16^. It is also correlated with other stress-associated metabolites such as phytohormones and polyamines^17–20^. However, the precise nature of the connection between GABA and other metabolic pathways, as well as plant immunity-associated signaling events is poorly understood.

Interestingly, GABA is abundant in the phloem sap of several plants such as *Brassica napus*^21^*Medicago truncatula*^22,23^and *Citrus sinensis*^24^. Moreover, it is abundant in the leaf tissues of tomato (*Solanum lycopersicum*) plants^25,26^bean (*Phaseolus vulgaris*) plants^27^and sweet orange (*C. sinensis*) plants^18,19,28^. Furthermore, GABA was accumulated to higher levels in response to abiotic and biotic stress in general^29^ and fungal^25^ and bacterial infection particularly^18,19,26–28^. For example, infection with the bacterial pathogen *Pseudomonas syringae* Pv. *phaseolicola* or *P. syringae* pv. *tomato* resulted in the accumulation of higher levels of GABA with the apoplast of infected bean and tomato plants, respectively^26,27^. Likewise, GABA levels were increased upon the infection with ‘*Candidatus* Liberibacter Asiaticus’, the causal agent of citrus greening disease (aka Huanglongbing [HLB])^18,19,28^.

GABA is synthesized in the cytosol from glutamate^30–32^but broken down inside mitochondria into succinic semialdehyde (SSA) which rapidly turns into succinate^33^. Several genes enable the non-cyclic flux toward succinate via GABA shunt^34^. Previously, we proved that *gab* genes facilitate shifting the cyclic flux of the TCA cycle to an alternative non-cyclic pathway via GABA shunt and contribute to the citrus response to the bacterial pathogen ‘*Candidatus* Liberibacter asiaticus’, the causal agent of Huanglongbing^18^. It is worth mentioning that the citrus genome possesses all three putative *gab* genes including amino-acid permease (also known as GABA permease; *CsgabP*), GABA transaminase (*CsgabT*), and succinate-semialdehyde dehydrogenase (also known as GABA dehydrogenase; *CsgabD*)^34^. *CsgabP* had relatively high homology with the mitochondrial GABA permease from *A. thaliana* (*AtGABP*)^16^ and upregulated in ‘*Ca*. L. asiaticus’-infected plants^18^. However, important gaps of knowledge remain to be filled in this sense, such as the characterization and structural modeling of *CsgabP* and its role in GABA uptake in citrus.

In the current study, we comprehensively used in silico genome-wide analysis and bioinformatics to identify *gabP* homologies of the economically important non-model citrus plants. Moreover, active domains and important sites of these proteins were predicted and functionally analyzed. Likewise, three-dimensional structures, as well as, the ligand-receptor binding profile of *CsgabP* protein(s) were investigated. Furthermore, multi-omics techniques including metabolomics and transcriptomics were used to better understand the effect of GABA accumulation, whether via exogenous GABA supplementation or due to biotic stress (infection with ‘*Ca*. L. asiaticus’ and infestation with *Diaphorina citri*, the vector of the bacterial pathogen), on the expression patterns of the predicted *gabP* gene(s). The importance of this study is not only to characterize *CsgabP* and decipher its role in GABA uptake in HLB-affected trees, but probably clarify the molecular and functional connection between the GABA shunt and the TCA cycle.

## Results

### The citrus genome encodes for two putative *CsgabP* proteins

Based on the available data on the GenBank database and using the BLASTp tool, our findings showed that the *C. sinensis* genome possesses about eight AA sequences (Table S2) that were highly similar and produced significant alignment statistics with the bidirectional amino acid transporter 1 (BAT1; GenBank accession no. NP_565254.1; 516 aa) from *Arabidopsis thaliana* (aka GABA permease [*AtgabP* ]). However, only three of them (Table S3) had an identity percentage of more than 50%, including amino-acid permease BAT1-like isoform X1 (GenBank accession no. XP_006468761.1, 521 aa, identity = 80.95%; henceforth *CsgabP*-1), amino-acid permease BAT1-like isoform X2 (GenBank accession no. XP_006468762.1, 419 aa, identity = 81.75%; henceforth *CsgabP*-2), and amino-acid permease BAT1 (GenBank accession no. XP_006469954.1, 482 aa, identity = 80.71%; *CsgabP*-3). However, the record of *CsgabP*-3 (XP_006469954.1) was removed as a result of standard genome annotation processing. Therefore, we focused on only two proteins (*CsgabP*-1 and *CsgabP*-2) for further in silico analysis (Tables S2 and S3).

### Predicted *CsgabP* proteins are relatively highly homologous to each other

**Fig. 1:**
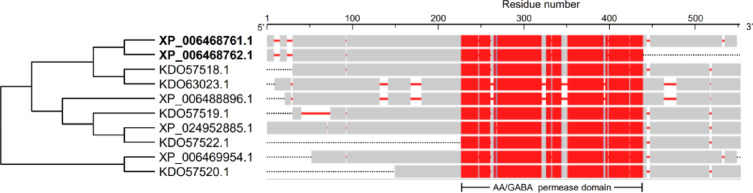
In silico analysis of putative GABA permease (*CsgabP*, aka amino-acid permease [BAT1]) from ***Citrus sinensis***. Evolutionary analysis using the maximum likelihood method and its associated multiple protein sequence alignments using Constraint-Based Alignment tool (COBALT) analysis. The listed genes were identified using the protein-protein BLAST (BLASTp) using bidirectional amino acid transporter 1 (BAT1; GenBank accession no. NP_565254.1; 516 aa) from *Arabidopsis thaliana* (aka GABA permease [*AtgabP*])^16^ as a query sequence against *Citrus sinensis* genome available in GenBank, National Center for Biotechnology Information website (NCBI, http://www.ncbi.nlm.nih.gov/gene/), using the compositionally adjusted substitution matrices^35^. Evolutionary analyses and the joint tree were conducted in MEGA-X software^36^. In the COBALT analysis, residues were colored using a column-based method according to their relative entropy threshold. Aligned columns with no gaps are colored blue and red, where the red color indicates highly conserved columns and blue indicates less conserved ones. The full list of genes, names, and accession numbers is available in supplementary Tables S2 and S3.

Although the multiple protein sequences alignment using COBALT analysis showed that the obtained eight AA sequences were relatively highly homologous to each other (Fig. 1), its associated phylogenetic analysis showed that only two proteins were phylogenetically closer (about 77%) including *CsgabP*-1 and *CsgabP*-2 (Fig. 1). Interestingly, both *CsgabPs* proteins were matched with the bidirectional amino acid transporter 1 (orange1.1g011598m; 482 aa) from the “*Citrus sinensis proteins*, phytozome 154 v1.1” BLAST dataset available on Citrus Greening Solutions website (Table S3). On the other hand, the obsolete record of *CsgabP*-3 was clustered separately with the hypothetical protein CISIN_1g010352mg (GenBank accession no. KDO57520.1; 394 aa).

### Physicochemical properties of predicted *CsgabP* proteins

The physicochemical properties of putative *CsgabP* proteins from *C. sinensis* compared with *AtgabP* from *A. thaliana* as obtained using the ExPASy ProtParam tool are summarized in Table S4. It is worth mentioning that most of, if not all, calculated physicochemical properties of putative *CsgabP* proteins from *C. sinensis* were relatively comparable to *AtgabP* from *A. thaliana*. For instance, the theoretical isoelectric point (pI) ranged from 6.37 (*CsgabP*-2) to 8.17 (*AtgabP*) which was very similar to *CsgabP*-1 (8.16) (Table S4). Furthermore, *CsgabP*-1 (521 aa) had the highest molecular weight (MW; 56.07 KDa) followed by *AtgabP* (516 aa; 55.33 KDa) whereas *CsgabP*-2 (419 aa) had the lowest MW (45.13 KDa). Extinction coefficients (ε) ranged from 77,600 M^−1^ cm^−1^ (*CsgabP*-2) to 110,030 M^−1^ cm^−1^ (*AtgabP*) at 280 nm assuming all pairs of Cys residues form cystines, however, it ranged from 77,350 to M^−1^ cm^−1^ for both proteins, respectively assuming all Cys residues are reduced (Table S4). Additionally, both predicted *CsgabP* proteins (*CsgabP*-1 and *CsgabP*-2) were classified as stable proteins since both had instability index (II) below 40 (26.27 and 30.98, respectively). Likewise, relative similarities were observed between putative *CsgabP* proteins from *C. sinensis* and *AtgabP* from *A. thaliana* in terms of other physiochemical features including aliphatic index, the total number of negatively charged residues (Asp + Glu), the total number of positively charged residues (Arg + Lys), and hydropathicity (GRAVY) (Table S4).

### Predicted *CsgabP*s are homologous to BAT1 proteins from other plant species

**Fig. 2:**
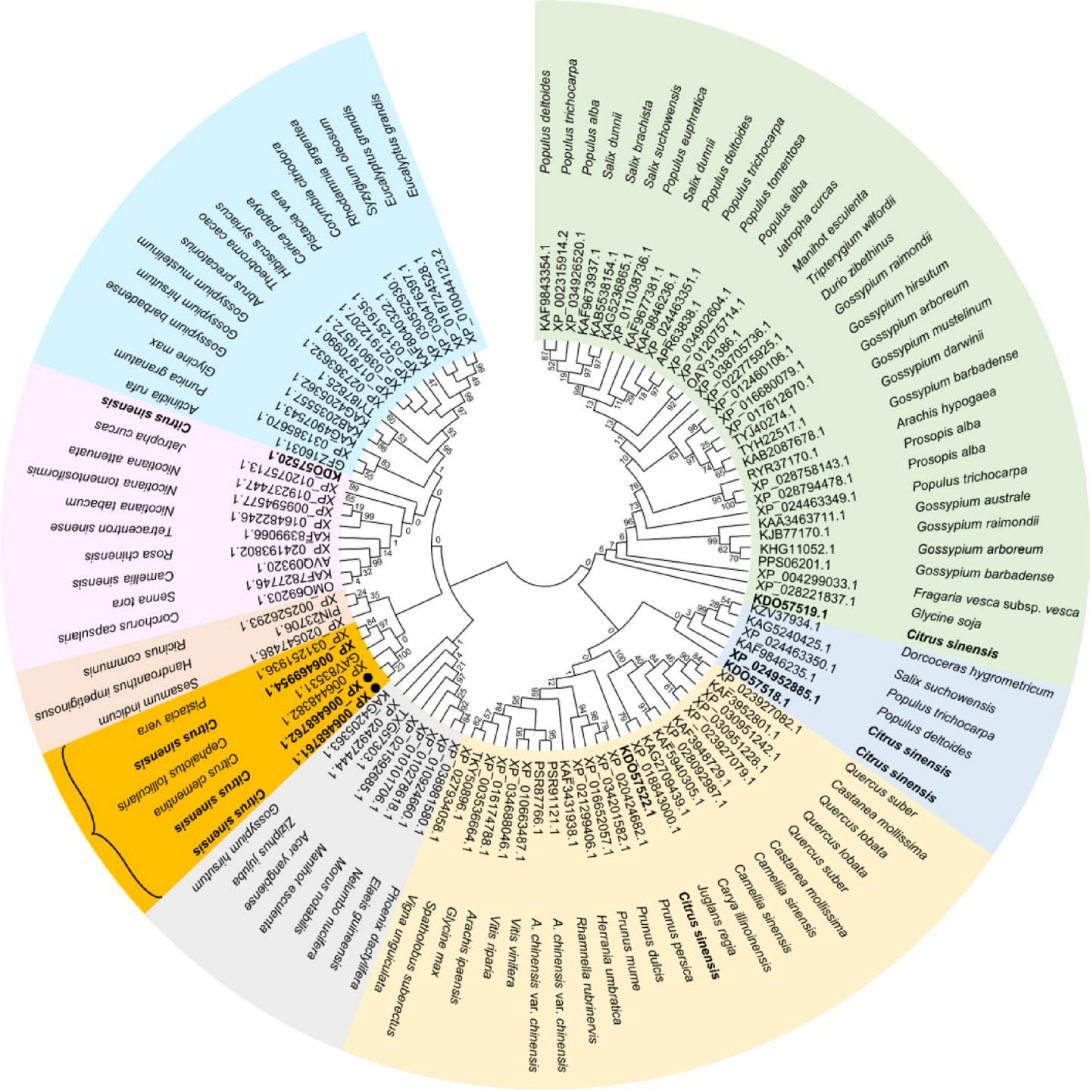
Evolutionary relationships of GABA permease (*gabP*, aka amino-acid permease [BAT1]) proteins from different plant species. The evolutionary history was inferred by using the Maximum Likelihood method and JTT matrix-based model^37^. The tree with the highest log likelihood (-65227.88) is shown. The percentage of replicate trees in which the associated taxa clustered together in the bootstrap test (500 replicates) are shown next to the branches^38^. Initial tree(s) for the heuristic search were obtained automatically by applying Neighbor-Join and BioNJ algorithms to a matrix of pairwise distances estimated using the JTT model and then selecting the topology with a superior log-likelihood value. This analysis involved 106 amino acid sequences. There were a total of 1052 positions in the final dataset. Evolutionary analyses were conducted in MEGA X^36^. Putative GABA permeases of interest from *Citrus sinensis* (*CsgabP*) are bolded and marked by a black circle. The listed genes were assembled based on recently available data in GenBank, National Center for Biotechnology Information website (NCBI, https://www.ncbi.nlm.nih.gov/protein/). The full list of genes, names, and accession numbers is available in supplementary Table S5.

The homology of *CsgabPs* from *C. sinensis* to *BAT1* proteins from other plant species was significantly high (more than 80% identity; Table S5) and it showed high similarity and conserved amino acid permease/GABA permease domain when aligned with these sequences from other plant species (Figs. 2 and S1). However, the phylogenetic analysis revealed that the AA sequence of *CsgabP*-1 and *CsgabP*-2 were phylogenetically closer to AA permease BAT1 from *Citrus clementina* (GenBank accession no. XP_006448382.1) and AA_permease_2 domain-containing protein from *Cephalotus follicularis* (GenBank accession no. GAV83531.1) than the obsolete record of *CsgabP*-3 which was phylogenetically closer to AA permease BAT1 homolog isoform X2 from *Pistacia vera* (GenBank accession no. XP_031251936.1) with high bootstrap values (Figs. 2 and S1).

### *CsgabPs* proteins are similar and have conserved sequences between them

**Fig. 3:**
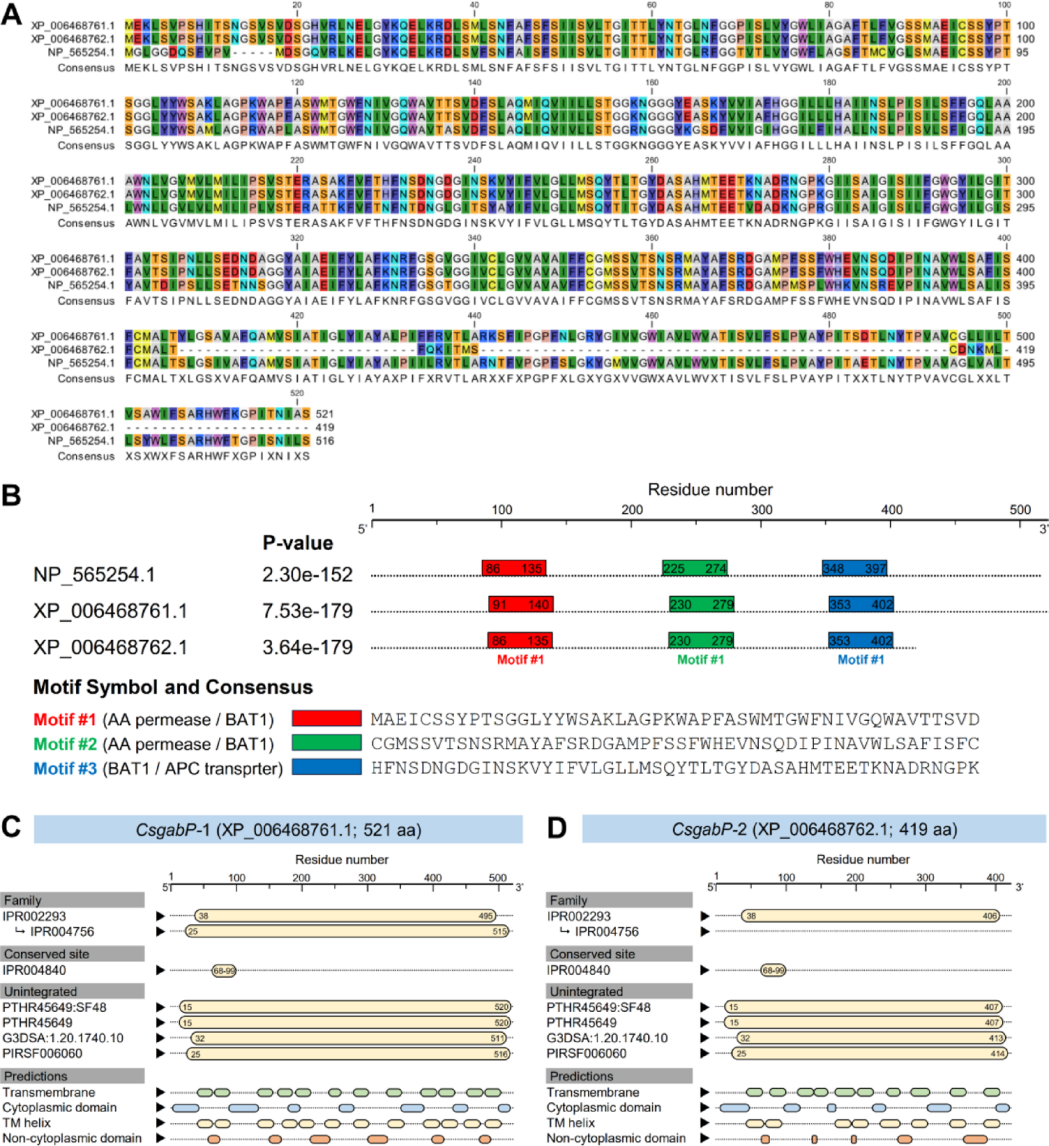
In silico analysis of putative GABA permeases (*CsgabP*, aka amino-acid permease [BAT1]) from ***Citrus sinensis***. (**A**) Multiple sequence alignment of the top matched AA sequence of putative *CsgabPs* from *C. sinensis* along with the query sequence of bidirectional amino acid transporter 1 (BAT1; GenBank accession no. NP_565254.1; 516 aa) from *Arabidopsis thaliana* (aka GABA permease [*AtgabP*])^16^. Clustal Omega algorithm^39^ and QIAGEN CLC Genomics Workbench 24.0.2 were used for the multiple sequence alignment and visualization of retrieved AA sequences. Conserved amino acids and similar residues are shaded using RasMol coloring. (**B**) Novel motifs of *CsgabP-*1 (XP_006468761.1) and *CsgabP-*2 (XP_006468762.1) from sweet orange (*C. sinensis*), along with the query protein sequence, *AtgabP* (NP_565254.1). Motifs were discovered using Multiple Em for Motif Elicitation (MEME) Suite-version 5.5.7 (http://meme.sdsc.edu/meme/meme.html, accessed on October, 2nd, 2024)^40^. (**C** and **D**) Functional and conserved domains analysis of *CsgabP-*1 (XP_006468761.1) and *CsgabP-*2 (XP_006468762.1), respectively, using the InterPro Scan tool. Family and conserved sites corresponding to the entry of three contributing databases (InterPro, PANTHER, and PIRSF) as follows; IPR002293: amino acid/polyamine transporter I family, IPR004756: amino acid permease subfamily, IPR004840: amino acid permease conserved site, PTHR45649:SF48: amino-acid permease BAT1 homolog, PTHR45649: amino-acid permease BAT1, G3DSA:1.20.1740.10: amino acid/polyamine transporter I, and PIRSF006060: amino acid transporter. The full list of genes, names, and accession numbers is available in supplementary Table S2.

The multiple sequence alignment of the top matched *CsgabPs* proteins (*CsgabP*-1 and *CsgabP*-2) from *C. sinensis* showed high similarity and conserved sequences between them, as well as with the query sequence of *AtgabP* from *A. thaliana* (Fig. 3A). Both *CsgabP* proteins were almost identical except that *CsgabP*-2 was 102-aa shorter than *CsgabP*-1. Interestingly, three tandem motifs were discovered within the protein sequences of both *CsgabP*s from sweet orange (*C. sinensis*) and the query sequence of *AtgabP* from *A. thaliana* using MEME Suite (Fig. 3B). A set of 50 AA residues represents each motif. Motif #1 (MAEICSSYPTSGGLYYWSAKLAGPKWAPFASWMTGWFNIVGQWAVTTSVD) was consistently discovered within all studied sequences revealing its identity with the amino acid permease (Pfam entry: PF13520) and the amino acid/polyamine transporter I family (InterPro entry: IPR002293). Likewise, motif #2 (CGMSSVTSNSRMAYAFSRDGAMPFSSFWHEVNSQDIPINAVWLSAFISFC) was conserved among all studied *gabP* protein sequences and was also associated with transmembrane transporter activity (Fig. 3B). Finally, motif #3 (HFNSDNGDGINSKVYIFVLGLLMSQYTLTGYDASAHMTEETKNADRNGPK) was identical to the amino-acid permease BAT1 (PANTHER entry: PTHR45649), amino acid/polyamine transporter I (InterPro entry: IPR002293), and a TMHMM: TM-helix which is a region of a membrane-bound protein predicted to be embedded in the membrane.

Moreover, classifying protein families using the InterPro Scan tool provided similar functional analysis and interactively predicted the conserved domains. The InterPro analysis suggested a high topological similarity between *CsgabP*-1 (Fig. 3C) and *CsgabP*-2 (Fig. 3D). Briefly, both sequences had an amino acid/polyamine transporter I family (InterPro entry: IPR002293). However, *CsgabP*-1 had an amino acid permease subfamily (InterPro entry: IPR004756) that was absent in *CsgabP*-2 (Fig. 3D). Moreover, both sequences had an amino acid permease conserved site (InterPro entry: IPR004840) with four unintegrated sites including amino-acid permease BAT1 homolog (PANTHER entry: PTHR45649:SF48), amino-acid permease BAT1 (PANTHER entry: PTHR45649), amino acid/polyamine transporter I (CATH-Gene3D entry: G3DSA:1.20.1740.10), and amino acid transporter (PIRSF entry: PIRSF006060) (Fig. 3C and D). Additionally, like other transporters, *CsgabPs* were predicted to have several transmembrane regions (12 and 10 regions), cytoplasmic domains (7 and 6 domains) transmembrane helices (12 and 8 helices), and some non-cytoplasmic domains (6 and 5 domains) for *CsgabP*-1 (Fig. 3C) and *CsgabP*-2 (Fig. 3D), respectively. Collectively, these findings suggest that both predicted *CsgabP* proteins were highly similar in their functional analysis, conserved domains, and topology.

### Secondary structure analysis of predicted *CsgabP* proteins

NPS@-based secondary structure analysis revealed that the three *gabP* proteins (*AtgabP*, *CsgabP-*1, and *CsgabP-*2) are dominated by over 48% of amino acids residing in α-helices (Table S6). On the other hand, extended strands represented 17.44, 17.27, and 18.38% for *AtgabP*, *CsgabP-*1, and *CsgabP-*2, respectively. It is worth noting that random coils represented a considerable percentage of the secondary structure of *AtgabP* (31.78%), *CsgabP-*1 (32.05%), and *CsgabP-*2 (29.36%). Nevertheless, just a few AA residues were involved in the beta turns of *AtgabP* (2.13%), *CsgabP-*1 (1.73%), and *CsgabP-*2 (3.34%). Interestingly, none of the studied *gabP* proteins had 3_10_-helices, π-helices, beta bridges, bend regions, ambiguous states, or any other states (Table S6).

The secondary structures of putative *CsgabP*s were further and deeply investigated using the PDBsum tool (Figure S2). Briefly, the secondary structure-associated ProMotif of *CsgabP*-1 (Figure S2A) presented 27 helices (H1 – H27; Table S7) involved in about 55 helix-helix interactions between them (Table S8) with 39 beta turns (Table S9) and 9 gamma turns (Table S10). Helices of *CsgabP*-1 were mostly alpha helix type combined with only six 3_10_ helices (H2, H10, H14, H15, H17, and H23) (Table S7). Moreover, the 39 predicted beta turns were assigned to five classes based on the phi, psi angles of residues i + 1 and i + 2 with the majority of type IV (20 *β*-turns), followed by 14 *β*-turns type I, two of each type I’ and type VIII, and only one *β*-turns type II (Table S9). Besides, out of the nine discovered gamma turns, two-thirds of them were ‘Inverse’ type and only three were ‘Classic’ gamma turns (Table S10).

Likewise, the secondary structure-associated ProMotif of *CsgabP*-2 (Figure S2B) displayed 22 helices (H1 – H22; Table S11) involved in only 38 helix-helix interactions between them (Table S12) with 30 beta turns (Table S13) and six gamma turns (Table S14). Most of the 22 helices of *CsgabP*-2 were *α*-helix with only five 3_10_ helices (H2, H13, H14, H16, and H21) (Table S11). Furthermore, the 30 predicted *β*-turns were assigned to only four classes based on the phi, psi angles of residues i + 1 and i + 2 with most of the type I and IV (13 *β*-turns of each type) and two of each type I’ and II (Table S13). Although only six γ-turns were discovered within the secondary structure of *CsgabP*-1, only one ‘Classic’ turn was reported, whereas the rest were ‘Inverse’ type (Table S14).

Moreover, the topology diagrams of *CsgabP*-1 (Figure S2C) and *CsgabP*-2 (Figure S2D) were generated to visualize better the arrangement and connectivity of the helices and strands in both proteins. Although the topology plot of *CsgabP*-2 was five helices shorter than *CsgabP*-1, their topology remains almost the same. This implies that both *CsgabP*-1 and *CsgabP*-2 are closely related and that similar folds may have been similarly evolutionally acquired.

### The crystallographic 3D structures of *CsgabP* proteins

The crystallographic three-dimensional (3D) structures of both *CsgabPs* (*CsgabP*-1 and *CsgabP*-2) were predicted using SWISS-MODEL and AlphaFold but not experimentally determined crystallographic structures. Initially, 3D structures of both *CsgabPs* were predicted using SWISS-MODEL using the crystal structure of probable glutamate/gamma-aminobutyrate antiporter (aka glutamate-GABA antiporter [GadC] from *Escherichia coli* (strain K12) in the protein data bank (PDB ID: 4dji.1.A) and refined using X-ray method to 3.19 Å resolution with acceptable statistics (Figure S3). Briefly, approximately 87% AA sequence (residues Lys 35 to Ala 508) of *CsgabP*-1 has been modeled with the template protein (sequence identity = 14.98%, sequence similarity = 27%, and confidence = 100%) with accepted Global Model Quality Estimation (GMQE = 0.47) and good absolute quality (QMEAN Z-score = -9.05) (Figure S3A). The predicted 3D structure of *CsgabP*-1 is a monomer composed of 17 *α*-helix ribbons (four of them are short, less than 10 residues) and four *β*-sheets (Figures S3A and S3B) with considerable predicted local similarity (PLS) to target (Figure S3C). Likewise, approximately 83% (residues Lys 35 to Leu 405) of *CsgabP-*2 have been modeled with the target protein (sequence identity = 14.37%, sequence similarity = 27%, and confidence = 100%) with satisfactory GMQE and QMEAN (0.43 and − 7.67, respectively) (Figure S3D). The predicted model of *CsgabP-*2 is a monomer that contains 15 *α*-helices and only one stranded *β*-sheet (Figures S3D and S3E) with significant PLS to the target (Figure S3F).

**Fig. 4:**
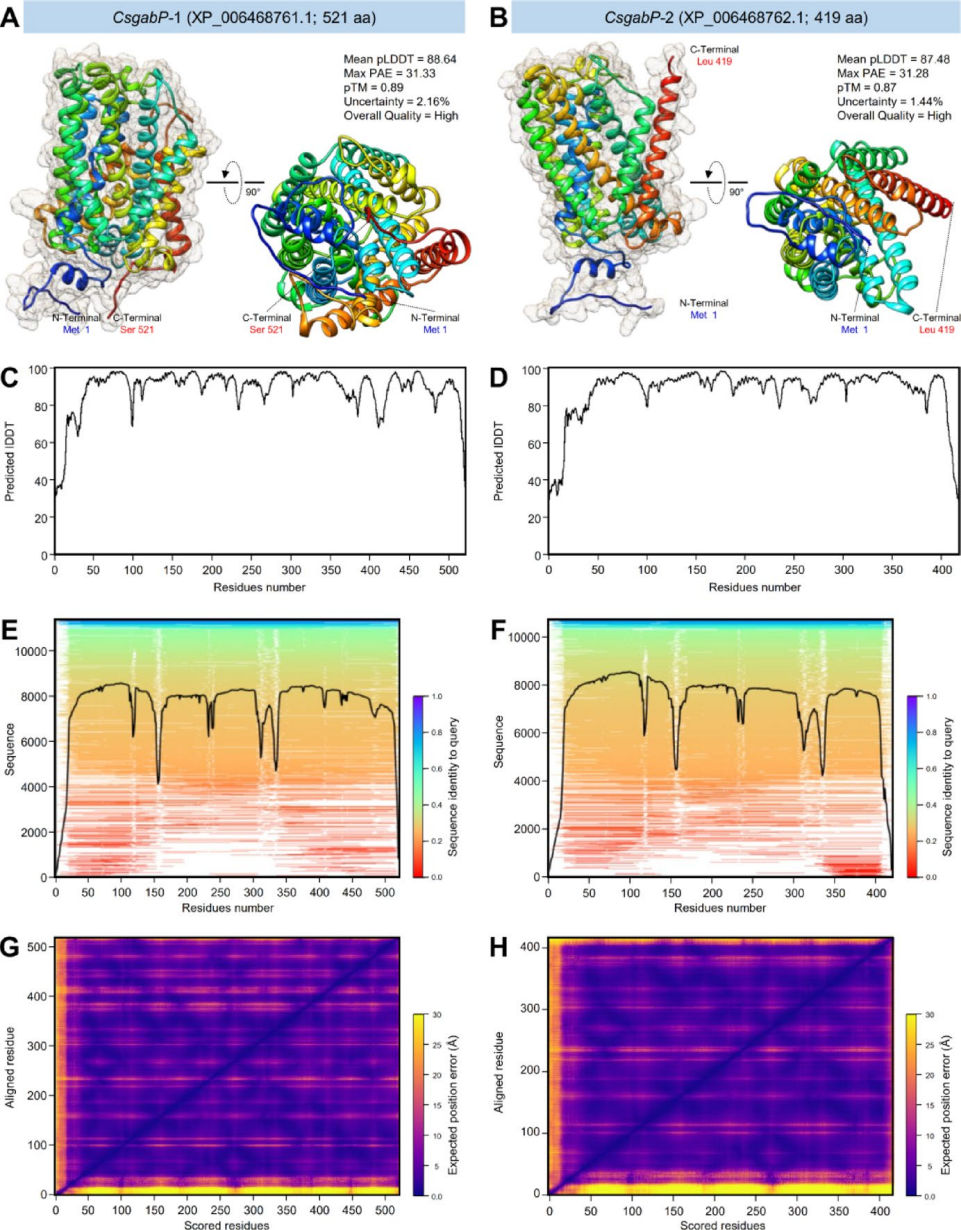
The crystallographic tertiary structure of putative GABA permeases (*CsgabP*, aka amino-acid permease [BAT1]) from *Citrus sinensis* using AlphaFold. (**A** and** B**) AlphaFold2-based predicted three-dimensional (3D) structure model and its associated mesh surface of *CsgabP-*1 (XP_006468761.1) and *CsgabP-*2 (XP_006468762.1), respectively. The tertiary structures of *CsgabPs* were predicted using the AlphaFold2^41,42^ model based on ColabFold^43^ using the multiple sequence alignment (MSA) mode of “mmseqs2_uniref_env” of five cycles and recycle early stop tolerance of 0.75. The tertiary structures were generated using the Neurosnap platform for computational biology tools (https://neurosnap.ai/service/AlphaFold2, accessed on October, 9th, 2024) and visualized with the UCSF-Chimera package. Protein chains are colored according to the rainbow color spectrum, from blue (N-terminus) to red (C-terminus). (**C** and** D**) Predicted local distance difference test (pLDDT), (**E** and** F**) Multiple sequence alignment (MSA) sequence Coverage, and (**G** and** H**) Predicted aligned error.

Moreover, AlphaFold2 was used to regularly predict the 3D structures of the full-length AA sequences of both *CsgabP* proteins with atomic accuracy without a specific template. Briefly, 100% AA sequence (residues Met 1 to Ser 521) of *CsgabP*-1 (Fig. 4A), as well as 100% AA sequence (residues Met 1 to Leu 419) of *CsgabP*-2 (Fig. 4B) has been modeled with very low uncertainty (2.16 and 1.44%; for *CsgabP*-1 and *CsgabP*-2; respectively) which reflect a high overall quality of both predicted structures (Fig. 4A and B). The predicted local distance difference test (pLDDT) that allows us to infer the accuracy of each predicted residue’s spatial orientation and position showed that both predicted 3D structures were predicted with relatively high pLDDT (Mean pLDDT = 88.64 and 87.48, for *CsgabP*-1 and *CsgabP*-2; respectively) (Fig. 4C and D). Furthermore, 2D heatmaps of predicted aligned error (PAE) displayed that both models are confident in the residue-residue interactions (Max PAE = 31.33 and 31.28; for *CsgabP*-1 and *CsgabP*-2; respectively) (Fig. 4G and H). Moreover, the predicted template modeling (pTM) score for the superposition between the predicted structure and the hypothetical true structure of both *CsgabP*-1 (Fig. 4E) and *CsgabP*-2 (Fig. 4F) suggests a reasonable prediction based on the obtained high pTM (0.89 and 0.87; respectively).

### Putative *CsgabP*s are integral transmembrane transporter proteins

**Fig. 5:**
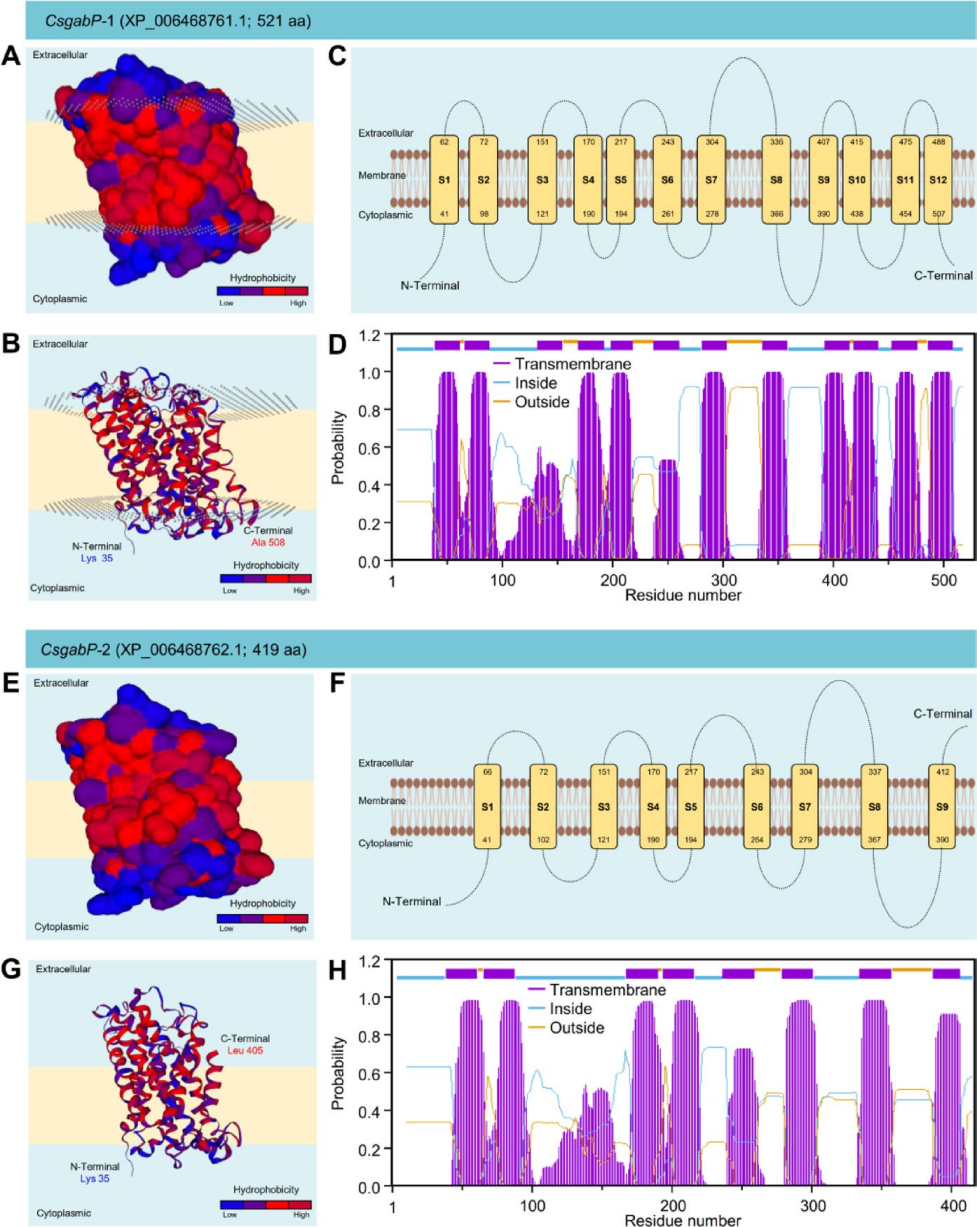
The predicted topology of putative GABA permeases (*CsgabP*, aka amino-acid permease [BAT1]) from *Citrus sinensis*. (**A** and **E**) Membrane prediction and surface topology of *CsgabP-*1 (XP_006468761.1), and *CsgabP-*2 (XP_006468762.1), respectively. (**B** and** G**) Membrane prediction and cartoon organization of *CsgabP-*1 and *CsgabP-*2, respectively. Bio-units of transmembrane proteins are identified in the SMTL solely based on structural information. The membrane annotation is transferred to a model if at least 80% of all biounit-transmembrane residues are aligned with the target sequence(s). Protein chains are colored according to their hydrophobicity. Low hydrophobic residues are colored blue, whereas most hydrophobic residues are colored red (see the scale at the right bottom corner of the graph). (**C** and **F**) Schematic representation of Phyre2-based predicted topology of *CsgabP-*1, *CsgabP-*2, and *CsgabP-*3, respectively. Numbers inside the transmembrane (TM) domains (yellow rectangle) denote AA residues. (**D** and **H**) TMHMM posterior probabilities.

Furthermore, in silico analysis showed that both *CsgabPs* are integral transmembrane transporter proteins (Fig. 5). Briefly, *CsgabP-*1 is a highly hydrophobic transmembrane protein (Fig. 5A) with internal hydrophilic N- and C-terminal ends (Fig. 5B) and depends on 12 transmembrane regions and 11 connecting loops (Fig. 7C). Transmembrane helix prediction analyzed by the TMHMM server supports these findings with considerable TMHMM posterior probabilities (up to 1) except for only two transmembrane regions (S3 and S6) (Fig. 5D). Likewise, *CsgabP-*2 is a highly hydrophobic protein (Fig. 5E) with internal hydrophilic N- and external hydrophobic C-terminal end (Fig. 5F), however, it is composed of only nine transmembrane domains and eight connecting loops (Fig. 5G). TMHMM-based transmembrane helix analysis partially supports these findings with substantial posterior probabilities (up to 1) except for only two transmembrane regions (S3 and S6) (Fig. 5H). However, TMHMM-based transmembrane helix analysis suggests the presence of only eight membrane-spanning regions flanked by internal hydrophilic N- and C-terminal domains. Collectively, these findings showed that both *CsgabP* candidates from *C. sinensis* were highly similar and structurally homologous to each other, which suggests that they might be different copies of the same gene.

### Putative *CsgabP*s interact with GABA shunt-associated enzymes and superoxide dismutases (SODs)

**Fig. 6:**
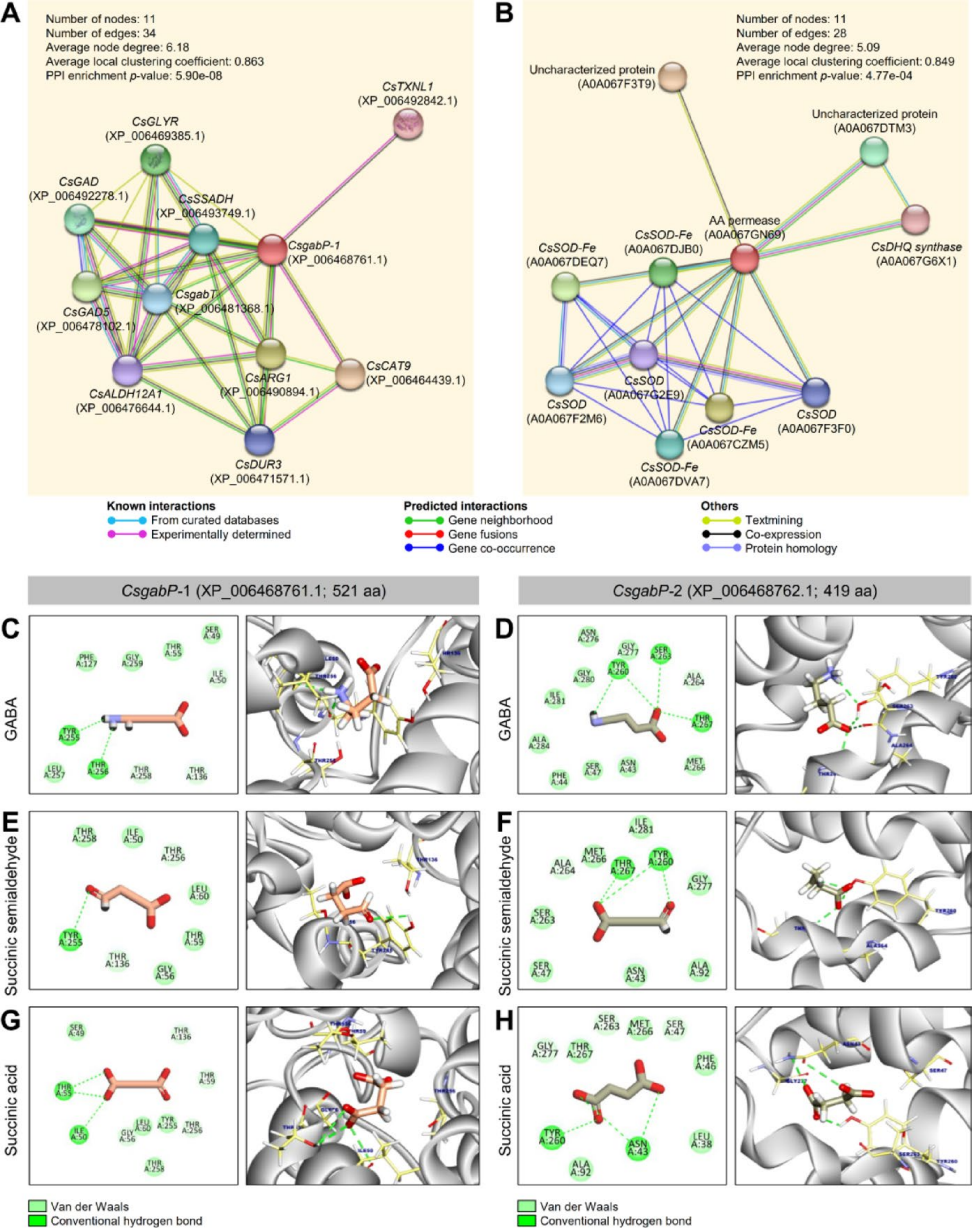
The protein-protein and docking interactions of putative GABA permeases (*CsgabP*, aka amino-acid permease [BAT1]) from *Citrus sinensis*. (**A** and **B**) Protein-protein interaction network of *CsgabP* as predicted using STRING 11.0 (https://version-11-0.string-db.org/ accessed on May, 15th, 2020) and STRING 12.0 (https://string-db.org/, accessed on October, 2nd, 2024)^44^respectively. All known and predicted interactions between proteins were integrated, including direct (physical) and indirect (functional) associations. The minimum required interaction score was preset to 0.2. The red node represented *CsgabP*, and the other nodes represented its predicted functional partners. (**C** and **D**) 2D and 3D docking interactions of GABA against *CsgabP*-1 and *CsgabP*-2, respectively. (**E** and **F**) 2D and 3D docking interactions of succinic semialdehyde against *CsgabP*-1 and *CsgabP*-2, respectively. (**G** and **H**) 2D and 3D docking interactions of succinic acid against *CsgabP*-1 and *CsgabP*-2, respectively.

Predicted functional partners of *CsgabP*-1 as predicted through protein-protein interaction networks functional enrichment analysis using STRING-11 Consortium (Fig. 6A and Table S15) showed that the prediction confidence scores ranged from 0.693 to 0.420 indicating functional network among *CsgabP*-1 and other 10 proteins from *C. sinensis.* Interestingly, *CsgabP* was predicted to interact with 10 proteins (Fig. 6A and Table S15) differently. These proteins are mainly involved in the GABA shunt, including *CsCAT9*, *CsARG1*, *CsGAD5*, *CsGLYR*, *CsGAD*, *CsSSADH*, *CsgabT*, *CsDUR3*, *CsALDH12A1*, and *CsTXNL1*. The protein-protein interaction (PPI) network of *CsgabP* comprised 11 nodes connected with 34 different edges (Average node degree = 6.18, Average local clustering coefficient = 0.863, and PPI enrichment *p*-value = 5.90e-08). Likewise, STRING-12-based PPI showed that *CsgabP* interacts with four SOD-Fe, three SOD, two uncharacterized proteins, and 3-Dehydroquinate synthase domain-containing protein with confidence scores ranged from 0.373 to 0.361 (Fig. 6B and Table S16). The PPI analysis using STRING-12 predicted 11 nodes connected with 28 different edges with an average node degree of 5.09, an average local clustering coefficient of 0.849, and the PPI enrichment *p*-value was observed to be 4.77e-04.

### Docking studies suggest that the residues involved in GABA binding are conserved between *CsgabP*-1 and ***CsgabP***-2

To better understand the binding modes of GABA and its subsequent mitochondrial metabolites, succinic semialdehyde and succinic acid with the putative *CsgabP*s, their ability to bind the active site(s) of the predicted protein-structures was computationally investigated via molecular docking analysis (Fig. 6C and H). Initially, multiple sequence alignment of AA sequences of putative *CsgabP*-1 and *CsgabP*-2 were aligned using the CLUSTALW tool (Figure S4A). Then, the 3D structural alignment for both putative *CsgabP*s was preformed (Figure S4B) to identify the conserved binding pockets (Figure S4C) and exclude false-positive variations. 3D molecular structures were analyzed using structure comparison, with the MatchMaker tool that superimposed structures based on sequence alignments and interactively visualized using UCSF Chimera.

Generally, molecular docking provides a preliminary assessment of ligand-protein interactions. Although the binding modes of the three tested compounds exhibited slight interactions with binding energies equal to − 4.26, − 4.08, and − 4.35 kcal/mol with *CsgabP*-1, respectively, and − 4.10, − 4.07, and − 4.23 kcal/mol with *CsgabP*-1, respectively (Table S17). Molecular docking analysis showed that GABA interacted by two conventional hydrogen bonds with residues Tyr 255 and Thr 256 of *CsgabP*-1 (Fig. 6C) and three hydrogen bonds with Tyr 260 (two H-bonds), Ser 263, and Thr 267 of *CsgabP*-2 (Fig. 6D). It is worth mentioning that GABA exposed acceptable root-mean-square deviations of atomic locations (RMSD = 2.72 and 2.70 Å; with *CsgabP*-1 and *CsgabP*-2, respectively) (Table S17). On the other hand, the succinic semialdehyde formed one hydrogen bond with the AA residue Tyr 255 of *CsgabP*-1 (Fig. 6E), and three conventional hydrogen bonds with the AA residues Tyr 260 (two H-bonds), and Thr 267 of *CsgabP*-2 (Fig. 6F) with accepted docking (Affinity) scores and RMSD-Refines (Table S17). Nevertheless, succinic acid formed three hydrogen bonds with *CsgabP*-1 (Thr 55 [2 H-bonds] and Ile 50; Fig. 6G) as well as three hydrogen bonds with *CsgabP*-2 (Asn 43 [2 H-bonds] and Tyr 260; Fig. 6H) with putative docking (Affinity) scores and RMSD-Refines (Table S17). Collectively, these findings suggest that the residues involved in GABA binding are conserved between *CsgabP*-1 and *CsgabP*-2, but with slight Deportation due to the variation in AA sequence length.

### *CsgabP* is positively correlated with GABA accumulation in citrus plants

#### GABA supplementation enhanced the accumulation of endogenous GABA of healthy and ‘*Ca*. L. asiaticus’-infected citrus plants

**Fig. 7:**
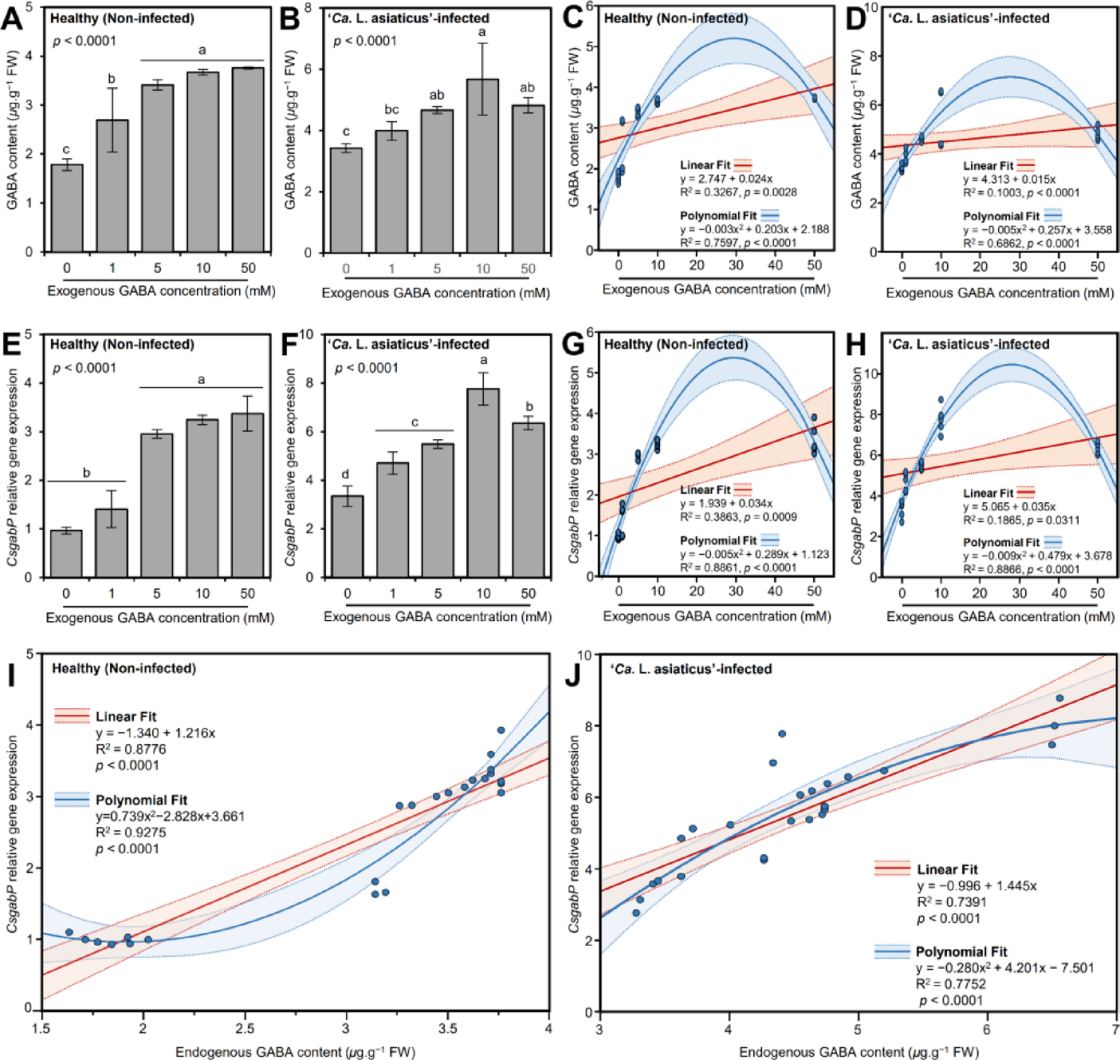
Effects of exogenous GABA application on the endogenous GABA content and its transport carrier *CsgabP* from healthy and Huanglongbing-affected Valencia sweet orange (*Citrus sinensis*). (**A** and **B**) Endogenous levels of GABA after the treatment with different GABA concentrations (0, 1, 5, 10, and 50 mM) from healthy and ‘*Ca.* L. asiaticus’-infected Valencia sweet orange, respectively. (**C** and **D**) Simple linear regression (SLR) and second-degree polynomial regression (SPR) analyses between exogenous GABA concentration (mM) and endogenous GABA content (*µ*g.g^− 1^ FW) from healthy and ‘*Ca.* L. asiaticus’-infected Valencia sweet orange, respectively. (**E** and **F**) Relative gene expression of *CsgabP* after the treatment with different GABA concentrations (0, 1, 5, 10, and 50 mM) from healthy and ‘*Ca.* L. asiaticus’-infected Valencia sweet orange, respectively. (**G** and **H**) SLR and SPR analyses between exogenous GABA concentration (mM) and *CsgabP* relative gene expression from healthy and ‘*Ca.* L. asiaticus’-infected Valencia sweet orange, respectively. (**I** and **J**) SLR and SPR analyses between endogenous GABA content (*µ*g.g^− 1^ FW) and *CsgabP* relative gene expression from healthy and ‘*Ca.* L. asiaticus’-infected Valencia sweet orange, respectively. In panels A, B, E, and F, the data presented are the means ± standard deviation (mean ± SD) of six biological replicates (*n* = 6). Different letters signify statistically significant differences among treatments, whereas the same letters indicate no significant differences among them using Tukey’s HSD (*p* < 0.05). In SLR and SPR panels, blue dots represent raw data (*n* = 6). The fitted SLR line is presented as a red line, while SPR models are presented as blue lines. The 95% confidence intervals for the estimated regression are shaded with the same color and edged by dotted lines. Regression equations for both SLR and SPR models, *R*^2^and *p*-value based on the *F* test (*p* ≤ 0.05) were also obtained and are presented within the graphs.

Generally, exogenous GABA application via root drenching significantly induced the accumulation of endogenous GABA levels of non-infected (Fig. 7A) and ‘*Ca*. L. asiaticus’-infected citrus plants (Fig. 7B) in a dose-dependent manner, till 5-10mM. It is worth mentioning that the application of 10 mM GABA significantly elevated the GABA content (5.68 + 1.18 *µ*g.g^− 1^ FW) in ‘*Ca*. L. asiaticus’-infected plants, followed by 5 mM and 50 mM GABA (4.67 + 0.11 and 4.82 + 0.26 *µ*g.g^− 1^ FW, respectively) did not differ significantly from each other (Fig. 7B). However, no significant differences were noticed between the highest three GABA doses (5, 10, or 50 mM) in healthy treated plants (Fig. 7A). These findings suggest that while 5 mM GABA was enough to maximize the endogenous GABA content within healthy citrus plants, 10 mM is required to reach the highest peak of GABA within ‘*Ca*. L. asiaticus’-infected citrus plants.

Furthermore, to better understand the relationship between GABA supplementation and endogenous GABA levels, metabolome data were fitted using a simple linear regression (SLR) model (Fig. 7C and D). SLR showed that the endogenous GABA levels were slightly correlated with the exogenous dose of GABA in healthy (y = 2.747 + 0.024x, R^2^ = 0.3267, and *p* = 0.0028; Fig. 7C) and ‘*Ca*. L. asiaticus’-infected (y = 4.313 + 0.015x, R^2^ = 0.1003, and *p* < 0.0001; Fig. 7D) citrus plants. Additionally, to test the nonlinear phenomena between endogenous GABA levels and the exogenous GABA doses, data were fitted with a second-degree polynomial regression (SPR) model (Fig. 7C and D). Interestingly, the relationship between endogenous GABA levels and supplemented GABA doses followed a positive and quadratic model in both healthy (y = − 0.003 × ^2^ + 0.203x + 2.188, R^2^ = 0.7597, and *p* < 0.0001; Fig. 7C) and ‘*Ca*. L. asiaticus’-infected (y = − 0.005 × ^2^ + 0.257x + 3.558, R^2^ = 0.6862, and *p* < 0.0001; Fig. 7D).

#### GABA application induced the transcript levels of *CsgabP* in healthy and ‘*Ca*. L. asiaticus’-infected citrus plants

The effect of GABA supplementation on the gene expression of predicted *CsgabP* was investigated (Fig. 7E and F). Although the high concentration of GABA (5, 10, and 50 mM) significantly induced the transcript levels of *CsgabP* in citrus leaves (more than 3-folds), no significant differences were noticed between the lowest GABA concentration (1mM) and the mock-treated healthy plants (Fig. 7E). Likewise, the gene expression of predicted *CsgabP* in ‘*Ca*. L. asiaticus’-infected leaves recorded their highest levels when citrus plants were treated with 10 mM GABA (7.76-folds), followed by the highest dose of 50 mM GABA (6.35-folds) (Fig. 7F).

To better understand the relationship between *CsgabP* gene expression and exogenous GABA application, SLR and SPR were modeled. Briefly, a slight correlation was noticed between the relative gene expression of *CsgabP* and the exogenous GABA concentration (mM) in healthy citrus plants (y = 1.939 + 0.034x, R^2^ = 0.3863, and *p* = 0.0009; Fig. 7G). This correlation has been weakened in ‘*Ca*. L. asiaticus’-infected leaves (y = 5.065 + 0.035x, R^2^ = 0.1865, and *p* = 0.0311; Fig. 7H). Due to the nonlinear relationship between exogenous GABA concentrations and the transcript levels of *CsgabP*, both metabolic and transcriptomic data were fitted using the SPR model (Fig. 7G and H). Generally, the relationship between supplementary GABA doses and *CsgabP* expression followed a positive and quadratic model. The relationship between exogenous GABA concentrations and the transcript levels of *CsgabP* in healthy citrus plants is described by the equation y = − 0.005 × ^2^ + 0.289x + 1.123 (R^2^ = 0.8861, and *p* < 0.0001; Fig. 7G) while in ‘*Ca*. L. asiaticus’-infected plants it is described by y = − 0.009 × ^2^ + 0.479x + 3.678 (R^2^ = 0.8866, and *p* < 0.0001; Fig. 7H).

Additionally, to prove the correlation between endogenous GABA content and the gene expression of *CsgabP*, SLR and SPR were applied (Fig. 7I and J). Briefly, the linear regression of the endogenous GABA content versus the *CsgabP* expression showed a strong positive correlation between them in both healthy (R^2^ = 0.8776; Fig. 7I) and ‘*Ca*. L. asiaticus’-infected plants (R^2^ = 0.7391; Fig. 7J). Likewise, SPR models showed a positive and quadratic relationship between endogenous GABA levels and the transcript levels of *CsgabP* in both healthy (y = 0.739 × ^2^ − 2.828x + 3.661, R^2^ = 0.9275, and *p* < 0.0001; Fig. 7I) and ‘*Ca*. L. asiaticus’-infected plants (y = − 0.280 × ^2^ + 4.201x − 7.501, R^2^ = 0.7752, and *p* < 0.0001; Fig. 7J).

### *CsgabP* is involved in citrus response to different biotic stressors

#### ‘*Ca*. L. asiaticus’ infection altered the endogenous GABA content and its transporter gene, *CsgabP*, in citrus leaves

**Fig. 8:**
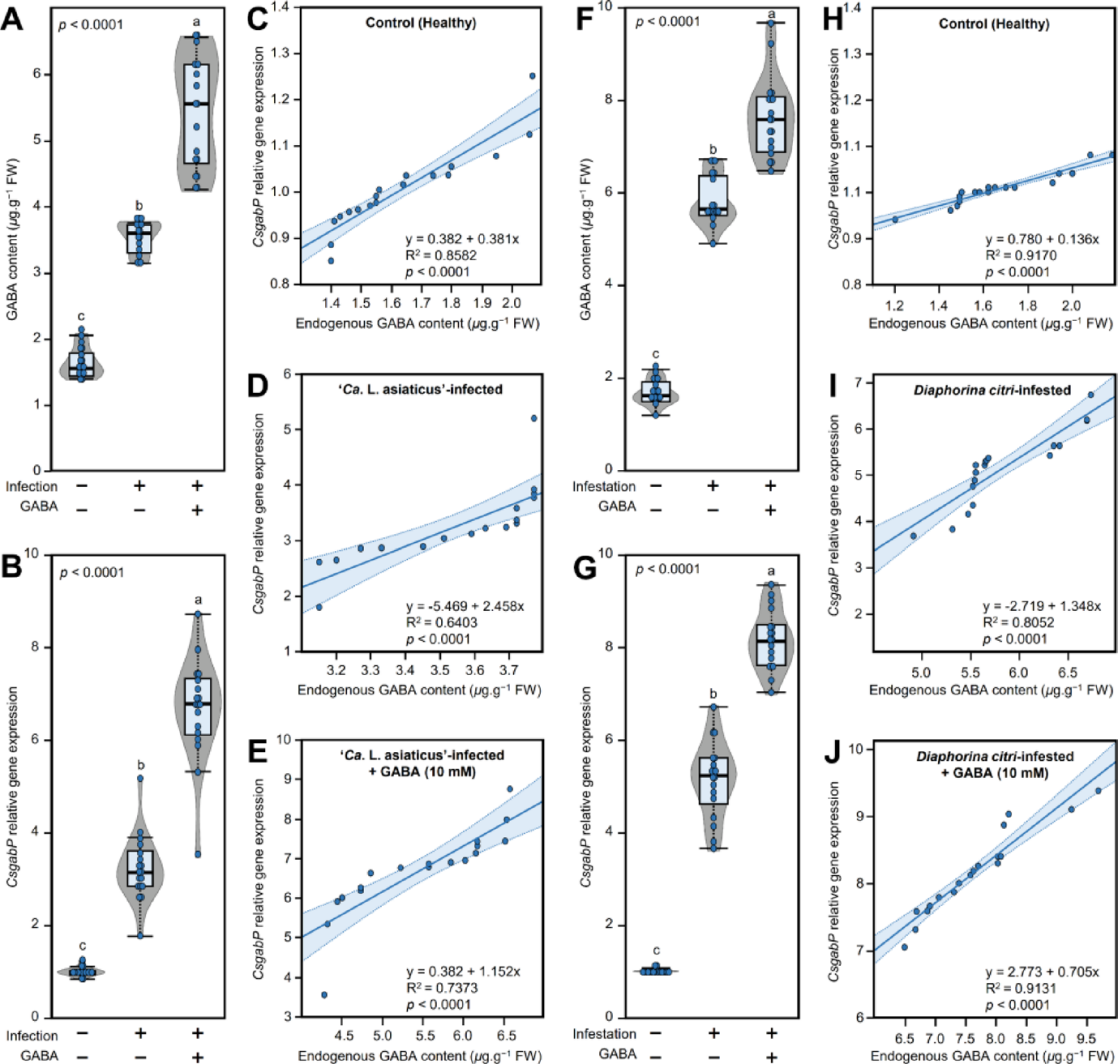
Effects of ‘*Ca*. L. asiaticus’ infection and *D. citri* infestation on the endogenous GABA content and its transport carrier *CsgabP* from Valencia sweet orange (*Citrus sinensis*). (**A** and **B**) Effect of ‘*Ca*. L. asiaticus’ infection and/or GABA supplementation on endogenous GABA levels (*µ*g.g^− 1^ FW) and *CsgabP* relative gene expression from Valencia sweet orange, respectively. (**C**,**D**, and **E**) Simple linear regression (SLR) analysis between endogenous GABA levels (*µ*g.g^− 1^ FW) and *CsgabP* relative gene expression from healthy, *Ca.* L. asiaticus’-infected, and ‘*Ca*. L. asiaticus’-infected + GABA (10 mM) Valencia sweet orange, respectively. (**F** and **G**) Effect of *D. citri* infestation and/or GABA supplementation on endogenous GABA levels (*µ*g.g^− 1^ FW) and *CsgabP* relative gene expression from Valencia sweet orange, respectively. (**H**,**I**, and **J**) Simple linear regression (SLR) analysis between endogenous GABA levels (*µ*g.g^− 1^ FW) and *CsgabP* relative gene expression from healthy, *D. citri*-infested, and *D. citri*-infested + GABA (10 mM) Valencia sweet orange, respectively. In boxplots, whiskers represent the minimum and the maximum values, while thick horizontal lines specify the median. Light-blue boxes show the interquartile ranges (25th to 75th percentile of the data), white dots represent the raw data (*n* = 6), and gray shading represents the corresponding violin plot. Different letters signify statistically significant differences among treatments, whereas the same letters indicate no significant differences among them using Tukey’s HSD (*p* < 0.05). In SLR models, blue dots represent raw data (*n* = 6). The fitted SLR line is presented as blue lines. The 95% confidence intervals for the estimated regression are shaded with the same color and edged by dotted lines. Regression equations for both SLR and SPR models, *R*^2^and *p*-value based on the *F* test (*p* ≤ 0.05) were also obtained and are presented within the graphs.

Targeted metabolomics showed that infection with ‘*Ca.* L. asiaticus’ significantly enhanced the accumulation of endogenous GABA content (3.54 ± 0.23 *µ*g.g^− 1^ FW) compared to the non-infected control plant (1.64 ± 0.22 *µ*g.g^− 1^ FW) (Fig. 8A). Moreover, GABA application further enhanced the GABA accumulation in ‘*Ca.* L. asiaticus’-infected plans (5.45 ± 0.83 *µ*g.g^− 1^ FW). Interestingly, the transcript levels of *CsgabP* had a similar profile. Relative *CsgabP* expression significantly upregulated upon infection with ‘*Ca.* L. asiaticus’ (3.23-folds) and almost doubled with infected plants upon GABA application (6.67-folds) (Fig. 8B).

To improve our comprehension of the relationship between endogenous GABA content and the transcript levels of its putative transporter, *CsgabP*, the SLR model was applied for all three studied cases including healthy control (Fig. 8C), ‘*Ca*. L. asiaticus’-infection (Fig. 8D), and ‘*Ca*. L. asiaticus’-infected + 10 mM GABA (Fig. 8E). Briefly, although a strong positive correlation (R^2^ = 0.8582) was observed between endogenous GABA content and relative *CsgabP* expression in the absence of both ‘*Ca*. L. asiaticus’ infection and GABA application (healthy control plants), the SLR analysis showed that the linear regression slope of the fitted line was very small (0.381; Fig. 8C). On the other hand, infection with ‘*Ca*. L. asiaticus’ significantly increased the slope of the fitted line (2.458 and *p* < 0.0001) and maintained a strong positive correlation (R^2^ = 0.6403) between endogenous GABA content and *CsgabP* expression (Fig. 8D). It is worth mentioning that the linear regression of the endogenous GABA content versus the relative expression of *CsgabP* showed that GABA application significantly strengthened the positive correlation between both variables (R^2^ = 0.7373 and *p* < 0.0001) and maintained an acceptable linear regression slope (y = 0.382 + 1.152x; Fig. 8E).

#### *D. citri* infestation enhanced the endogenous GABA levels and upregulated *CsgabP* in citrus leaves

GC-MS-SIM-based analysis showed that endogenous levels of GABA were significantly elevated under the infestation with *D. citri* (5.84 ± 0.54 *µ*g.g^− 1^ FW) compared to healthy control (1.68 ± 0.26 *µ*g.g^− 1^ FW) (Fig. 8F). Furthermore, the endogenous GABA accumulated more (7.64 ± 0.86 *µ*g.g^− 1^ FW) upon the application of 10 mM GABA to *D. citri*-infested Valencia sweet orange leaves. In agreement with these findings, *CsgabP* was upregulated in *D. citri*-infested plants (5.16-folds) and further enhanced upon GABA supplementation (8.16-folds) (Fig. 8G).

It is worth noting that the SLR confirmed the positive correlation between endogenous GABA content and the transcript levels of its putative transporter, *CsgabP* within healthy control (Fig. 8H), *D. citri*-infested (Fig. 8I), and *D. citri*-infested + 10 mM GABA (Fig. 8J) citrus plants. Although the healthy control plants (neither *D. citri* infestation nor GABA application) recorded the strongest correlation (R^2^ = 0.9170) between endogenous GABA content and *CsgabP* expression, it also recorded the lowest linear regression slope (y = 0.780 + 0.136x; Fig. 8H). Nevertheless, *D. citri* infestation significantly increased the linear regression slope (y = -2.719 + 1.348x and *p* < 0.0001) and maintained a strong correlation between GABA content and expression of *CsgabP* (R^2^ = 0.8052; Fig. 8I). Furthermore, the SLR showed that GABA supplementation significantly supported the positive correlation between GABA content and the transcript levels of *CsgabP* (y = 2.773 + 0.705x, R^2^ = 0.9131, and *p* < 0.0001; Fig. 8J). In other words, there was a significant positive dose-dependent relationship between the endogenous GABA content and the gene expression of its transporter *CsgabP*, which confirms the potential role of GABA against ‘*Ca.* L. asiaticus’ and its vector, *D. citri*, within HLB-affected trees.

## Discussion

The potential role(s) of GABA in plants is poorly studied, and its associated genes are not fully characterized across various plant species, particularly non-model plants. PhytoGABA is metabolized through a short metabolic route called GABA shunt^29,45,46^ that is essential for the central carbon and nitrogen metabolism^33,47^as well as response to several abiotic^45,46,48^ and biotic stress^18,49,50^ including bacterial pathogens^18,51^. GABA metabolism involves several evolutionarily conserved enzymes that bypass the α-Ketoglutarate-to‐succinate conversion outside the mitochondrial TCA cycle^16,18,34,52^. Briefly, GABA is biosynthesized in the cytosol from glutamate via an irreversible decarboxylation reaction driven by glutamate decarboxylase (GAD)^46^. Subsequently, cytosolic GABA is transported into the mitochondria via the activity of a mitochondrial *gabP*^16,18,34^transaminated to succinic semialdehyde by GABA transaminase (*gabT*), oxidized to succinate by succinate semialdehyde dehydrogenase (*SSADH*; also known as *gabD*)^46^and then succinate feeds into the TCA cycle. Although a mitochondrial *gabP* was reported previously to connect the GABA shunt and the TCA cycle in the model plant, *Arabidopsis thaliana*^16^unfortunately, specific GABA transporters in non-model plants such as citrus remain poorly characterized.

Previously, we proposed three *gab* genes to be involved in enabling the non-cyclic flux toward succinate via GABA Shunt in *C. sinensis*^34^. In silico genome-wide analysis showed that the citrus genome possesses three putative *gab* genes including three *CsgabP*, one *CsgabT*, and three isoforms of *CsgabD*^34^. *CsgabP* was predicted from two loci within chromosome 2. Locus LOC102610833 encodes for two isoforms of amino-acid (AA) permease BAT1-like; isoform X1 (*CsgabP*-1) and isoform X2 (*CsgabP*-2), whereas, LOC102627227 encodes for one AA permease BAT1^34^. Although the three *gabP* genes were highly similar to each other, LOC102627227 was discontinued and its associated record on NCBI was removed as a result of standard genome annotation processing. In the current study, we are refining our previous annotation, as well as providing a deeper in silico characterization, structural modeling, and molecular docking of *CsgabP*s of *C. sinensis.*

Herein, bioinformatics-based analysis showed that the citrus genome encodes for only two putative *CsgabP* proteins that were relatively highly homologous to each other and also homologous to BAT1 proteins from other plant species. In agreement with these findings, two GABA transporters (*gabP1* and *gabP2*) were reported previously in *Corynebacterium variabile* DSM 44,702^53^. It was reported previously that GABA uptake is mainly controlled by specific transporters of the amino acid/polyamine/organocation (APC) superfamily^54,55^. These transporters are conserved in fungi^56^ plants^16,57^, and animals^55^. Moreover, the physicochemical properties of putative *CsgabP* proteins from *C. sinensis* were relatively comparable to *AtgabP* from *A. thaliana* and those of other BAT1 proteins. For instance, the molecular mass (MW) of putative *CsgabP*s ranged from 55.33 to 56.07 kDa which was identical to the predicted MW of *AtgabP* (55.3 kDa)^16^and experimental evidence that a single 55-kDa polypeptide was noticed the mitochondrial fraction of protein extracts using antiserum specific to the N-terminus of *AtGABP*^16^.

Moreover, in silico analysis showed that putative *CsgabP*s proteins might be members of the previously described plant amino acid/polyamine transporter I family (InterPro entry: IPR002293) based on the Conserved Domain Database (CDD)^58,59^ blast search. The InterPro analysis suggested a high topological similarity between *CsgabP*-1 and *CsgabP*-2 which both had an amino acid/polyamine transporter I family. Furthermore, both proteins had an AA permease conserved site with four unintegrated sites including AA permease BAT1 homolog, AA permease BAT1, amino acid/polyamine transporter I, and AA transporter. BAT1 and CAT1 were reported previously as members of the APC superfamily^54,60^. Unlike other plant AA transporters, *BAT1* from *A. thaliana* (At2g01170) exhibited both export and import activity and thus was named bidirectional AA transporter 1 (*BAT1*)^57^. However, further studies are required to confirm the transport direction of predicted *CsgabP*s.

The localization and topology of transmembrane domains of both *CsgabP*s proteins using two methods of secondary structure predictions suggest the putative *CsgabP*s are highly hydrophobic integral transmembrane transporter proteins. The hydropathy profile of *CsgabP-*1 suggests the presence of 12 transmembrane regions with internal hydrophilic N- and C-terminal ends. However, because of the shorter AA sequence, *CsgabP-*2 is composed of only nine transmembrane domains flanked by hydrophilic N- and C-terminal domains. These findings are in agreement with the transmembrane topology of *gabP* from *Escherichia coli*^61^*Bacillus subtilis*^62^and *AtgabP* from *A. thaliana*^16^. Several other transporter proteins are evolutionarily related and similarly structured^63,64^. For instance, the primary structure prediction of four transport proteins of *Saccharomyces cerevisiae* included the uracil permease (*FUR4*), purine-cytosine permease (*FCY2*), arginine permease (*CAN1*), and the histidine permease (*HIP1*) suggests the presence of 9–12 transmembrane regions in each polypeptide chain^63^. Likewise, proline permease (*PUT*4) from *S. cerevisiae* was predicted as a hydrophobic protein with 12 transmembrane segments^64^.

Additionally, previous studies suggest that *gabP* possesses a “Consensus Amphiphatic Region (CAR)” sound to be evolutionarily conserved within this transport family^65^. CAR is positioned between helix 8 and loop 8–9, establishing a potential channel domain, and is suggested to be involved in ligand recognition and translocation^65^. Molecular docking analysis showed that GABA and its subsequent mitochondrial metabolites, succinic semialdehyde and succinic acid slightly interact with both *CsgabP*s via forming 1–3 conventional hydrogen bonds with several AA residues. These findings suggest their ability to bind the active site(s) of the predicted protein structures. Moreover, these findings suggest that the residues involved in GABA binding are conserved between *CsgabP*-1 and *CsgabP*-2, but with slight deportation due to the variation in the AA sequence length. Molecular docking studies previously showed that that GABA binds *MsGabP* from *Mycobacterium smegmatis* and its homologue from *M. tuberculosis* (*MtgabP*) in the same manner suggesting that the AA residues involved in GABA binding are conserved between *MsGabP* and *MtbGabP*^66^. It is worth mentioning that molecular docking provides a preliminary and tentative assessment of ligand-protein interactions rather than definitive binding confirmation. Therefore, further experimental studies are required to validate these findings.

Additionally, our molecular docking studies exhibited weak binding energies (around − 4 kcal/mol) between predicted *CsgabPs* and three ligands (GABA, succinic semialdehyde, and succinic acid) suggesting low-affinity interactions under physiological conditions^67^however, they do not necessarily reflect actual biological relevance. Protein-ligand docking depends on static structural models, assuming a rigid protein and ligand conformation^68^which does not fully describe the flexibility and dynamic nature of molecular interactions^69^. Accordingly, further validation through molecular dynamics simulations, experimental assays, and free energy calculations is required.

Predictive *CsgabP* proteins showed remarkably high homology (80–100% identity with 92–100% query cover) with APC transporters from other plant species. Although some were described as hypothetical proteins, most of these transporters were annotated as AA permease BAT1. Considering only the previously characterized *AtgabP* homologs, both *CsgabP*s showed more than 80% identity with *AtgabP A. thaliana*^16^ which has been reported previously to be GABA-specific permease. Moreover, AlphaFold is a computational approach that can regularly predict protein structures to near experimental accuracy even without similar known structures^41,42^. Interestingly, the AlphaFold2-based crystallographic 3D structures of putative *CsgabP* proteins revealed their overall cylindrical shape, intracellular N- and C-termini, short cytoplasmic and extracellular loops, and 9–12 transmembrane segments when predicted using the crystal structure of GadC from *E. coli* (PDB ID: 4dji.1.A). It is worth mentioning that crystallographic 3D structures of putative *CsgabP* proteins showed high similarity with other members of the APC superfamily such as Na + independent amino acid transporter (*ApcT*)^70^ Arginine/agmatine antiporter (*adiC*)^71^ and glutamate-GABA antiporter (*GadC*)^72^ from *E. coli*.

In the current study, we used SWISS-MODEL-based protein structure homology modeling^73^as well as an AI-based system AlphaFold2^74^ to predict the 3D structure of both *CsgabPs*. Regardless of the accuracy of experimental methods, sequence-based computational prediction of 3D structures has become more accessible compared to experimental approaches which are often time- and resources-consuming^75^. However, computational characterization and modeling of dynamic membrane transporters based on a single static model is challenging and presents several limitations^76–78^. It was reported previously that membrane transporters depend on diverse conformational transitions for their function^78^ which a single static model cannot capture, leading to an incomplete understanding of their function.

Moreover, membrane transporters often exist in distinct conformations, including inward-open, occluded, and outward-open^79,80^. Nevertheless, a single structure may not represent the acceptable conformation in which the substrate-binding site is accessible to the extracellular and intracellular regions^80^. This might negatively affect the interaction predictions, as ligand docking studies using a fixed structure may misidentify key binding sites or overlook biologically relevant sites. Additionally, transporters control the passage of solutes across tight lipid bilayers^81^where membrane organization modulates their structure and function, yet static models, particularly those predicted by AlphaFold, do not account for these environmental factors. To overcome these limitations, using multiple static models for dynamic transporters is recommended. Moreover, Further studies on molecular dynamics simulations, cryo-EM studies, and functional assays to validate computational predictions are required.

Proteins do not function individually within the plant cell, however, they perform in a network^82^. Predicted *CsgabP* proteins displayed distinct functional characteristics that feature their protein-protein interactions (PPI). STRING-12-based PPI showed that putative *CsgabP*s interact with 10 proteins that are mainly involved in the GABA shunt, glutamine metabolism, and arginine catabolism. Metabolic-engineered microorganisms proved that gabPs are involved in the metabolism and transport of AA-related compounds, including glutamate, GABA, and lysine^83^. Additionally, it was suggested that *gabP*s play a regulatory role in GABA biosynthesis-associated pathways such as the glutarate catabolism and the putrescine pathway for GABA anabolism^83^. The predicted PPI may clarify this regulatory role. Moreover, STRING-11-based PPI showed that putative *CsgabP*s interact with several superoxide dismutases (SODs) and SOD-Fe. SODs have strong antioxidative properties and are involved in reducing O_2_^·−^ to H_2_O_2_ to protect the cell from free radical damage. Recently, we showed that GABA accumulation contributes to citrus response via modulation of multiple metabolic pathways and redox status^19^. Moreover, GABA upregulated four SODs genes, including *CsSOD*-Cu/Zn, *CsSOD*-Mn, *CsSOD*-Fe, and *CsSOD*-Fe3^19^. Collectively, these findings suggest that *CsgabP*s might be involved in citrus response(s) via interacting with GABA-associated metabolic pathways, as well as, modulating the cellular redox homeostasis by interaction with SODs.

Furthermore, our findings showed that *CsgabP* was positively correlated with GABA accumulation in citrus plants. Briefly, GABA supplementation significantly enhanced the accumulation of endogenous GABA and upregulated *CsgabP* genes in healthy and ‘*Ca*. L. asiaticus’-infected citrus plants. Likewise, GABA and its transporter gene, *CsgabP*, were involved in citrus response to different biotic stressors including infection with the bacterial phytopathogen ‘*Ca*. L. asiaticus’ or the infestation with its vector, *D. citri*. The protective role(s) of GABA in citrus against ‘*Ca*. L. asiaticus’ might be due to the rapid metabolization to succinate and feed the TCA cycle^84^induction of phytohormones levels^20,85^modulation of GABA-related metabolic pathways^19,86^and maintaining the cellular redox homeostasis^19,87^.

**Fig. 9:**
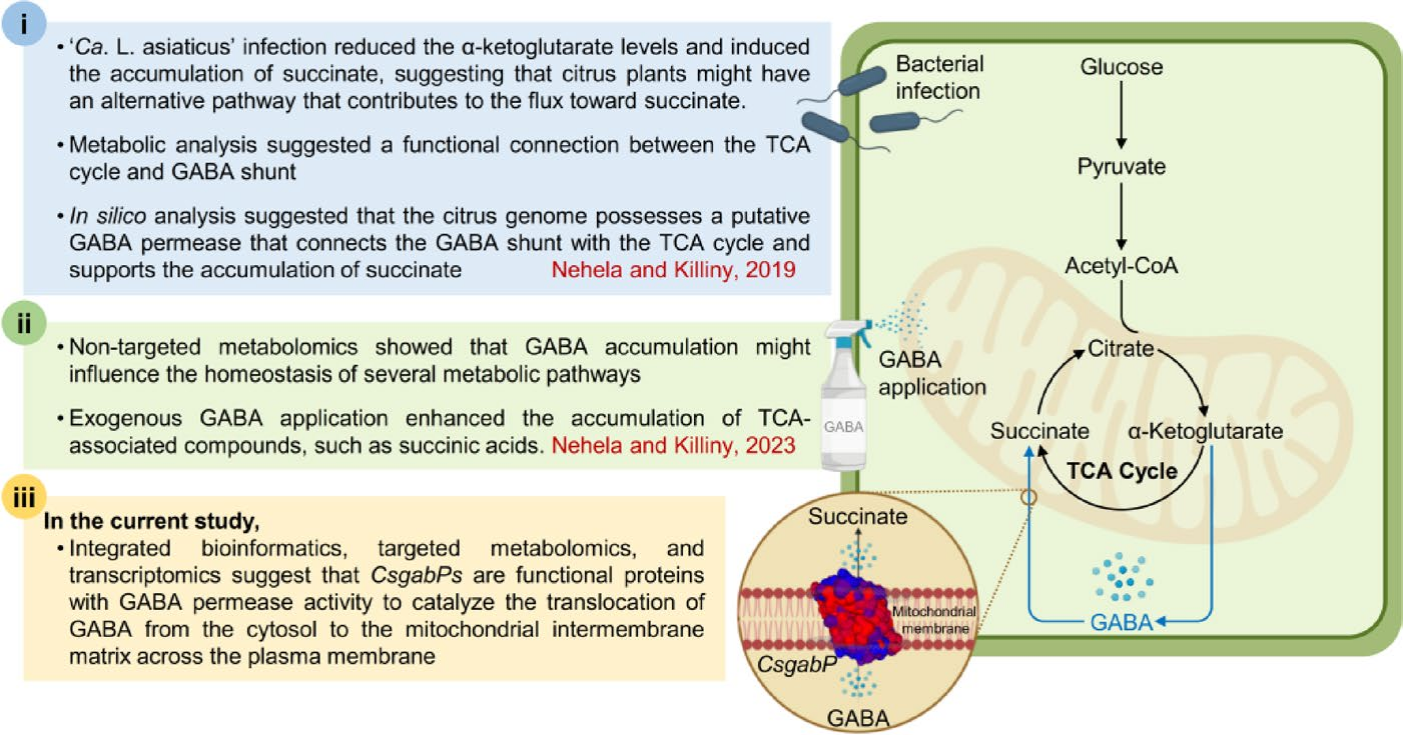
Hypothetical model of the potential role(s) of *CsgabP* from the non-model plant *Citrus sinensis* in GABA uptake under biotic stress. (i) the effect of ‘*Ca*. L. asiaticus’ infection on the TCA-related compounds, as well as the association between the GABA shunt and the TCA cycle in *C. sinensis* as our previous study^18^(ii) the effect of exogenous GABA application on TCA-related compounds as in our previous study^19^and (iii) the novelty of *CsgabP* characterization, as well as the unique contribution of *CsgabP* in GABA uptake and plant response to biotic stress form the current study.

In conclusion, aligned with our previous hypothesis that both the TCA cycle and GABA shunt are functionally connected^18^as well as the previously proposed role of GABA as a modulator of multiple metabolic pathways in *C. sinensis* response against ‘*Ca*. L.Asiaticus’^19^integrated bioinformatics, targeted metabolomics, and transcriptomics suggest that *CsgabP*s are potential GABA transport carriers with GABA permease activity to catalyze the translocation of GABA from the cytosol to the mitochondrial intermembrane matrix across the plasma membrane (Fig. 9). The predicted primary structure of *CsgabP*s supports their GABA permease activity. Moreover, secondary and 3D structures of predicted *CsgabP* proteins and their high homology with other permeases from bacteria, fungi, and *AtgabP* form *A. thaliana* also characterize their amino acid or GABA permease activity. Additionally, the topology of putative *CsgabP*s as integral transmembrane transporter proteins, as well as molecular docking studies, supports their potential role in GABA uptake. Moreover, our findings showed that *CsgabP* was positively correlated with GABA accumulation in healthy citrus plants, as well as within those infected with ‘*Ca*. L. asiaticus’ or infested with its vector *D. citri* suggesting their potential role in citrus defensive response(s) to biotic stress. However, further experimental validation through functional analysis is required. The importance of this study is not only to characterize the GABA transport carrier, *CsgabP*, from *C. sinensis* as an integral membrane transporter that might be involved in facilitating GABA translocation and contributes to cellular metabolism but also to understand better the functional connection between the GABA shunt and the TCA cycle. This connection was poorly reported previously in non-model plants. Finally, our findings add another piece to the puzzle to build a comprehensive picture of citrus defense responses against HLB, which is essential for discovering novel sustainable management strategies for HLB.

## Materials and methods

### In silico snalysis

In silico analysis was carried out based on recently available data about the *C. sinensis* genome on the two major databases included the “*Citrus sinensis* CDS, phytozome 154 v1.1” and “*Citrus sinensis proteins*, phytozome 154 v1.1” BLAST datasets^88^ and their corresponding gene sequences based on the most recent available data in GenBank, National Center for Biotechnology Information website (NCBI) (for more information, see Method S1.1). In silico analyses included Protein-protein BLAST (BLASTp) and Nucleotide-Nucleotide BLAST (BLASTn)^35,89^ to retrieve the amino acid (AA) sequence homologies that resemble the query amino-acid sequence of bidirectional amino acid transporter 1 (BAT1; GenBank accession no. NP_565254.1; 516 aa; aka GABA permease [*AtgabP* ])^16^ from *Arabidopsis thaliana* (for more information, see Method S1.2). Multiple protein sequence alignment analysis was done using the Constraint-Based Alignment Tool (COBALT; for multiple protein sequences)^90^and ClustalW to align the top matched AA sequences of putative *CsgabP* proteins from *C. sinensis*^91^. Detailed information is described in “Supplementary Information” (Method S1.3).

Evolutionary analysis and phylogenetic trees of the predicted *gabP* genes from sweet orange (*C. sinensis*) and their matched sequences from other plant species were inferred using the maximum likelihood method and Jones-Taylor-Thornton (JTT) matrix-based model^37^ (for more information, see Method S1.4). Likewise, the theoretical physicochemical properties of putative *CsgabP* proteins were computed using Expasy’s ProtParam tool^92^. For more details, see the “Supplementary Information” (Method S1.5).

Additionally, primary structure analysis and conserved domains were discovered using Multiple Em for Motif Elicitation (MEME) Suite-version 5.5.7^40^ and classified into families using the InterPro tool^93^ (for more information, see Method S1.6). Likewise, secondary structure analysis was initially analyzed using the Network Protein Sequence Analysis (NPS@) server^94,95^ (for more information, see Method S1.7). Moreover, the secondary motif map and topology diagram were calculated using the PDBsum Pictorial database^96^. Finally, crystallographic tertiary structure analysis and three-dimensional (3D) modeling were carried out using The SWISS-MODEL server^97^then further confirmed using the AlphaFold2 model^41,42^ based on ColabFold^43^. The predicted 3D structures (PDB format) were interactively visualized using the UCSF-Chimera package^98^ (for more information, see Method S1.8).

Localization and topology of transmembrane domains were recognized using the Protein Homology/analogY Recognition Engine (Phyre2) server^99^ and further confirmed using the TMHMM-2.0 server (Method S1.9). Protein-protein interaction was predicted using STRING 11.0 and STRING 12.0^44^ (for more information, see Method S1.10). Finally, the possible affinity between ligand molecules (GABA, succinic semialdehyde, and succinic acid), and predictive *CsgabP*s target proteins was evaluated via molecular docking using Autodock Vina 1.5.7 software^100^. For more information, see the “Supplementary Information” (Method S2).

### Greenhouse experiments

To better understand the relationship between predicted *CsgabP*s and endogenous GABA levels within citrus plants, multi-omics techniques including metabolomics and transcriptomics were used. Briefly, the effect of different biotic stress (infection with ‘*Ca*. L. asiaticus’ and infestation with *D. citri*), as well as the consequence of exogenous GABA supplementation on the endogenous GABA levels, as well as the expression of the *CsgabP* gene was investigated under greenhouse conditions. Detailed description of plant materials, growth conditions, leaf sampling (Method S3.1), preparation of ‘*Ca.* L. asiaticus’-Infected plants (Method S3.2), preparation of *D. citri*-Infested plants (Method S3.2), and exogenous GABA application (Method S3.3) are described within the “Supplementary Information”.

### Targeted metabolomic analysis of GABA using GC-MS-SIM

GABA was extracted in tripartite from approximately 100 ± 2 mg leaf tissue using 750 µL of acidic methanol 80% as described in our previous studies^18,28,101,102^. The supernatants were collected, combined together, concentrated to 50 µL, and then derivatized using methyl chloroformate (MCF) following the protocol of^24^ with slight modifications as described in our previous studies^18,28,101,102^. For GC-MS analysis, 1 µL of the derivatized samples was injected into a GC-MS system model Clarus 680 (Perkin Elmer, Waltham, MA, USA) using the same chromatographic conditions as described in our previous study^18–20,28^. GABA was identified by comparing their retention times (RT), linear retention indices (LRIs), and mass spectra with those of authentic reference standards (Sigma-Aldrich, St. Louis, MO, USA) treated identically to samples. Endogenous GABA levels were quantified using a standard curve of serial GABA dilutions (0, 5, 10, 25, and 50 mg.L^− 1^) derivatized and treated identically to samples^19,20^.

### Gene expression analysis using RT-qPCR

Total genomic RNA was extracted using TriZol^®^ reagent (Ambion^®^, Life Technologies, NY, USA) as described in our previous studies^18,20,28^ to determine the transcript levels of *CsgabP* genes under different biotic stressors (‘*Ca*. L. asiaticus’-infected and *D. citri*-infested) and/or GABA supplementation. cDNA was synthesized using a SuperScript first-strand synthesis system (Invitrogen) with random hexamer primers (Table S1) as described by the manufacturer’s instructions. The 2^−ΔΔ*C*^_T_ method was used to determine the relative changes in gene expression among PCR products^103^. Four housekeeping (reference genes) genes were used for the normalization of gene expression including *CsEF-1α*, *CsF-box*, *CsGAPC1*, and *CsSAND*^104,105^.

### Statistical analysis

Throughout this study, all experiments were laid out using a completely randomized design with six biological replicates ( 3 trees per replicate) and analyzed in duplicates (two technical replicates for each). ANOVA was used to determine differences between treatments, followed by Tukey’s HSD for post-hoc pairwise comparisons. Simple linear regression (SLR) and second-degree polynomial regression model (quadratic model) were performed to model the relationship between studied variables. For more information, see the “Supplementary Information” (Method S4).

## Electronic supplementary material

Below is the link to the electronic supplementary material.

Supplementary Material 1

## Data Availability

All relevant data supporting this study’s findings are presented in the figures of this manuscript and/or can be found in the supporting materials.
