# Supplementary material for: In silico characterization, structural modeling, and molecular docking of GabP in citrus and its potential role in GABA uptake
Source: Sci Rep. 2025 Jul 4;15:23919. doi: 10.1038/s41598-025-07447-y (PMC12227542; doi:10.1038/s41598-025-07447-y)
Supplement: Supplementary file 1 — Supplementary Material 1 [file 41598_2025_7447_MOESM1_ESM.docx]

***Supplementary Informaion***

## Not Just a Cycle: *In Silico* Characterization, Structural Modeling, and Molecular Docking of *gabP* from a Non-model Plant and its Potential Role in GABA Uptake in Plants under Biotic Stress

**Yasser Nehela ^1^ and Nabil Killiny ^2,^***

^1^ Department of Agricultural Botany, Faculty of Agriculture, Tanta University, Tanta, Egypt

^2^ Department of Plant Pathology, Citrus Research and Education Center, University of Florida, 700 Experiment Station Rd., Lake Alfred, FL 33850

*** Corresponding author**: Nabil Killiny

Email: [nabilkilliny@ufl.edu](mailto:nabilkilliny@ufl.edu)

Phone: 863-956-8833

# ORCID

Nabil Killiny: <https://orcid.org/0000-0002-3895-6943>

Yasser Nehela: <https://orcid.org/0000-0003-1177-8099>

**Keywords**: Huanglongbing; citrus greening; Liberibacter; citrus; TCA cycle; GABA shunt; *gab* genes; GABA permease (*gabP*); *in silico* analysis

# Supplementary Materials and Methods

## Method S1. *In silico* Analysis

### **Method S1.1.** **Sequence retrieval and databases**

*In silico* analysis was carried out based on recently available data about the *C. sinensis* genome on the two major databases included the "*Citrus sinensis* CDS, phytozome 154 v1.1" and "*Citrus sinensis proteins*, phytozome 154 v1.1" BLAST datasets available on Citrus Greening Solutions website (<https://citrusgreening.org/organism/Citrus_sinensis/genome>, accessed on October, 2^nd^, 2024) ^1^ and their corresponding gene sequences based on the most recent available data in GenBank, National Center for Biotechnology Information website (NCBI, <http://www.ncbi.nlm.nih.gov/gene/>). Subsequently, a shortlist of the top matched proteins was generated based on the evolutionary relationships within the phylogenetic trees, identifying more than 50%, and excluding all the hypothetical, partial, and low-quality proteins that have these characteristics, as well as obsolete records that were removed as a result of standard genome annotation processing.

### **Method S1.2.** **Protein-protein BLAST (BLASTp) and Nucleotide-Nucleotide BLAST (BLASTn)**

Two Basic Local Alignment Search Tool (BLAST) algorithms were used throughout the study. The protein-protein BLAST (BLASTp) algorithm was used for the genome-wide analysis of putative *CsgabP* proteins from *C. sinensis* to retrieve the amino acid (AA) sequence homologies that resemble the query amino-acid sequence. The AA sequence of bidirectional amino acid transporter 1 (BAT1; GenBank accession no. [NP_565254.1](https://www.ncbi.nlm.nih.gov/protein/NP_565254.1); 516 aa; aka GABA permease [*AtgabP* ]) ^2^ from *Arabidopsis thaliana* were used as query sequences to match their homologous proteins from sweet orange (*C. sinensis*) using the protein-protein BLAST (BLASTp 2.8.0+) using the compositionally adjusted substitution matrices ^3,4^. On the other hand, the nucleotide-nucleotide BLAST (BLASTn) algorithm was used to retrieve the nucleotide sequence for the top-matched sequences producing significant alignments with query sequences.

### **Method S1.3.** **Multiple Protein Sequence Alignment Analysis**

The AA sequences of the predicted *gabP* genes from sweet orange (*C. sinensis*) and their matched sequences from other plant species were simultaneously aligned using the Constraint-Based Alignment Tool (COBALT; for multiple protein sequences (<https://www.ncbi.nlm.nih.gov/tools/cobalt/re_cobalt.cgi>, accessed on October, 2^nd^, 2024) ^5^. Besides, ClustalW (<http://www.genome.jp/tools-bin/clustalw>, accessed on October, 2^nd^, 2024) was used to align the top matched AA sequences of putative *CsgabP* proteins from *C. sinensis* ^6^. Moreover, the Clustal Omega algorithm (<https://www.ebi.ac.uk/jdispatcher/msa/clustalo> ) ^7^ and QIAGEN CLC Genomics Workbench 24.0.2 were used for the multiple sequence alignment and visualization of retrieved AA sequences. Conserved amino acids and similar residues are shaded using RasMol coloring.

### **Method S1.4.** **Evolutionary Analysis and Phylogenetic Trees**

The evolutionary history of the predicted *gabP* genes from sweet orange (*C. sinensis*) and their matched sequences from other plant species was inferred using the maximum likelihood method and Jones-Taylor-Thornton (JTT) matrix-based model ^8^. The initial tree for the heuristic search was obtained automatically by applying Neighbor-Join and BioNJ algorithms to a matrix of pairwise distances estimated using the JTT model and then selecting the topology with a superior log-likelihood value. Evolutionary analyses were conducted in MEGA X ^9^.

### **Method S1.5.** **Physicochemical properties of putative *CsgabP* proteins**

The theoretical physicochemical properties of putative *CsgabP* proteins were computed using Expasy’s ProtParam tool (<https://web.expasy.org/protparam/>, accessed on October, 2^nd^, 2024) ^10^. These physicochemical properties included, theoretical isoelectric point (pI), molecular weight (MW), extinction coefficients (ε), instability index (II), aliphatic index, and grand average of hydropathicity (GRAVY). The extinction coefficient (ε; M^-1^ cm^-1^) was calculated based on the potential light absorbance by the protein at 280 nm measured in water. Moreover, II was determined to estimate the stability of putative *CsgabP* proteins. The protein was predicted to be stable when II is smaller than 40, however, the protein was expected to be unstable when the II value is above 40 ^10^. GRAVY values of putative *CsgabP* proteins were calculated as the sum of hydropathy values of all the amino acids, divided by the number of residues in the sequence.

### **Method S1.6.** **Primary structure analysis and Conserved domains**

Novel motifs within the unaligned *CsgabP* protein sequences from sweet orange (*C. sinensis*), along with the query *AtgabP* protein sequence, were discovered using Multiple Em for Motif Elicitation (MEME) Suite-version 5.5.7 (<http://meme.sdsc.edu/meme/meme.html>, accessed on October, 2^nd^, 2024) ^11^. Moreover, functionally important domains and conserved sites of the predicted *CsgabP* genes from *C. sinensis* were interactively classified into families using the InterPro tool (<https://www.ebi.ac.uk/interpro/>, accessed on October, 2^nd^, 2024) ^12^.

### **Method S1.7.** **Secondary structure analysis**

Secondary structures of predicted *CsgabP* proteins, as well as, the query AtgabP protein were initially analyzed using the Network Protein Sequence Analysis (NPS@) server ^13^ based on an improved self-optimized prediction method (SOPMA) ^14^ (<https://npsa-prabi.ibcp.fr/cgi-bin/npsa_automat.pl?page=/NPSA/npsa_sopma_f.html>, accessed on October, 4^th^, 2024). Moreover, the secondary motif map and topology diagram were calculated using the PDBsum Pictorial database (<http://www.ebi.ac.uk/thornton-srv/databases/pdbsum/Generate.html>, accessed on October, 4^th^, 2024) ^15^. The protein secondary structure motifs shown in PDBsum are computed using v.3.0 of Gail Hutchinson's PROMOTIF program. Helices, helix-helix interactions, beta turns, and gamma turns of the 3D structure of *CsgabP*s were also predicted using the PDBsum Pictorial database. A beta turn is defined for 4 consecutive residues (denoted by i, i+1, i+2, and i+3) if the distance between the C-alpha atom of residue i and the C-alpha atom of residue i+3 is less than 7Å and if the central two residues are not helical (either using the Kabsch and Sander criteria or using author-defined criteria) ^16^. A Gamma turn is defined for 3 residues i, i+1, i+2 if a hydrogen bond exists between residues i and i+2 and the phi and psi angles of residue i+1 fall within 40 degrees of one of the following 2 classes ^17,18^: Classic type (phi_(i+1)_= 75.0 and psi_(i+1)_= -64.0) and Inverse type (phi_(i+1)_= -79.0 and psi_(i+1)_= 69.0).

### **Method S1.8.** **Tertiary structure analysis and three-dimensional (3D) modeling**

The protein structure homology modeling, structure-based function annotation, three-dimensional (3D) structure prediction, and the membrane topology of the predicted *CsgabP* proteins from sweet orange (*C. sinensis*) were carried out using The SWISS-MODEL server (<https://swissmodel.expasy.org/>, accessed on October, 2^nd^, 2024) ^19^. The predicted 3D structures (PDB format) were interactively visualized using the UCSF-Chimera package (version 1.15; <https://www.cgl.ucsf.edu/chimera/>, accessed on October, 2^nd^, 2024) ^20^. The 3D structures prediction of both *CsgabP* proteins were further confirmed using the AlphaFold2 ^21,22^ model (<https://neurosnap.ai/service/AlphaFold2>, accessed on October, 9^th^, 2024) based on ColabFold ^23^ using the multiple sequence alignment (MSA) mode of "mmseqs2_uniref_env” of five cycles and recycles early stop tolerance of 0.75.

### **Method S1.9.** **Localization and topology of transmembrane domains**

Because both putative *CsgabP* proteins are related to a well-characterized family known to contain transmembranes, the optimal membrane location for each transmembrane biounit of both *CsgabP* proteins (*CsgabP*-1 and *CsgabP*-2) was recognized using Protein Homology/analogY Recognition Engine (Phyre2-V 2.0) server (<http://www.sbg.bio.ic.ac.uk/phyre2/html/page.cgi?id=index>, accessed on October, 2^nd^, 2024) based on the memsat-svm algorithm ^24^. Moreover, the prediction of the transmembrane helices of both *CsgabP* proteins was further confirmed using the TMHMM - 2.0 server (<https://services.healthtech.dtu.dk/services/TMHMM-2.0/>, accessed on October, 2^nd^, 2024) based on the Hidden Markov Model (HMM) ^25^.

### **Method S1.10.** **Protein-protein interaction analysis**

Protein-protein interaction was predicted using STRING 11.0 (<https://version-11-0.string-db.org/> accessed on May, 15^th^, 2020) and STRING 12.0 (<https://string-db.org/>, accessed on October, 2^nd^, 2024) ^26^ to integrate all known and predicted interactions between proteins. The integrated interactions include direct (physical) and indirect (functional) associations. The minimum required interaction score was preset to 0.2.

## Method S2. Molecular docking

The possible affinity between ligand molecules (GABA, succinic semialdehyde, and succinic acid), and predictive *CsgabP*s target proteins was evaluated via molecular docking. Initially, target protein structures were prepared by removing water molecules from the complexes ^27^, and then the crystallographic disorders and unfilled valence atoms were corrected. Subsequently, protein structure energy was minimized by applying the Chemistry at Harvard Macromolecular Mechanics (CHARMM) force fields ^28^. Additionally, 2D structures of ligand compounds were drawn using ChemBioDraw Ultra 16.0 (<https://chembiodraw-ultra.software.informer.com/>) and saved as a Spatial Data File (SDF) file, then the 3D structure was protonated, and 0.1 RMSD kcal/mole energy was minimized by the Merck molecular force field (MMFF94) force field. The minimized structure was prepared for docking via the ligand preparation tools. Autodock Vina 1.5.7 software was used for the docking process ^29^. The receptor was held rigid while the ligands were allowed to be flexible. During the refinement, each molecule was allowed to produce twenty different poses with the proteins. The docking scores (affinity energy) of the best-fitted poses with the active sites were recorded, and then 2D and 3D were visualized using the Discovery Studio visualizer (<https://www.3ds.com/products/biovia/discovery-studio>).

## Method S3. Greenhouse Experiments and Multi-omics

To better understand the relationship between predicted *CsgabP*s and endogenous GABA levels within citrus plants, multi-omics techniques including metabolomics and transcriptomics were used. Briefly, the effect of different biotic stress (infection with ‘*Ca*. L. asiaticus’ and infestation with *D. citri*), as well as the consequence of exogenous GABA supplementation on the endogenous GABA levels, as well as the expression of the *CsgabP* gene was investigated under greenhouse conditions.

### **Method S3.1.** **Plant materials, growth conditions, and leaf sampling**

The HLB-susceptible cultivar, ‘Valencia’ sweet orange (*Citrus sinensis* (L.) Osbeck), was used as an experimental plant throughout this study. All plants were approximately 100 ± 5 cm in height and 18-months old at the sampling time. Trees were maintained in a secured greenhouse at the Citrus Research and Education Center, University of Florida (CREC-UF), Lake Alfred, Florida, at 27 ± 2 °C, with 70 ± 3 % RH, and 16:8 h L/D photocycle. Citrus trees were watered twice weekly and fertilized monthly with a water-soluble 20:10:20 NPK fertilizer (Allentown, PA, USA). All experiments were laid out using a completely randomized design with six biological replicates (3 trees per replicate) and analyzed in duplicates (two technical replicates for each). For sampling, three leaves were collected per tree from different positions (top, middle, and lower part of the plant) and ages including juvenile, intermediate-aged (fully expanded but not hardened), and mature leaves (deep green and hardened). Because metabolite profiles fluctuate based on day and night cycles, all samples for metabolomics and transcriptomics studies were collected in the morning (~ 10:00 am) from greenhouse-grown plants to minimize variations due to diurnal changes. The collected leaves were chopped, mixed together, and immediately kept at −80°C until further analysis.

### **Method S3.2.** **Preparation of ‘*Ca.* L. asiaticus’-Infected and *D. citri*-Infested plants**

To obtain the ‘*Ca*. L. asiaticus’-infected plant materials, 12-month-old healthy plants were inoculated via bud grafting using ‘*Ca*. L. asiaticus’-positive materials and maintained under the same conditions described above. Upon initial symptom development and about six months post-inoculation (mpi), the presence of ‘*Ca*. L. asiaticus’ was confirmed using PCR according to ^30^. Healthy plants were prepared in the same way but using ‘*Ca*. L. asiaticus’-free materials ^31–33^.

To obtain both *D. citri-*infested citrus plants, 100 ‘*Ca*. L. asiaticus’-free *D. citri* adults (obtained from our laboratory colony reared on *Bergera koenegii,* nonhost for ‘*Ca.* L. asiaticus’) were transferred to 16-month-old healthy citrus plants with new flushes caged individually using insect-rearing cages (60 by 60 by 90 cm) and maintained in the growth room under the same conditions as described above ^31^.

### **Method S3.3.** **Exogenous GABA application**

To investigate the effect of exogenous GABA supplementation on endogenous GABA levels and the expression of *CsgabP,* 18-month-old citrus trees (healthy versus ‘*Ca.* L. asiaticus’-infected) were treated with 300 mL of 10 mM aqueous GABA solution (Sigma-Aldrich, St. Louis, MO, USA) per tree via soil drench method under low light intensity to avoid any degradation of GABA ^32–35^. The applied aliquot GABA solution was enough to fulfill the soil field capacity, however, the solution that exceeded the soil field capacity, and gravitationally drained from the pot bottom, was collected using a plastic plate and reapplied to the plant. Distilled water was used as a negative control. Both healthy and ‘*Ca.* L. asiaticus’-infected plants (GABA-treated vs. mock-treated) were maintained under the same conditions described above for seven days ^34,35^ before leaf sampling as described above.

## Method S4. Statistical analysis

Throughout this study, all experiments were laid out using a completely randomized design with six biological replicates (3 trees per replicate) and analyzed in duplicates (two technical replicates for each). The technical replicates were used only to test reproducibility and variability in the extraction protocol and GC-MS machine but were not used for statistical analysis to avoid the possibility of pseudo-replication. Analysis of Variance (ANOVA) test was used to determine differences between treatments, followed by Tukey's honestly significant difference test (Tukey's HSD) for post-hoc pairwise comparisons. Simple linear regression (SLR) was performed to model the relationship between exogenous GABA concentration (as an independent variable) and the endogenous GABA content or *CsgabP* relative gene expression (as dependent variables), as well as to model the correlation between endogenous GABA content (as an independent variable) and *CsgabP* relative gene expression (as dependent variables). Linear equation, R ^2^, and P value based on the F test (P ≤ 0.05) were obtained. Furthermore, due to the observed nonlinear phenomena between exogenous GABA concentrations and the endogenous GABA content or *CsgabP* relative gene expression, data were fitted with a second-degree polynomial regression model (quadratic model). Quadratic equation, R ^2^, and P value based on the F test (P ≤ 0.05) were obtained.

# Supplementary Figures


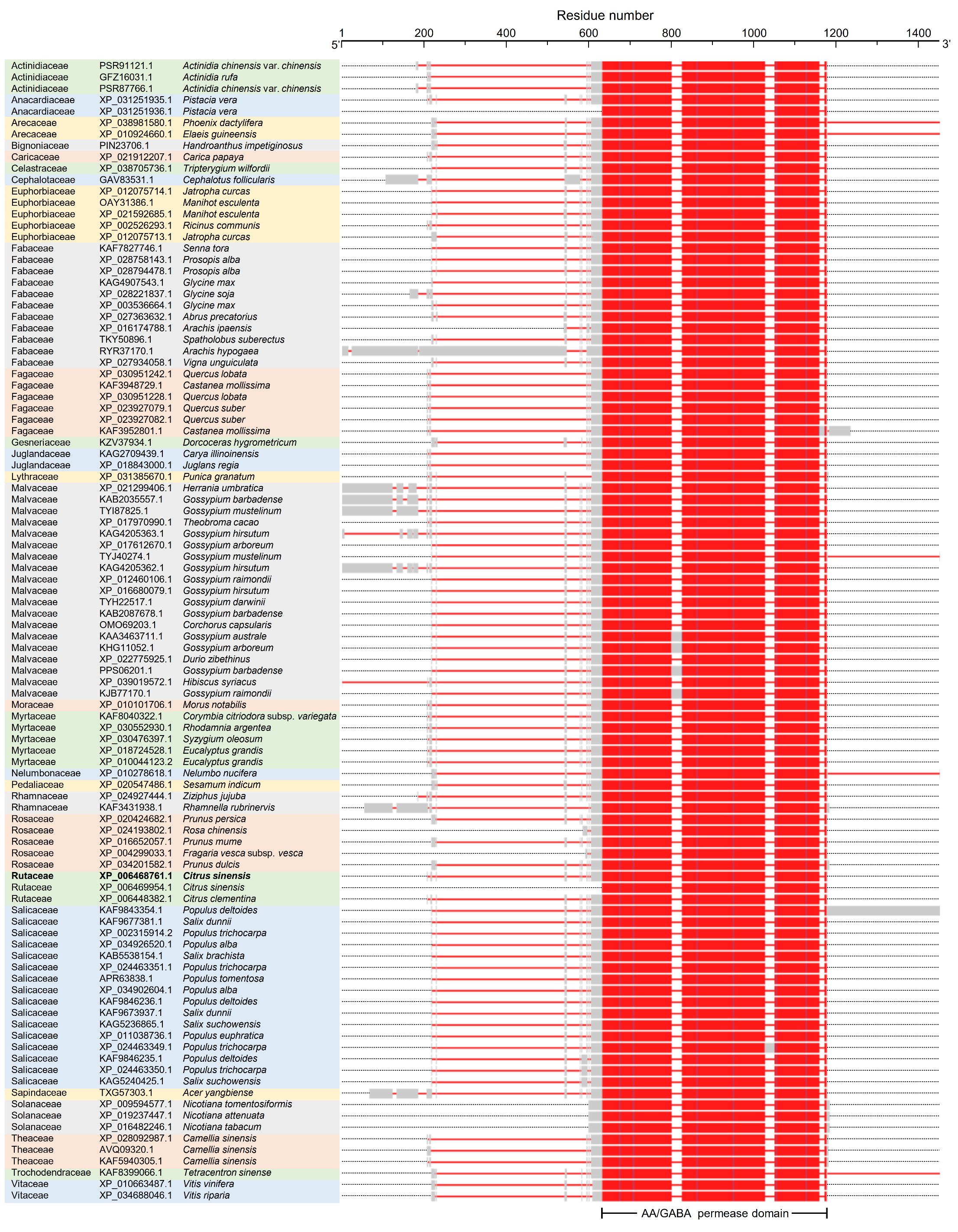


## Figure S1: Multiple sequence alignment of GABA permease (*gabP,* aka amino-acid permease [BAT1]) proteins from different plant species using the Constraint-based Multiple Alignment Tool (COBALT). Residues were colored using a column-based method according to their relative entropy threshold. Aligned columns with no gaps are colored blue and red, where the red color indicates highly conserved columns and blue indicates less conserved ones. The listed genes were identified based on recently available data in GenBank, National Center for Biotechnology Information website (NCBI, <https://www.ncbi.nlm.nih.gov/protein/>). The full list of genes, names, and accession numbers is available in supplementary Table S5.


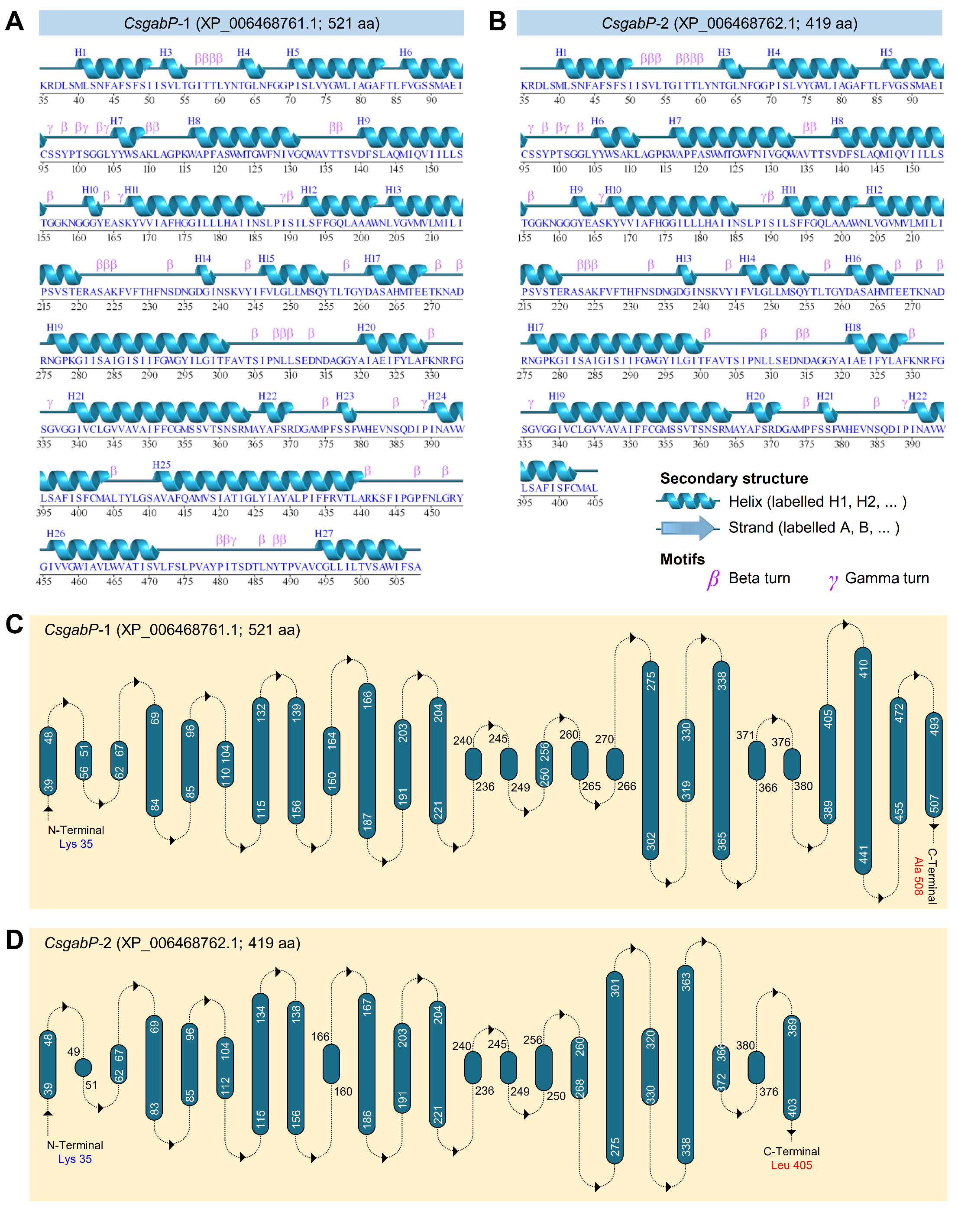


## Figure S2. The secondary structure of putative GABA permeases (*CsgabP*s*,* aka amino-acid permease [BAT1]) from *Citrus sinensis*. (A and B) The predicted secondary structures of *CsgabP-*1 (XP_006468761.1) and *CsgabP-*2 (XP_006468762.1), respectively. The secondary structures were generated using the PDBsum tool. (C and D) The secondary structure-associated topology diagrams of *CsgabP-*1 and *CsgabP-*2, respectively, show the arrangement and connectivity of the helices and strands in protein. *α*-helices of the predicted secondary structures are shown as rounded rectangles, whereas *β*-sheets are represented by arrows.


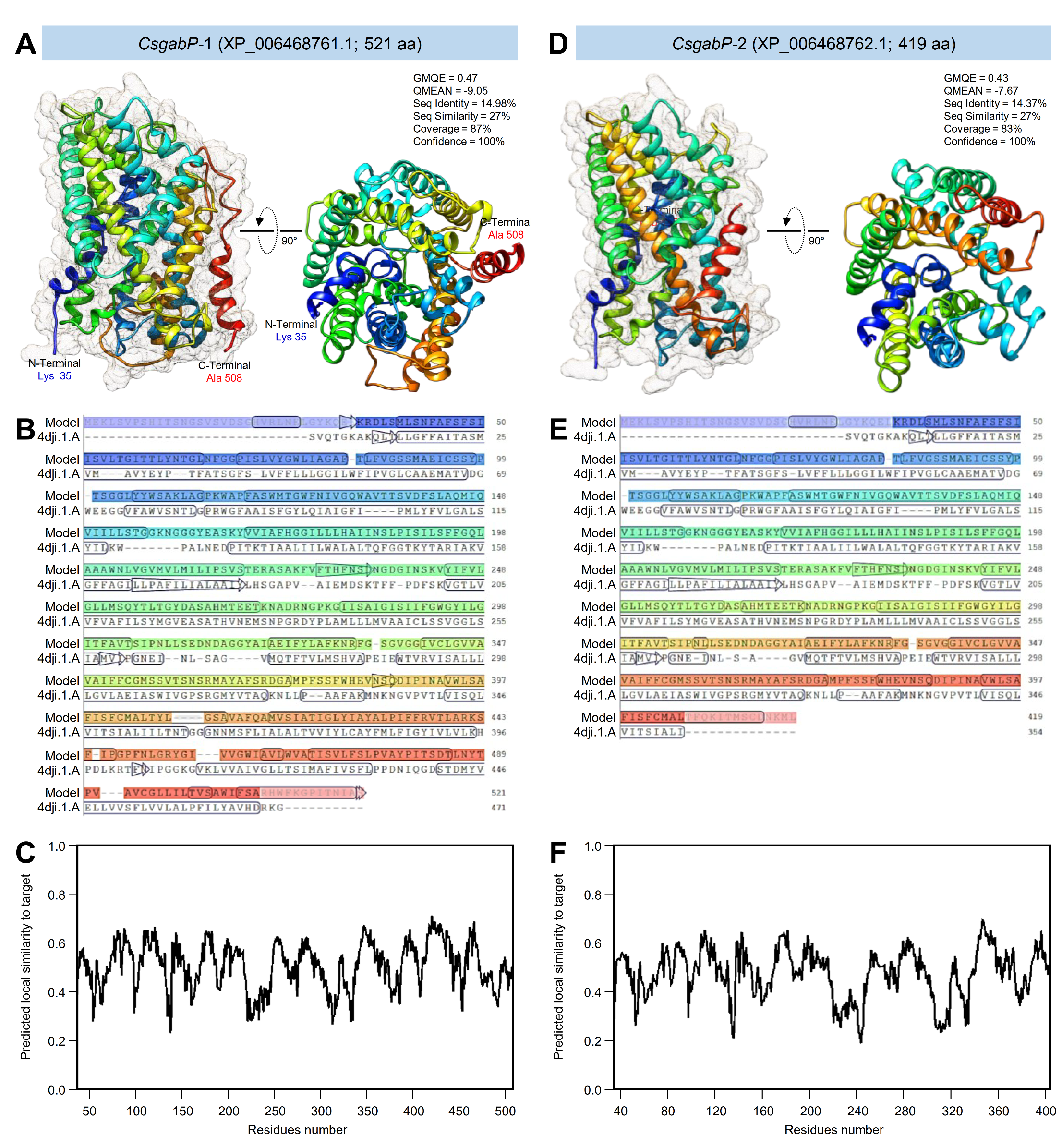


## Figure S3. The crystallographic tertiary structure of putative GABA permeases (*CsgabP,* aka amino-acid permease [BAT1]) from *Citrus sinensis*. (A and D) SWISS-MODEL-based predicted three-dimensional (3D) structures and their associated mesh surface of *CsgabP-*1 (XP_006468761.1) and *CsgabP-*2 (XP_006468762.1), respectively. The tertiary structures of *CsgabPs* were predicted using the crystal structure of probable glutamate/gamma-aminobutyrate antiporter (aka glutamate-GABA antiporter [GadC] from *Escherichia coli* (strain K12) in the protein data bank (PDB ID: [4dji.1.A](https://swissmodel.expasy.org/templates/4dji.1)) and refined using the X-ray method to 3.19 Å resolution with 100.0% confidence. Protein chains are colored according to the rainbow color spectrum, from blue (N-terminus) to red (C-terminus). The tertiary structures were generated using the SWISS-MODEL server and visualized with the UCSF-Chimera package. (B and E) Model–template alignment of *CsgabP-*1 and *CsgabP-*2. AA sequences of each model were aligned with the template ([4dji.1.A](https://swissmodel.expasy.org/templates/4dji.1)). *α*-Helices of the predicted secondary structures are shown as rectangles, whereas *β*-sheets are represented by arrows. Matched sequences are black-colored. (C and F) Local quality estimate of the predicted models of *CsgabP-*1 and *CsgabP-*2, respectively, to target. GMQE: Global model quality estimation.


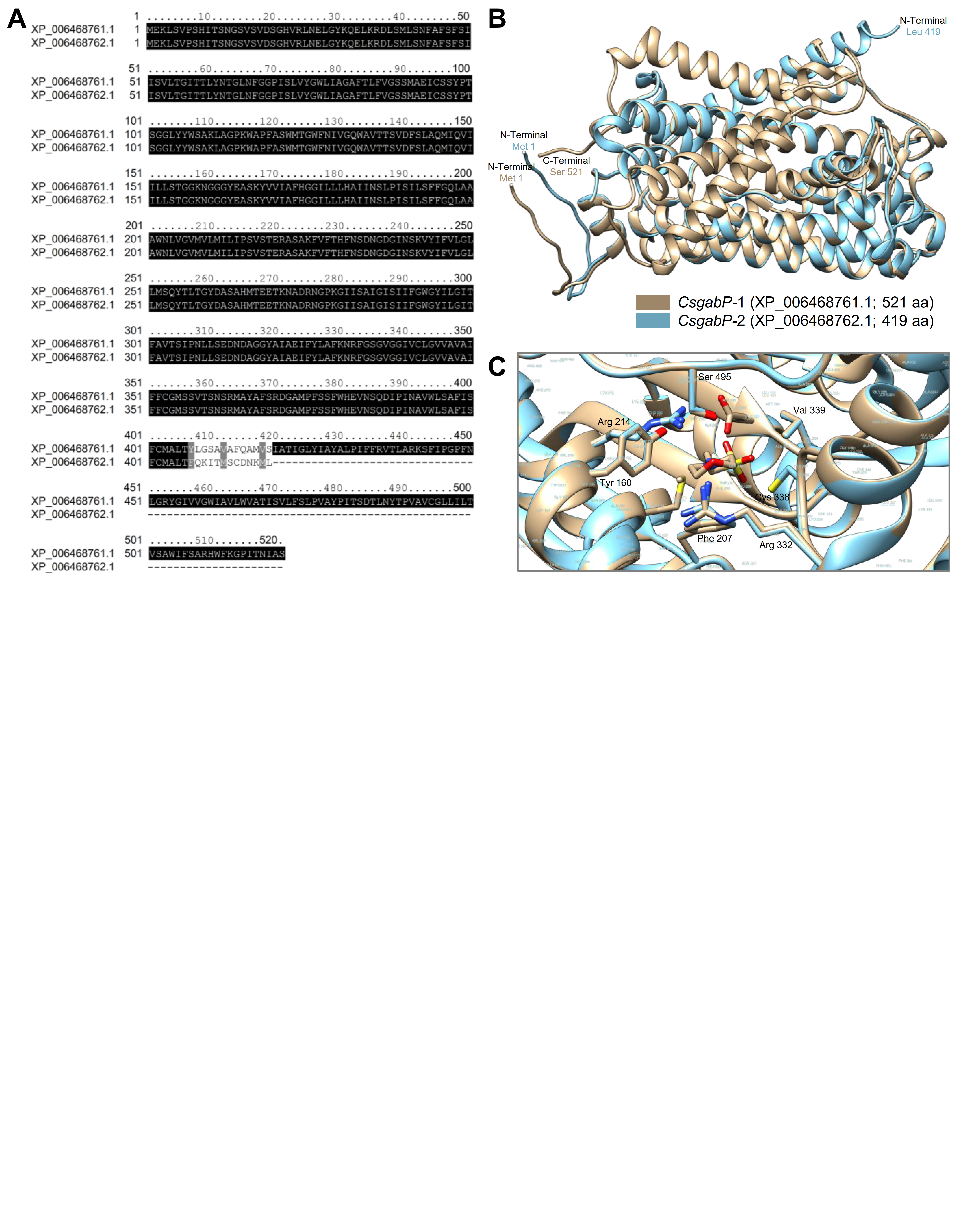


## Figure S4: Multiple sequence alignment of the predicted crystallographic tertiary structure (3D) of putative GABA permeases (*CsgabP,* aka amino-acid permease [BAT1]) from *Citrus sinensis*. (A) CLUSTALW-based multiple sequence alignment of *CsgabP-*1 (XP_006468761.1; 521 aa) and *CsgabP-*2 (XP_006468762.1; 419 aa) from *C. sinensis*. Conserved amino acids are indicated with black shading and those with high similarity scores are in gray. Numbers denote amino acid residue numbers for the start and end, respectively. The full list of genes, names, and accession numbers are available in supplementary Table S2. (B) 3D structure alignment of *CsgabP-*1 (XP_006468761.1; 521 aa) colored in golden and *CsgabP-*2 (XP_006468762.1; 419 aa) colored in baby-blue from *C. sinensis*. 3D molecular structures were analyzed using structure comparison, with the MatchMaker tool that superimposed structures based on sequence alignments and interactively visualized using UCSF Chimera. and (C) Binding pockets of *CsgabP-*1 (colored in golden) and *CsgabP-*2 (colored in baby-blue) from *C. sinensis*.

# Supplementary Tables

## Table S1. Primer used for gene expression analysis of putative *gab* genes Valencia sweet orange (*C. sinensis*) leaves using real-time RT-PCR ^a^.

| **Gene** | **Accession ID** |  | **Primer**  **(Forward and Reverse)** | **TM**  **(˚C)** | **Product size**  **(bp)** |
| --- | --- | --- | --- | --- | --- |
| ***CsgabP*** | XM_006468698.3 | F: | TTCATCCCCGGACCTTTCAA | 58.93 | 197 |
|  |  | R: | CGAGCACTGAAGATCCAAGC | 58.99 |  |
| ***CsEF1* ^b^** | AY498567.1 | F: | GGAAGTTCGAGACCACCAAG | 59.70 | 202 |
|  |  | R: | ACACCAAGGGTGAAAGCAAG | 60.15 |  |
| ***CsF-box* ^b^** | XM_006482390.1 | F: | ACTTGACAGATGGGCTGTCC | 60.12 | 197 |
|  |  | R: | CAGCAACCAAATACCCGTCT | 59.99 |  |
| ***CsGAPC1* ^b^** | XM_006483974.2 | F: | ACTCCAGAGGGATGATGTGG | 59.92 | 200 |
|  |  | R: | ATGGGATCTCCTCTGGGTTC | 60.28 |  |
| ***CsSAND* ^b^** | XM_006488024.2 | F: | GCATCAGCTGCACAGAAGAG | 59.89 | 204 |
|  |  | R: | GGAATGTAGCTGGGTTCCAA | 59.93 |  |

**^a^** The listed genes were identified based on recently available data in GenBank, National Center for Biotechnology Information website (NCBI, <http://www.ncbi.nlm.nih.gov/gene/>).

**^b^** Genes have been used as reference/housekeeping genes for data normalization according to ^36,37^.

***Abbreviations***

- ***CsgabP:*** *C.sinensis* GABA permease (aka PREDICTED: amino-acid permease BAT1-like isoform X1).
- ***CsEF-1α***: *C.sinensis* elongation factor-1 alpha.
- ***CsF-box****:* *C. sinensis* F-box/kelch-repeat protein.
- ***CsGAPC1****: C. sinensis* glyceraldehyde-3-phosphate dehydrogenase, cytosolic (aka GAPDH).
- ***CsSAND***: *C. sinensis* SAND family protein.

## Table S2. Sequences from *Citrus sinensis* that producing significant alignments with GABA permease (*gabP*) from *Arabidopsis thaliana* using NCBI database ^a^.

| **Description** | **NCBI**  **Accession number** | [**Accession Len**](https://blast.ncbi.nlm.nih.gov/Blast.cgi)**gth**  **(aa)** | **Max Score** | **Total Score** | **Query Cover**  **(%)** | **Identity**  **(%)** | **E value** |
| --- | --- | --- | --- | --- | --- | --- | --- |
| Amino-acid permease BAT1-like isoform X1 | [XP_006468761.1](https://www.ncbi.nlm.nih.gov/protein/XP_006468761.1?report=genbank&log$=prottop&blast_rank=1&RID=E45EUUEK013) | 521 | 823 | 823 | 97 | 80.95 | 0.0 |
| Amino-acid permease BAT1-like isoform X2 | [XP_006468762.1](https://www.ncbi.nlm.nih.gov/protein/XP_006468762.1?report=genbank&log$=prottop&blast_rank=3&RID=E45EUUEK013) | 419 | 642 | 642 | 75 | 81.75 | 0.0 |
| Amino-acid permease BAT1 ^b^ | [XP_006469954.1](https://www.ncbi.nlm.nih.gov/protein/XP_006469954.1?report=genbank&log$=prottop&blast_rank=2&RID=E45EUUEK013) | 482 | 781 | 781 | 93 | 80.71 | 0.0 |
| Amino-acid permease BAT1-like | [XP_024952885.1](https://www.ncbi.nlm.nih.gov/protein/XP_024952885.1?report=genbank&log$=prottop&blast_rank=4&RID=E45EUUEK013) | 542 | 418 | 418 | 97 | 42.38 | 3e-141 |
| Hypothetical protein CISIN_1g010352mg | [KDO57518.1](https://www.ncbi.nlm.nih.gov/protein/KDO57518.1?report=genbank&log$=prottop&blast_rank=5&RID=E45EUUEK013) | 512 | 404 | 404 | 97 | 42.18 | 5e-136 |
| Hypothetical protein CISIN_1g010352mg | [KDO57519.1](https://www.ncbi.nlm.nih.gov/protein/KDO57519.1?report=genbank&log$=prottop&blast_rank=6&RID=E45EUUEK013) | 478 | 345 | 345 | 88 | 40.83 | 1e-113 |
| Hypothetical protein CISIN_1g010352mg | [KDO57520.1](https://www.ncbi.nlm.nih.gov/protein/KDO57520.1?report=genbank&log$=prottop&blast_rank=7&RID=E45EUUEK013) | 394 | 272 | 272 | 72 | 39.84 | 3e-86 |
| [Hypothetical protein CISIN_1g010352mg](https://blast.ncbi.nlm.nih.gov/Blast.cgi#alnHdr_KDO57522) | [KDO57522.1](https://www.ncbi.nlm.nih.gov/protein/KDO57522.1?report=genbank&log$=prottop&blast_rank=8&RID=E45EUUEK013) | 317 | 230 | 230 | 59 | 41.29 | 3e-71 |

^a^ The listed genes were identified using the protein-protein BLAST (BLASTp) using bidirectional amino acid transporter 1 (BAT1; GenBank accession no. [NP_565254.1](https://www.ncbi.nlm.nih.gov/protein/NP_565254.1); 516 aa) from *Arabidopsis thaliana* (aka GABA permease [*AtgabP* ]) ^2^ as a query sequence against *Citrus sinensis* genome available in GenBank, National Center for Biotechnology Information website (NCBI, <http://www.ncbi.nlm.nih.gov/gene/>), using the compositionally adjusted substitution matrices ^3^.

^b^ This record was removed as a result of standard genome annotation processing

## Table S3. Alignment statistics for the top matched putative *CsgabP* proteins from *Citrus sinensis*.

| **NCBI Database** | | | | | | | | | | | | | ***Citrus sinensis* Genome Database** | | | | | |
| --- | --- | --- | --- | --- | --- | --- | --- | --- | --- | --- | --- | --- | --- | --- | --- | --- | --- | --- |
| **Gene Description** | **Gene ID** | **mRNA** | | **Protein** | | | | **Protein-Protein Alignment statistics** | | | | | **Gene Description** | **Protein** | | **Protein-Protein Alignment statistics** | | |
|  |  | Accession | bp | Accession | aa |  |  | Max Score | Total Score | Query Cover  (%) | Identity  (%) | E value |  | Accession | aa | Identities  (%) | Positives  (%) | E value |
| Amino-acid permease BAT1-like isoform X1 (*CsgabP*-1) | LOC102610833 | [XM_006468698.3](https://www.ncbi.nlm.nih.gov/nuccore/XM_006468698.3) | 1927 | [XP_006468761.1](https://www.ncbi.nlm.nih.gov/protein/XP_006468761.1?report=genbank&log$=prottop&blast_rank=1&RID=5H9930W5016) | 521 |  |  | 823 | 823 | 97 | 80.95 | 0.0 | Bidirectional amino acid transporter 1 | [orange1.1g011598m](https://www.citrusgreening.org/tools/blast/match/show?blast_db_id=7;id=orange1.1g011598m;hilite_coords=1-482) | 482 | 100 | 100 | 0.0 |
| Amino-acid permease BAT1-like isoform X2 (*CsgabP*-2) | LOC102610833 | [XM_006468699.3](https://www.ncbi.nlm.nih.gov/nuccore/XM_006468699.3) | 1643 | [XP_006468762.1](https://www.ncbi.nlm.nih.gov/protein/XP_006468762.1?report=genbank&log$=prottop&blast_rank=3&RID=5H9930W5016) | 419 |  |  | 781 | 781 | 93 | 80.71 | 0.0 | Bidirectional amino acid transporter 1 | [orange1.1g011598m](https://www.citrusgreening.org/tools/blast/match/show?blast_db_id=7;id=orange1.1g011598m;hilite_coords=1-368) | 482 | 99.73 | 100 | 0.0 |

The listed putative gene candidates were identified using the protein-protein BLAST (BLASTp) using bidirectional amino acid transporter 1 (BAT1; GenBank accession no. [NP_565254.1](https://www.ncbi.nlm.nih.gov/protein/NP_565254.1); 516 aa) from *Arabidopsis thaliana* (aka GABA permease [*AtgabP* ]) ^2^ as a query sequence against *Citrus sinensis* genome available in GenBank, National Center for Biotechnology Information website (NCBI, <http://www.ncbi.nlm.nih.gov/gene/>), and the "*Citrus sinensis* CDS, phytozome 154 v1.1" and "*Citrus sinensis proteins*, phytozome 154 v1.1" datasets available on Citrus Greening Solutions website (<https://citrusgreening.org/organism/Citrus_sinensis/genome>) ^1^, using the compositionally adjusted substitution matrices ^3,4^. The shortlist of top matches was generated based on the phylogenetic trees, identity more than 90%, and excluding all the hypothetical and low-quality proteins that have these characteristics, as well as obsolete records that were removed as a result of standard genome annotation processing.

## Table S4. Physicochemical properties of putative *CsgabP* proteins from *C. sinensis* compared with *AtgabP* from *A. thaliana*

| Protein | Theoretical isoelectric point (pI) | Molecular weight  (MW; kDa) | Extinction coefficients (ε) ^a^ | | | | Instability index (II) | Aliphatic index | GRAVY ^d^ | −R ^e^ | +R ^f^ |
| --- | --- | --- | --- | --- | --- | --- | --- | --- | --- | --- | --- |
|  |  |  | ε-I | Abs-I ^b^ | ε-II | Abs-II ^c^ |  |  |  |  |  |
| *AtgabP* | 8.17 | 55.33 | 110030 | 1.989 | 109780 | 1.984 | 30.56 | 111.28 | 0.726 | 21 | 23 |
| *CsgabP*-1 | 8.16 | 56.07 | 108540 | 1.936 | 108290 | 1.931 | 26.27 | 106.33 | 0.648 | 23 | 25 |
| *CsgabP*-2 | 6.37 | 45.13 | 77600 | 1.720 | 77350 | 1.714 | 30.98 | 99.16 | 0.511 | 23 | 21 |

^a^ Extinction coefficients (ε) are in units of M^-1^ cm^-1^, at 280 nm measured in water.

^b^ Abs-I 0.1%: (=1 g.L^−1^) assuming all pairs of Cys residues form cystines.

^c^ Abs-II 0.1%: (=1 g.L^−1^) assuming all Cys residues are reduced.

^d^ GRAVY: Grand average of hydropathicity

^e^ −R: Total number of negatively charged residues (Asp + Glu)

^f^ +R: Total number of positively charged residues (Arg + Lys)

## Table S5. Sequences of GABA permease (*gabP*) from different plant species retrieved in this study ^a^.

| **Description** | **Accession No.** | [**Len**](https://blast.ncbi.nlm.nih.gov/Blast.cgi)**gth**  **(aa)** | **Species** | **Family** | **Max Score** | **Total Score** | **Query Cover**  **(%)** | **Identity**  **(%)** | **E value** |
| --- | --- | --- | --- | --- | --- | --- | --- | --- | --- |
| Amino-acid permease BAT1-like isoform X1 | [XP_006468761.1](https://www.ncbi.nlm.nih.gov/protein/XP_006468761.1?report=genbank&log$=prottop&blast_rank=1&RID=78JKE0EJ016) | 521 | *Citrus sinensis* | Rutaceae | 1041 | 1041 | 100 | 100.00 | 0.0 |
| Amino-acid permease BAT1 | [XP_006448382.1](https://www.ncbi.nlm.nih.gov/protein/XP_006448382.1?report=genbank&log$=prottop&blast_rank=2&RID=78JKE0EJ016) | 521 | *Citrus clementina* | Rutaceae | 1039 | 1039 | 100 | 99.81 | 0.0 |
| Amino-acid permease BAT1 | [XP_006469954.1](https://www.ncbi.nlm.nih.gov/protein/XP_006469954.1?report=genbank&log$=prottop&blast_rank=3&RID=78JKE0EJ016) | 482 | *Citrus sinensis* | Rutaceae | 959 | 959 | 92 | 100.00 | 0.0 |
| Hypothetical protein EZV62_018616 | [TXG57303.1](https://www.ncbi.nlm.nih.gov/protein/TXG57303.1?report=genbank&log$=prottop&blast_rank=4&RID=78JKE0EJ016) | 635 | *Acer yangbiense* | Sapindaceae | 943 | 1050 | 99 | 90.00 | 0.0 |
| Amino-acid permease BAT1 homolog isoform X1 | [XP_031251935.1](https://www.ncbi.nlm.nih.gov/protein/XP_031251935.1?report=genbank&log$=prottop&blast_rank=5&RID=78JKE0EJ016) | 525 | *Pistacia vera* | Anacardiaceae | 932 | 932 | 99 | 89.81 | 0.0 |
| Amino-acid permease BAT1 homolog isoform X2 | [XP_012075714.1](https://www.ncbi.nlm.nih.gov/protein/XP_012075714.1?report=genbank&log$=prottop&blast_rank=6&RID=78JKE0EJ016) | 518 | *Jatropha curcas* | Euphorbiaceae | 927 | 927 | 99 | 87.07 | 0.0 |
| Amino-acid permease BAT1 isoform X3 | [XP_021912207.1](https://www.ncbi.nlm.nih.gov/protein/XP_021912207.1?report=genbank&log$=prottop&blast_rank=7&RID=78JKE0EJ016) | 525 | *Carica papaya* | Caricaceae | 927 | 927 | 99 | 89.38 | 0.0 |
| Hypothetical protein H0E87_012052 | [KAF9843354.1](https://www.ncbi.nlm.nih.gov/protein/KAF9843354.1?report=genbank&log$=prottop&blast_rank=8&RID=78JKE0EJ016) | 793 | *Populus deltoides* | Salicaceae | 924 | 924 | 99 | 86.29 | 0.0 |
| Hypothetical protein SADUNF_Sadunf08G0101700 | [KAF9677381.1](https://www.ncbi.nlm.nih.gov/protein/KAF9677381.1?report=genbank&log$=prottop&blast_rank=9&RID=78JKE0EJ016) | 518 | *Salix dunnii* | Salicaceae | 918 | 918 | 99 | 86.49 | 0.0 |
| Amino-acid permease BAT1 homolog | [XP_002315914.2](https://www.ncbi.nlm.nih.gov/protein/XP_002315914.2?report=genbank&log$=prottop&blast_rank=10&RID=78JKE0EJ016) | 518 | *Populus trichocarpa* | Salicaceae | 917 | 917 | 99 | 86.29 | 0.0 |
| Amino-acid permease BAT1 isoform X2 | [XP_021299406.1](https://www.ncbi.nlm.nih.gov/protein/XP_021299406.1?report=genbank&log$=prottop&blast_rank=11&RID=78JKE0EJ016) | 682 | *Herrania umbratica* | Malvaceae | 917 | 1070 | 99 | 88.42 | 0.0 |
| Amino-acid permease BAT1-like | [XP_034926520.1](https://www.ncbi.nlm.nih.gov/protein/XP_034926520.1?report=genbank&log$=prottop&blast_rank=12&RID=78JKE0EJ016) | 518 | *Populus alba* | Salicaceae | 917 | 917 | 99 | 86.29 | 0.0 |
| Hypothetical protein DKX38_015687 | [KAB5538154.1](https://www.ncbi.nlm.nih.gov/protein/KAB5538154.1?report=genbank&log$=prottop&blast_rank=13&RID=78JKE0EJ016) | 518 | *Salix brachista* | Salicaceae | 916 | 916 | 99 | 86.10 | 0.0 |
| Amino-acid permease BAT1 homolog isoform X4 | [XP_024463351.1](https://www.ncbi.nlm.nih.gov/protein/XP_024463351.1?report=genbank&log$=prottop&blast_rank=14&RID=78JKE0EJ016) | 516 | *Populus trichocarpa* | Salicaceae | 915 | 915 | 99 | 86.43 | 0.0 |
| Hypothetical protein | [APR63838.1](https://www.ncbi.nlm.nih.gov/protein/APR63838.1?report=genbank&log$=prottop&blast_rank=15&RID=78JKE0EJ016) | 516 | *Populus tomentosa* | Salicaceae | 915 | 915 | 99 | 86.82 | 0.0 |
| Amino-acid permease BAT1 homolog isoform X2 | [XP_034902604.1](https://www.ncbi.nlm.nih.gov/protein/XP_034902604.1?report=genbank&log$=prottop&blast_rank=16&RID=78JKE0EJ016) | 518 | *Populus alba* | Salicaceae | 914 | 914 | 99 | 86.49 | 0.0 |
| Hypothetical protein H0E87_021045 | [KAF9846236.1](https://www.ncbi.nlm.nih.gov/protein/KAF9846236.1?report=genbank&log$=prottop&blast_rank=17&RID=78JKE0EJ016) | 518 | *Populus deltoides* | Salicaceae | 913 | 913 | 99 | 86.10 | 0.0 |
| Hypothetical protein SADUNF_Sadunf10G0075700 | [KAF9673937.1](https://www.ncbi.nlm.nih.gov/protein/KAF9673937.1?report=genbank&log$=prottop&blast_rank=18&RID=78JKE0EJ016) | 518 | *Salix dunnii* | Salicaceae | 913 | 913 | 99 | 85.91 | 0.0 |
| Amino-acid permease BAT | [KAG5236865.1](https://www.ncbi.nlm.nih.gov/protein/KAG5236865.1?report=genbank&log$=prottop&blast_rank=19&RID=78JKE0EJ016) | 518 | *Salix suchowensis* | Salicaceae | 911 | 911 | 99 | 85.52 | 0.0 |
| Hypothetical protein MANES_14G108000 | [OAY31386.1](https://www.ncbi.nlm.nih.gov/protein/OAY31386.1?report=genbank&log$=prottop&blast_rank=20&RID=78JKE0EJ016) | 518 | *Manihot esculenta* | Euphorbiaceae | 910 | 910 | 99 | 85.71 | 0.0 |
| Hypothetical protein ES319_D04G160400v1 | [KAB2035557.1](https://www.ncbi.nlm.nih.gov/protein/KAB2035557.1?report=genbank&log$=prottop&blast_rank=21&RID=78JKE0EJ016) | 685 | *Gossypium barbadense* | Malvaceae | 909 | 1061 | 99 | 88.03 | 0.0 |
| Hypothetical protein E1A91_D04G163000v1 | [TYI87825.1](https://www.ncbi.nlm.nih.gov/protein/TYI87825.1?report=genbank&log$=prottop&blast_rank=22&RID=78JKE0EJ016) | 697 | *Gossypium mustelinum* | Malvaceae | 908 | 1064 | 99 | 88.03 | 0.0 |
| Amino-acid permease BAT1 isoform X2 | [XP_017970990.1](https://www.ncbi.nlm.nih.gov/protein/XP_017970990.1?report=genbank&log$=prottop&blast_rank=23&RID=78JKE0EJ016) | 525 | *Theobroma cacao* | Malvaceae | 908 | 908 | 99 | 87.64 | 0.0 |
| Hypothetical protein ERO13_A04G099600v2 | [KAG4205363.1](https://www.ncbi.nlm.nih.gov/protein/KAG4205363.1?report=genbank&log$=prottop&blast_rank=24&RID=78JKE0EJ016) | 560 | *Gossypium hirsutum* | Malvaceae | 908 | 908 | 99 | 87.64 | 0.0 |
| Amino-acid permease BAT1 homolog | [XP_017612670.1](https://www.ncbi.nlm.nih.gov/protein/XP_017612670.1?report=genbank&log$=prottop&blast_rank=25&RID=78JKE0EJ016) | 516 | *Gossypium arboreum* | Malvaceae | 906 | 906 | 99 | 87.98 | 0.0 |
| Hypothetical protein E1A91_A04G128500v1 | [TYJ40274.1](https://www.ncbi.nlm.nih.gov/protein/TYJ40274.1?report=genbank&log$=prottop&blast_rank=26&RID=78JKE0EJ016) | 521 | *Gossypium mustelinum* | Malvaceae | 906 | 906 | 99 | 87.64 | 0.0 |
| Amino-acid permease BAT1 homolog | [XP_011038736.1](https://www.ncbi.nlm.nih.gov/protein/XP_011038736.1?report=genbank&log$=prottop&blast_rank=27&RID=78JKE0EJ016) | 517 | *Populus euphratica* | Salicaceae | 906 | 906 | 99 | 85.47 | 0.0 |
| Hypothetical protein ERO13_A04G099600v2 | [KAG4205362.1](https://www.ncbi.nlm.nih.gov/protein/KAG4205362.1?report=genbank&log$=prottop&blast_rank=28&RID=78JKE0EJ016) | 683 | *Gossypium hirsutum* | Malvaceae | 906 | 1057 | 99 | 87.64 | 0.0 |
| Amino-acid permease BAT1 homolog isoform X2 | [XP_028092987.1](https://www.ncbi.nlm.nih.gov/protein/XP_028092987.1?report=genbank&log$=prottop&blast_rank=29&RID=78JKE0EJ016) | 517 | *Camellia sinensis* | Theaceae | 905 | 905 | 98 | 85.94 | 0.0 |
| Amino-acid permease BAT1 homolog isoform X2 | [XP_012460106.1](https://www.ncbi.nlm.nih.gov/protein/XP_012460106.1?report=genbank&log$=prottop&blast_rank=30&RID=78JKE0EJ016) | 516 | *Gossypium raimondii* | Malvaceae | 905 | 905 | 99 | 88.18 | 0.0 |
| Bidirectional amino acid transporter | [AVQ09320.1](https://www.ncbi.nlm.nih.gov/protein/AVQ09320.1?report=genbank&log$=prottop&blast_rank=31&RID=78JKE0EJ016) | 521 | *Camellia sinensis* | Theaceae | 904 | 904 | 98 | 85.94 | 0.0 |
| Amino-acid permease BAT1 homolog isoform X2 | [XP_016680079.1](https://www.ncbi.nlm.nih.gov/protein/XP_016680079.1?report=genbank&log$=prottop&blast_rank=32&RID=78JKE0EJ016) | 516 | *Gossypium hirsutum* | Malvaceae | 904 | 904 | 99 | 87.98 | 0.0 |
| Hypothetical protein ES288_A04G134800v1 | [TYH22517.1](https://www.ncbi.nlm.nih.gov/protein/TYH22517.1?report=genbank&log$=prottop&blast_rank=33&RID=78JKE0EJ016) | 516 | *Gossypium darwinii* | Malvaceae | 904 | 904 | 99 | 87.79 | 0.0 |
| Hypothetical protein HYC85_021472 | [KAF5940305.1](https://www.ncbi.nlm.nih.gov/protein/KAF5940305.1?report=genbank&log$=prottop&blast_rank=34&RID=78JKE0EJ016) | 517 | *Camellia sinensis* | Theaceae | 903 | 903 | 98 | 85.94 | 0.0 |
| Hypothetical protein ES319_A04G120700v1 | [KAB2087678.1](https://www.ncbi.nlm.nih.gov/protein/KAB2087678.1?report=genbank&log$=prottop&blast_rank=35&RID=78JKE0EJ016) | 516 | *Gossypium barbadense* | Malvaceae | 902 | 902 | 99 | 87.60 | 0.0 |
| Amino-acid permease BAT1 homolog isoform X2 | [XP_024463349.1](https://www.ncbi.nlm.nih.gov/protein/XP_024463349.1?report=genbank&log$=prottop&blast_rank=36&RID=78JKE0EJ016) | 540 | *Populus trichocarpa* | Salicaceae | 902 | 902 | 99 | 82.59 | 0.0 |
| Amino-acid permease BAT1 isoform X2 | [XP_021592685.1](https://www.ncbi.nlm.nih.gov/protein/XP_021592685.1?report=genbank&log$=prottop&blast_rank=37&RID=78JKE0EJ016) | 523 | *Manihot esculenta* | Euphorbiaceae | 900 | 900 | 97 | 86.27 | 0.0 |
| Amino-acid permease BAT1 homolog isoform X1 | [XP_020547486.1](https://www.ncbi.nlm.nih.gov/protein/XP_020547486.1?report=genbank&log$=prottop&blast_rank=38&RID=78JKE0EJ016) | 528 | *Sesamum indicum* | Pedaliaceae | 898 | 898 | 99 | 86.54 | 0.0 |
| Hypothetical protein BT93_B2531 | [KAF8040322.1](https://www.ncbi.nlm.nih.gov/protein/KAF8040322.1?report=genbank&log$=prottop&blast_rank=39&RID=78JKE0EJ016) | 525 | *Corymbia citriodora* subsp*. variegata* | Myrtaceae | 898 | 898 | 99 | 85.91 | 0.0 |
| Amino-acid permease BAT1 homolog isoform X2 | [XP_030552930.1](https://www.ncbi.nlm.nih.gov/protein/XP_030552930.1?report=genbank&log$=prottop&blast_rank=40&RID=78JKE0EJ016) | 525 | *Rhodamnia argentea* | Myrtaceae | 897 | 897 | 99 | 85.52 | 0.0 |
| Amino-acid permease BAT1 homolog isoform X2 | [XP_030476397.1](https://www.ncbi.nlm.nih.gov/protein/XP_030476397.1?report=genbank&log$=prottop&blast_rank=41&RID=78JKE0EJ016) | 525 | *Syzygium oleosum* | Myrtaceae | 896 | 896 | 99 | 85.71 | 0.0 |
| Amino-acid permease BAT1 homolog isoform X1 | [XP_024927444.1](https://www.ncbi.nlm.nih.gov/protein/XP_024927444.1?report=genbank&log$=prottop&blast_rank=42&RID=78JKE0EJ016) | 527 | *Ziziphus jujuba* | Rhamnaceae | 896 | 896 | 99 | 86.10 | 0.0 |
| Amino-acid permease BAT1-like isoform X1 | [XP_038705736.1](https://www.ncbi.nlm.nih.gov/protein/XP_038705736.1?report=genbank&log$=prottop&blast_rank=43&RID=78JKE0EJ016) | 518 | *Tripterygium wilfordii* | Celastraceae | 896 | 896 | 99 | 86.24 | 0.0 |
| Amino acid/polyamine transporter I | [OMO69203.1](https://www.ncbi.nlm.nih.gov/protein/OMO69203.1?report=genbank&log$=prottop&blast_rank=44&RID=78JKE0EJ016) | 519 | *Corchorus capsularis* | Malvaceae | 894 | 894 | 99 | 86.07 | 0.0 |
| Amino-acid permease BAT1-like protein | [KAA3463711.1](https://www.ncbi.nlm.nih.gov/protein/KAA3463711.1?report=genbank&log$=prottop&blast_rank=45&RID=78JKE0EJ016) | 542 | *Gossypium australe* | Malvaceae | 894 | 894 | 99 | 83.95 | 0.0 |
| Amino acid transporter | [PIN23706.1](https://www.ncbi.nlm.nih.gov/protein/PIN23706.1?report=genbank&log$=prottop&blast_rank=46&RID=78JKE0EJ016) | 528 | *Handroanthus impetiginosus* | Bignoniaceae | 893 | 893 | 99 | 85.66 | 0.0 |
| amino-acid permease C15C4.04c | [KHG11052.1](https://www.ncbi.nlm.nih.gov/protein/KHG11052.1?report=genbank&log$=prottop&blast_rank=47&RID=78JKE0EJ016) | 542 | *Gossypium arboreum* | Malvaceae | 892 | 892 | 99 | 83.76 | 0.0 |
| Amino-acid permease | [PSR91121.1](https://www.ncbi.nlm.nih.gov/protein/PSR91121.1?report=genbank&log$=prottop&blast_rank=48&RID=78JKE0EJ016) | 522 | *Actinidia chinensis* var*. chinensis* | Actinidiaceae | 892 | 892 | 99 | 85.96 | 0.0 |
| Hypothetical protein I3760_05G239500 | [KAG2709439.1](https://www.ncbi.nlm.nih.gov/protein/KAG2709439.1?report=genbank&log$=prottop&blast_rank=49&RID=78JKE0EJ016) | 515 | *Carya illinoinensis* | Juglandaceae | 892 | 892 | 97 | 87.45 | 0.0 |
| Amino-acid permease BAT1 isoform X2 | [XP_002526293.1](https://www.ncbi.nlm.nih.gov/protein/XP_002526293.1?report=genbank&log$=prottop&blast_rank=50&RID=78JKE0EJ016) | 528 | *Ricinus communis* | Euphorbiaceae | 890 | 890 | 99 | 85.82 | 0.0 |
| Amino-acid permease BAT1 isoform X2 | [XP_022775925.1](https://www.ncbi.nlm.nih.gov/protein/XP_022775925.1?report=genbank&log$=prottop&blast_rank=51&RID=78JKE0EJ016) | 518 | *Durio zibethinus* | Malvaceae | 890 | 890 | 99 | 87.40 | 0.0 |
| Bidirectional amino acid transporter 1 | [GFZ16031.1](https://www.ncbi.nlm.nih.gov/protein/GFZ16031.1?report=genbank&log$=prottop&blast_rank=52&RID=78JKE0EJ016) | 517 | *Actinidia rufa* | Actinidiaceae | 888 | 888 | 99 | 86.10 | 0.0 |
| Amino-acid permease BAT1 homolog isoform X1 | [XP_030951242.1](https://www.ncbi.nlm.nih.gov/protein/XP_030951242.1?report=genbank&log$=prottop&blast_rank=53&RID=78JKE0EJ016) | 515 | *Quercus lobata* | Fagaceae | 888 | 888 | 97 | 86.61 | 0.0 |
| Hypothetical protein GOBAR_AA14439 | [PPS06201.1](https://www.ncbi.nlm.nih.gov/protein/PPS06201.1?report=genbank&log$=prottop&blast_rank=54&RID=78JKE0EJ016) | 542 | *Gossypium barbadense* | Malvaceae | 887 | 887 | 99 | 83.39 | 0.0 |
| amino-acid permease BAT1 homolog isoform X2 | [XP_010278618.1](https://www.ncbi.nlm.nih.gov/protein/XP_010278618.1?report=genbank&log$=prottop&blast_rank=55&RID=78JKE0EJ016) | 525 | *Nelumbo nucifera* | Nelumbonaceae | 886 | 886 | 97 | 86.25 | 0.0 |
| Amino-acid permease BAT1-like protein | [KAF7827746.1](https://www.ncbi.nlm.nih.gov/protein/KAF7827746.1?report=genbank&log$=prottop&blast_rank=56&RID=78JKE0EJ016) | 519 | *Senna tora* | Fabaceae | 885 | 885 | 98 | 84.60 | 0.0 |
| Amino-acid permease BAT1-like | [XP_039019572.1](https://www.ncbi.nlm.nih.gov/protein/XP_039019572.1?report=genbank&log$=prottop&blast_rank=57&RID=78JKE0EJ016) | 523 | *Hibiscus syriacus* | Malvaceae | 885 | 885 | 99 | 87.26 | 0.0 |
| Amino-acid permease BAT1 homolog isoform X3 | [XP_018843000.1](https://www.ncbi.nlm.nih.gov/protein/XP_018843000.1?report=genbank&log$=prottop&blast_rank=58&RID=78JKE0EJ016) | 515 | *Juglans regia* | Juglandaceae | 884 | 884 | 97 | 86.67 | 0.0 |
| Amino-acid permease BAT1 homolog | [XP_028758143.1](https://www.ncbi.nlm.nih.gov/protein/XP_028758143.1?report=genbank&log$=prottop&blast_rank=59&RID=78JKE0EJ016) | 518 | *Prosopis alba* | Fabaceae | 884 | 884 | 98 | 86.19 | 0.0 |
| Amino-acid permease BAT1 homolog | [XP_028794478.1](https://www.ncbi.nlm.nih.gov/protein/XP_028794478.1?report=genbank&log$=prottop&blast_rank=60&RID=78JKE0EJ016) | 518 | *Prosopis alba* | Fabaceae | 884 | 884 | 98 | 86.38 | 0.0 |
| AA_permease_2 domain-containing protein | [GAV83531.1](https://www.ncbi.nlm.nih.gov/protein/GAV83531.1?report=genbank&log$=prottop&blast_rank=61&RID=78JKE0EJ016) | 639 | *Cephalotus follicularis* | Cephalotaceae | 883 | 1012 | 97 | 87.57 | 0.0 |
| Hypothetical protein CMV_025307 | [KAF3948729.1](https://www.ncbi.nlm.nih.gov/protein/KAF3948729.1?report=genbank&log$=prottop&blast_rank=62&RID=78JKE0EJ016) | 515 | *Castanea mollissima* | Fagaceae | 882 | 882 | 97 | 86.47 | 0.0 |
| Amino-acid permease | [PSR87766.1](https://www.ncbi.nlm.nih.gov/protein/PSR87766.1?report=genbank&log$=prottop&blast_rank=63&RID=78JKE0EJ016) | 522 | *Actinidia chinensis* var*. chinensis* | Actinidiaceae | 882 | 882 | 99 | 84.62 | 0.0 |
| Amino-acid permease BAT1 homolog isoform X1 | [XP_020424682.1](https://www.ncbi.nlm.nih.gov/protein/XP_020424682.1?report=genbank&log$=prottop&blast_rank=64&RID=78JKE0EJ016) | 526 | *Prunus persica* | Rosaceae | 881 | 881 | 99 | 83.59 | 0.0 |
| Amino-acid permease BAT1 homolog isoform X2 | [XP_010663487.1](https://www.ncbi.nlm.nih.gov/protein/XP_010663487.1?report=genbank&log$=prottop&blast_rank=65&RID=78JKE0EJ016) | 522 | *Vitis vinifera* | Vitaceae | 880 | 880 | 99 | 83.59 | 0.0 |
| Hypothetical protein B456_012G123900 | [KJB77170.1](https://www.ncbi.nlm.nih.gov/protein/KJB77170.1?report=genbank&log$=prottop&blast_rank=66&RID=78JKE0EJ016) | 542 | *Gossypium raimondii* | Malvaceae | 880 | 880 | 99 | 83.76 | 0.0 |
| Hypothetical protein F511_17706 | [KZV37934.1](https://www.ncbi.nlm.nih.gov/protein/KZV37934.1?report=genbank&log$=prottop&blast_rank=67&RID=78JKE0EJ016) | 529 | *Dorcoceras hygrometricum* | Gesneriaceae | 879 | 879 | 98 | 85.05 | 0.0 |
| Amino-acid permease BAT1 homolog isoform X2 | [XP_038981580.1](https://www.ncbi.nlm.nih.gov/protein/XP_038981580.1?report=genbank&log$=prottop&blast_rank=68&RID=78JKE0EJ016) | 523 | *Phoenix dactylifera* | Arecaceae | 879 | 879 | 97 | 84.25 | 0.0 |
| Hypothetical protein JHK86_056027 | [KAG4907543.1](https://www.ncbi.nlm.nih.gov/protein/KAG4907543.1?report=genbank&log$=prottop&blast_rank=69&RID=78JKE0EJ016) | 515 | *Glycine max* | Fabaceae | 879 | 879 | 98 | 82.62 | 0.0 |
| Amino-acid permease BAT1 homolog isoform X2 | [XP_024193802.1](https://www.ncbi.nlm.nih.gov/protein/XP_024193802.1?report=genbank&log$=prottop&blast_rank=70&RID=78JKE0EJ016) | 519 | *Rosa chinensis* | Rosaceae | 878 | 878 | 97 | 86.47 | 0.0 |
| Amino-acid permease BAT1 homolog isoform X2 | [XP_034688046.1](https://www.ncbi.nlm.nih.gov/protein/XP_034688046.1?report=genbank&log$=prottop&blast_rank=71&RID=78JKE0EJ016) | 522 | *Vitis riparia* | Vitaceae | 878 | 878 | 99 | 83.40 | 0.0 |
| Amino-acid permease BAT1 homolog isoform X2 | [XP_030951228.1](https://www.ncbi.nlm.nih.gov/protein/XP_030951228.1?report=genbank&log$=prottop&blast_rank=72&RID=78JKE0EJ016) | 515 | *Quercus lobata* | Fagaceae | 877 | 877 | 97 | 86.22 | 0.0 |
| Amino-acid permease BAT1 homolog | [XP_010924660.1](https://www.ncbi.nlm.nih.gov/protein/XP_010924660.1?report=genbank&log$=prottop&blast_rank=73&RID=78JKE0EJ016) | 523 | *Elaeis guineensis* | Arecaceae | 877 | 877 | 97 | 83.14 | 0.0 |
| Amino-acid permease BAT1 homolog isoform X1 | [XP_028221837.1](https://www.ncbi.nlm.nih.gov/protein/XP_028221837.1?report=genbank&log$=prottop&blast_rank=74&RID=78JKE0EJ016) | 545 | *Glycine soja* | Fabaceae | 877 | 877 | 98 | 82.62 | 0.0 |
| Amino-acid permease BAT1 homolog isoform X1 | [XP_012075713.1](https://www.ncbi.nlm.nih.gov/protein/XP_012075713.1?report=genbank&log$=prottop&blast_rank=75&RID=78JKE0EJ016) | 529 | *Jatropha curcas* | Euphorbiaceae | 876 | 876 | 96 | 84.52 | 0.0 |
| Amino-acid permease BAT1 isoform X2 | [XP_018724528.1](https://www.ncbi.nlm.nih.gov/protein/XP_018724528.1?report=genbank&log$=prottop&blast_rank=76&RID=78JKE0EJ016) | 525 | *Eucalyptus grandis* | Myrtaceae | 876 | 876 | 99 | 84.36 | 0.0 |
| Hypothetical protein H0E87_021045 | [KAF9846235.1](https://www.ncbi.nlm.nih.gov/protein/KAF9846235.1?report=genbank&log$=prottop&blast_rank=77&RID=78JKE0EJ016) | 530 | *Populus deltoides* | Salicaceae | 876 | 876 | 96 | 83.90 | 0.0 |
| Amino-acid permease BAT1 isoform X2 | [XP_016652057.1](https://www.ncbi.nlm.nih.gov/protein/XP_016652057.1?report=genbank&log$=prottop&blast_rank=78&RID=78JKE0EJ016) | 526 | *Prunus mume* | Rosaceae | 876 | 876 | 99 | 83.37 | 0.0 |
| Amino-acid permease BAT1 homolog isoform X1 | [XP_023927079.1](https://www.ncbi.nlm.nih.gov/protein/XP_023927079.1?report=genbank&log$=prottop&blast_rank=79&RID=78JKE0EJ016) | 515 | *Quercus suber* | Fagaceae | 875 | 875 | 97 | 86.27 | 0.0 |
| Amino-acid permease BAT1 homolog | [XP_003536664.1](https://www.ncbi.nlm.nih.gov/protein/XP_003536664.1?report=genbank&log$=prottop&blast_rank=80&RID=78JKE0EJ016) | 520 | *Glycine max* | Fabaceae | 875 | 875 | 98 | 82.49 | 0.0 |
| Amino-acid permease BAT1 homolog isoform X3 | [XP_009594577.1](https://www.ncbi.nlm.nih.gov/protein/XP_009594577.1?report=genbank&log$=prottop&blast_rank=81&RID=78JKE0EJ016) | 518 | *Nicotiana tomentosiformis* | Solanaceae | 875 | 875 | 96 | 82.97 | 0.0 |
| Amino-acid permease BAT1 homolog | [XP_023927082.1](https://www.ncbi.nlm.nih.gov/protein/XP_023927082.1?report=genbank&log$=prottop&blast_rank=82&RID=78JKE0EJ016) | 515 | *Quercus suber* | Fagaceae | 875 | 875 | 97 | 85.83 | 0.0 |
| Amino-acid permease BAT1 homolog | [XP_027363632.1](https://www.ncbi.nlm.nih.gov/protein/XP_027363632.1?report=genbank&log$=prottop&blast_rank=83&RID=78JKE0EJ016) | 523 | *Abrus precatorius* | Fabaceae | 875 | 875 | 99 | 82.24 | 0.0 |
| Hypothetical protein HHK36_014932 | [KAF8399066.1](https://www.ncbi.nlm.nih.gov/protein/KAF8399066.1?report=genbank&log$=prottop&blast_rank=84&RID=78JKE0EJ016) | 524 | *Tetracentron sinense* | Trochodendraceae | 874 | 874 | 99 | 84.36 | 0.0 |
| Amino-acid permease BAT1 homolog isoform X2 | [XP_004299033.1](https://www.ncbi.nlm.nih.gov/protein/XP_004299033.1?report=genbank&log$=prottop&blast_rank=85&RID=78JKE0EJ016) | 512 | *Fragaria vesca* subsp*. vesca* | Rosaceae | 874 | 874 | 97 | 85.88 | 0.0 |
| Amino-acid permease BAT1 isoform X3 | [XP_024463350.1](https://www.ncbi.nlm.nih.gov/protein/XP_024463350.1?report=genbank&log$=prottop&blast_rank=86&RID=78JKE0EJ016) | 528 | *Populus trichocarpa* | Salicaceae | 874 | 874 | 96 | 84.03 | 0.0 |
| Amino-acid permease BAT1 homolog | [XP_010044123.2](https://www.ncbi.nlm.nih.gov/protein/XP_010044123.2?report=genbank&log$=prottop&blast_rank=87&RID=78JKE0EJ016) | 525 | *Eucalyptus grandis* | Myrtaceae | 874 | 874 | 99 | 83.78 | 0.0 |
| Amino-acid permease BAT1 homolog | [XP_010101706.1](https://www.ncbi.nlm.nih.gov/protein/XP_010101706.1?report=genbank&log$=prottop&blast_rank=88&RID=78JKE0EJ016) | 523 | *Morus notabilis* | Moraceae | 874 | 874 | 99 | 85.38 | 0.0 |
| Hypothetical protein FNV43_RR26674 | [KAF3431938.1](https://www.ncbi.nlm.nih.gov/protein/KAF3431938.1?report=genbank&log$=prottop&blast_rank=89&RID=78JKE0EJ016) | 665 | *Rhamnella rubrinervis* | Rhamnaceae | 874 | 1036 | 99 | 85.71 | 0.0 |
| Amino-acid permease BAT1 homolog | [XP_034201582.1](https://www.ncbi.nlm.nih.gov/protein/XP_034201582.1?report=genbank&log$=prottop&blast_rank=90&RID=78JKE0EJ016) | 530 | *Prunus dulcis* | Rosaceae | 874 | 874 | 99 | 83.01 | 0.0 |
| Amino-acid permease BAT1 homolog isoform X2 | [XP_031251936.1](https://www.ncbi.nlm.nih.gov/protein/XP_031251936.1?report=genbank&log$=prottop&blast_rank=91&RID=78JKE0EJ016) | 482 | *Pistacia vera* | Anacardiaceae | 874 | 874 | 92 | 91.29 | 0.0 |
| Amino-acid permease BAT1 homolog | [XP_016174788.1](https://www.ncbi.nlm.nih.gov/protein/XP_016174788.1?report=genbank&log$=prottop&blast_rank=92&RID=78JKE0EJ016) | 522 | *Arachis ipaensis* | Fabaceae | 874 | 874 | 99 | 81.73 | 0.0 |
| PREDICTED: amino-acid permease BAT1 homolog | [XP_019237447.1](https://www.ncbi.nlm.nih.gov/protein/XP_019237447.1?report=genbank&log$=prottop&blast_rank=93&RID=78JKE0EJ016) | 518 | *Nicotiana attenuata* | Solanaceae | 874 | 874 | 96 | 83.17 | 0.0 |
| Amino-acid permease BAT1-like | [TKY50896.1](https://www.ncbi.nlm.nih.gov/protein/TKY50896.1?report=genbank&log$=prottop&blast_rank=94&RID=78JKE0EJ016) | 520 | *Spatholobus suberectus* | Fabaceae | 874 | 874 | 98 | 82.88 | 0.0 |
| Hypothetical protein CMV_021683 | [KAF3952801.1](https://www.ncbi.nlm.nih.gov/protein/KAF3952801.1?report=genbank&log$=prottop&blast_rank=95&RID=78JKE0EJ016) | 579 | *Castanea mollissima* | Fagaceae | 873 | 873 | 97 | 86.08 | 0.0 |
| Amino-acid permease BAT1 | [KAG5240425.1](https://www.ncbi.nlm.nih.gov/protein/KAG5240425.1?report=genbank&log$=prottop&blast_rank=96&RID=78JKE0EJ016) | 530 | *Salix suchowensis* | Salicaceae | 872 | 872 | 96 | 83.10 | 0.0 |
| Hypothetical protein Ahy_A09g042100 | [RYR37170.1](https://www.ncbi.nlm.nih.gov/protein/RYR37170.1?report=genbank&log$=prottop&blast_rank=97&RID=78JKE0EJ016) | 1052 | *Arachis hypogaea* | Fabaceae | 872 | 1698 | 99 | 80.76 | 0.0 |
| Amino-acid permease BAT1 homolog | [XP_016482246.1](https://www.ncbi.nlm.nih.gov/protein/XP_016482246.1?report=genbank&log$=prottop&blast_rank=98&RID=78JKE0EJ016) | 518 | *Nicotiana tabacum* | Solanaceae | 872 | 872 | 96 | 82.77 | 0.0 |
| Amino-acid permease BAT1 homolog isoform X4 | [XP_031385670.1](https://www.ncbi.nlm.nih.gov/protein/XP_031385670.1?report=genbank&log$=prottop&blast_rank=99&RID=78JKE0EJ016) | 519 | *Punica granatum* | Lythraceae | 872 | 872 | 98 | 83.79 | 0.0 |
| Amino-acid permease BAT1 homolog | [XP_027934058.1](https://www.ncbi.nlm.nih.gov/protein/XP_027934058.1?report=genbank&log$=prottop&blast_rank=100&RID=78JKE0EJ016) | 520 | *Vigna unguiculata* | Fabaceae | 872 | 872 | 99 | 81.85 | 0.0 |

**^a^** The listed genes were identified based on recently available data in GenBank, National Center for Biotechnology Information website (NCBI, <https://www.ncbi.nlm.nih.gov/protein/>). Listed sequences were used to create the multiple protein sequence alignment using the Constraint-based Multiple Alignment Tool (COBALT) presented in Figure 2 and the phylogenetic tree presented in Figure 3.

## Table S6. Predicted secondary structure content of putative *CsgabP* proteins from *C. sinensis* compared with *AtgabP* from *A. thaliana*

| Feature | ***AtgabP*** (516 aa) | | ***CsgabP-*1** (521 aa) | | ***CsgabP-*2** (419 aa) | |
| --- | --- | --- | --- | --- | --- | --- |
|  | No. of AA residues | % | No. of AA residues | % | No. of AA residues | % |
| *α*-Helix | 251 | 48.64 | 255 | 48.94 | 205 | 48.93 |
| 3_10_-Helix | 0 | 0 | 0 | 0 | 0 | 0 |
| π-Helix | 0 | 0 | 0 | 0 | 0 | 0 |
| Beta bridge | 0 | 0 | 0 | 0 | 0 | 0 |
| Extended strand | 90 | 17.44 | 90 | 17.27 | 77 | 18.38 |
| Beta turn | 11 | 2.13 | 9 | 1.73 | 14 | 3.34 |
| Bend region | 0 | 0 | 0 | 0 | 0 | 0 |
| Random coil | 164 | 31.78 | 167 | 32.05 | 123 | 29.36 |
| Ambiguous states | 0 | 0 | 0 | 0 | 0 | 0 |
| Other states | 0 | 0 | 0 | 0 | 0 | 0 |

*α*-Helix: Alpha helix has 3.6 residues per helical turn in a 13-atom ring.

3_10_-Helix: 3_10_-Helix has 3 residues per helical turn in a 10-atom ring.

π-Helix: Pi-Helix is a wider helix with 4.6 residues per turn.

## Table S7. Helices of 3D structure of *CsgabP*-1 as predicted using PDBsum Pictorial database

| **No.** | **Start** | **End** | **Type** | **No. resid** | **Length** | **Unit rise** | **Residues per turn** | **Pitch** | **Deviation from ideal** | **Sequence** |
| --- | --- | --- | --- | --- | --- | --- | --- | --- | --- | --- |
| 1. | Met 40 | Ser 47 | H | 8 | 12.12 | 1.50 | 3.61 | 5.42 | 13.4 | MLSNFAFS |
| 2. | Phe 48 | Ile 50 | G | 3 | - | - | - | - | - | FSI |
| 3. | Ser 52 | Thr 55 | H | 4 | 4.44 | 1.03 | 4.21 | 4.36 | 43.7 | SVLT |
| 4. | Thr 63 | Asn 66 | H | 4 | 6.23 | 1.41 | 3.71 | 5.25 | 39.6 | TGLN |
| 5. | Pro 70 | Phe 83 | H | 14 | 20.11 | 1.43 | 3.61 | 5.16 | 7.0 | PISLVYGWLIAGAF |
| 6. | Phe 86 | Cys 95 | H | 10 | 15.48 | 1.49 | 3.66 | 5.46 | 6.2 | FVGSSMAEIC |
| 7. | Tyr 105 | Ala 109 | H | 5 | 7.81 | 1.49 | 3.67 | 5.47 | 23.0 | YYWSA |
| 8. | Trp 116 | Gly 131 | H | 16 | 24.26 | 1.48 | 3.70 | 5.47 | 20.3 | WAPFASWMTGWFNIVG |
| 9. | Asp 140 | Thr 155 | H | 16 | 24.35 | 1.51 | 3.65 | 5.50 | 8.1 | DFSLAQMIQVIILLST |
| 10. | Gly 161 | Tyr 163 | G | 3 | - | - | - | - | - | GGY |
| 11. | Lys 167 | Ser 186 | H | 20 | 30.43 | 1.50 | 3.67 | 5.51 | 8.3 | KYVVIAFHGGILLLHAIINS |
| 12. | Leu 192 | Trp 202 | H | 11 | 16.08 | 1.42 | 3.72 | 5.30 | 8.9 | LSFFGQLAAAW |
| 13. | Leu 204 | Glu 220 | H | 17 | 26.25 | 1.51 | 3.68 | 5.56 | 8.0 | LVGVMVLMILIPSVSTE |
| 14. | Asp 237 | Ile 239 | G | 3 | - | - | - | - | - | DGI |
| 15. | Phe 246 | Gly 249 | G | 4 | 8.30 | 2.12 | 3.30 | 6.98 | 36.2 | FVLG |
| 16. | Leu 250 | Tyr 255 | H | 6 | 9.72 | 1.51 | 3.53 | 5.34 | 5.7 | LLMSQY |
| 17. | Asp 261 | His 265 | G | 5 | 9.03 | 1.80 | 4.61 | 8.27 | 29.8 | DASAH |
| 18. | Met 266 | Glu 269 | H | 4 | 6.78 | 1.60 | 3.73 | 5.98 | 38.9 | MTEE |
| 19. | Asn 276 | Phe 301 | H | 26 | 38.33 | 1.45 | 3.77 | 5.45 | 12.1 | NGPKGIISAIGISIIFGWGYILGITF |
| 20. | Ala 320 | Phe 329 | H | 10 | 13.99 | 1.41 | 3.60 | 5.09 | 7.6 | AIAEIFYLAF |
| 21. | Gly 339 | Met 364 | H | 26 | 40.11 | 1.52 | 3.66 | 5.57 | 20.4 | GIVCLGVVAVAIFFCGMSSVTSNSRM |
| 22. | Tyr 366 | Arg 370 | H | 5 | 8.63 | 1.63 | 3.42 | 5.58 | 19.1 | YAFSR |
| 23. | Ser 377 | Phe 379 | G | 3 | - | - | - | - | - | SSF |
| 24. | Ile 390 | Ala 404 | H | 15 | 22.49 | 1.46 | 3.69 | 5.39 | 5.6 | INAVWLSAFISFCMA |
| 25. | Ala 411 | Ala 440 | H | 30 | 44.48 | 1.46 | 4.28 | 6.26 | 42.8 | AVAFQAMVSIATIGLYIAYALPIFFRVTLA |
| 26. | Ile 456 | Val 471 | H | 16 | 24.78 | 1.51 | 3.63 | 5.48 | 8.4 | IVVGWIAVLWVATISV |
| 27. | Cys 494 | Phe 506 | H | 13 | 20.00 | 1.50 | 3.89 | 5.84 | 27.6 | CGLLILTVSAWIF |

The data displayed in the table for each helix includes the helix number (assigned sequentially starting with 1 at the N-terminus of the protein), the residue numbers corresponding to the start and end of the helices, the helix type; **H**: alpha helix (has 3.6 residues per helical turn in a 13-atom ring) or **G**: 3_10_ helix (has 3 residues per helical turn in a 10-atom ring). This is followed by the number of residues in the helix and information about the geometry of the helix as follows: length and unit rise (both in Angstroms), the number of residues per turn (ideally 3.6) for alpha helices), the helix pitch in Angstroms and a measure of the deviation of the helix geometry from an ideal helix (in degrees). This latter value should be 0 for a perfect helix. The geometrical parameters are not calculated for helices with fewer than four residues. The final column in the table gives the helix's amino acid sequence.

## Table S8. Helix-helix interactions of 3D structure of *CsgabP*-1 as predicted using PDBsum Pictorial database

| **No.** | **Helices** | | **Helix**  **types** | | **Distance (Å)** | **Angle (°)** | **Interaction** | | **No. interacting residues** | | |
| --- | --- | --- | --- | --- | --- | --- | --- | --- | --- | --- | --- |
|  |  |  |  |  |  |  | **type** | | **Total** | **Helix 1** | **Helix 2** |
| 1. | A1 | A17 | H | G | 9.5 | 135.4 | c | n | 2 | 2 | 1 |
| 2. | A1 | A18 | H | H | 10.4 | 169.9 | N | C | 2 | 2 | 1 |
| 3. | A1 | A19 | H | H | 8.3 | -43.2 | I | I | 7 | 2 | 6 |
| 4. | A3 | A13 | H | H | 6.9 | 88.0 | C | I | 6 | 3 | 3 |
| 5. | A3 | A19 | H | H | 7.5 | -32.9 | N | I | 4 | 2 | 3 |
| 6. | A4 | A5 | H | H | 8.3 | 101.7 | C | I | 2 | 1 | 2 |
| 7. | A4 | A16 | H | H | 8.6 | -126.9 | c | n | 3 | 2 | 2 |
| 8. | A4 | A19 | H | H | 9.6 | -85.1 | I | I | 7 | 3 | 5 |
| 9. | A4 | A20 | H | H | 7.6 | 82.9 | n | n | 1 | 1 | 1 |
| 10. | A4 | A25 | H | H | 11.3 | -81.8 | N | I | 1 | 1 | 1 |
| 11. | A5 | A6 | H | H | 4.2 | -24.5 | c | n | 2 | 2 | 1 |
| 12. | A5 | A16 | H | H | 9.9 | -45.1 | I | I | 3 | 3 | 1 |
| 13. | A5 | A19 | H | H | 9.4 | 163.4 | I | I | 10 | 6 | 6 |
| 14. | A5 | A26 | H | H | 9.2 | -136.5 | C | I | 5 | 3 | 3 |
| 15. | A6 | A7 | H | H | 10.6 | 81.0 | I | I | 3 | 3 | 1 |
| 16. | A6 | A17 | H | G | 7.1 | -22.9 | C | C | 5 | 3 | 3 |
| 17. | A6 | A18 | H | H | 8.6 | -63.3 | I | I | 2 | 2 | 1 |
| 18. | A6 | A19 | H | H | 11.5 | 149.0 | I | I | 2 | 2 | 1 |
| 19. | A6 | A25 | H | H | 10.2 | 43.7 | I | I | 11 | 5 | 5 |
| 20. | A6 | A26 | H | H | 8.3 | -127.5 | N | I | 5 | 3 | 4 |
| 21. | A7 | A8 | H | H | 6.4 | 146.3 | C | N | 7 | 3 | 5 |
| 22. | A7 | A17 | H | G | 10.2 | 72.0 | N | I | 2 | 1 | 2 |
| 23. | A7 | A22 | H | H | 9.8 | -62.4 | n | n | 1 | 1 | 1 |
| 24. | A7 | A25 | H | H | 7.5 | 46.7 | C | I | 1 | 1 | 1 |
| 25. | A8 | A16 | H | H | 13.1 | -113.8 | I | C | 1 | 1 | 1 |
| 26. | A8 | A21 | H | H | 9.9 | -159.1 | C | I | 3 | 2 | 2 |
| 27. | A8 | A22 | H | H | 10.1 | -139.1 | I | N | 3 | 3 | 1 |
| 28. | A8 | A24 | H | H | 9.3 | -56.5 | I | I | 4 | 2 | 3 |
| 29. | A8 | A25 | H | H | 9.4 | -158.1 | I | I | 16 | 7 | 9 |
| 30. | A8 | A27 | H | H | 10.1 | 145.7 | I | I | 12 | 5 | 7 |
| 31. | A9 | A11 | H | H | 9.8 | 112.8 | I | I | 6 | 4 | 4 |
| 32. | A9 | A20 | H | H | 8.6 | -37.0 | N | N | 4 | 3 | 3 |
| 33. | A9 | A21 | H | H | 8.7 | -158.2 | I | I | 18 | 8 | 8 |
| 34. | A9 | A25 | H | H | 10.7 | -153.5 | N | I | 1 | 1 | 1 |
| 35. | A11 | A12 | H | H | 11.4 | 133.4 | c | n | 1 | 1 | 1 |
| 36. | A11 | A21 | H | H | 10.4 | -58.0 | I | I | 9 | 5 | 5 |
| 37. | A11 | A24 | H | H | 9.8 | 172.4 | N | C | 10 | 7 | 5 |
| 38. | A12 | A13 | H | H | 1.4 | -10.4 | c | n | 7 | 4 | 3 |
| 39. | A12 | A21 | H | H | 8.7 | -146.4 | I | I | 7 | 4 | 6 |
| 40. | A13 | A19 | H | H | 8.8 | 55.5 | I | I | 5 | 5 | 4 |
| 41. | A13 | A20 | H | H | 10.1 | 45.3 | I | I | 8 | 5 | 5 |
| 42. | A13 | A21 | H | H | 8.8 | -137.5 | N | I | 6 | 3 | 5 |
| 43. | A15 | A16 | G | H | 0.9 | 27.2 | c | n | 7 | 3 | 4 |
| 44. | A15 | A25 | G | H | 9.3 | 77.9 | C | I | 1 | 1 | 1 |
| 45. | A16 | A25 | H | H | 7.8 | 52.3 | I | I | 11 | 4 | 7 |
| 46. | A16 | A26 | H | H | 8.4 | -134.4 | n | c | 5 | 3 | 3 |
| 47. | A17 | A18 | G | H | 2.1 | -41.9 | I | N | 7 | 3 | 4 |
| 48. | A17 | A25 | G | H | 13.3 | 26.5 | N | I | 1 | 1 | 1 |
| 49. | A19 | A20 | H | H | 8.2 | 59.2 | C | I | 5 | 3 | 3 |
| 50. | A20 | A21 | H | H | 10.6 | 157.7 | I | N | 4 | 3 | 3 |
| 51. | A21 | A22 | H | H | 3.7 | -20.0 | c | n | 4 | 3 | 2 |
| 52. | A21 | A24 | H | H | 7.8 | 129.0 | C | N | 5 | 3 | 3 |
| 53. | A24 | A25 | H | H | 11.2 | 131.9 | C | N | 1 | 1 | 1 |
| 54. | A25 | A26 | H | H | 8.1 | -155.1 | I | I | 12 | 7 | 8 |
| 55. | A25 | A27 | H | H | 10.6 | -39.9 | I | N | 4 | 3 | 2 |

Pairs are included in the table if one or both of the helices is in the current polypeptide chain. The helix numbers for each helix, the distance of closest approach (in Angstroms) and the interaction angle (omega) are given. The interaction type describes where in each of the helices the distance of closest approach occurs (**C**: beyond the C terminus of the helix, **N**: beyond the N terminus of the helix, **I**: internal to the helix). The number of interacting pairs of residues and the number of residues in each of the two helices involved in the interaction are also given.

## Table S9. Beta turns of 3D structure of *CsgabP*-1 as predicted using PDBsum Pictorial database

| **No.** | **Turn** | **Sequence** | **Turn**  **type** | **Residue i+1** | | | **Residue i+2** | | | **i to i+3** | **H-bond** |
| --- | --- | --- | --- | --- | --- | --- | --- | --- | --- | --- | --- |
|  |  |  |  | **Phi** | **Psi** | **Chi1** | **Phi** | **Psi** | **Chi1** | **CA-dist** |  |
| 1. | Gly 56 – Thr 59 | GITT | I | -51.6 | -47.1 | -73.4 | -94.6 | 6.0 | 53.9 | 5.8 | No |
| 2. | Ile 57 – Leu 60 | ITTL | IV | -94.6 | 6.0 | 53.9 | -126.4 | -74.0 | -63.9 | 5.9 | Yes |
| 3. | Thr 58 – Tyr 61 | TTLY | IV | -126.4 | -74.0 | -63.9 | -61.2 | -38.7 | -139.8 | 6.2 | Yes |
| 4. | Thr 59 – Asn 62 | TLYN | I | -61.2 | -38.7 | -139.8 | -118.4 | 19.8 | -55.1 | 5.1 | No |
| 5. | Ser 97 – Thr 100 | SYPT | IV | -59.1 | 109.8 | -160.0 | 133.9 | -3.2 | -36.7 | 6.0 | No |
| 6. | Pro 99 – Gly 102 | PTSG | IV | -22.8 | -51.8 | -52.8 | -69.0 | 74.8 | -58.0 | 6.5 | Yes |
| 7. | Gly 102 – Tyr 105 | GGLY | I' | 41.4 | 56.3 | - | 66.6 | -27.2 | -175.2 | 5.7 | No |
| 8. | Ala 109 – Ala 112 | AKLA | IV | -97.4 | -4.5 | -57.9 | -125.5 | -24.7 | -56.3 | 5.6 | Yes |
| 9. | Lys 110 – Gly 113 | KLAG | IV | -125.5 | -24.7 | -56.3 | -110.5 | -8.7 | - | 5.6 | Yes |
| 10. | Val 135 - Ser 138 | VTTS | IV | 59.7 | 178.5 | -61.2 | -62.9 | -37.2 | 34.3 | 6.9 | Yes |
| 11. | Thr 136 – Val 139 | TTSV | I | -62.9 | -37.2 | 34.3 | -52.6 | 0.9 | 52.5 | 6.0 | No |
| 12. | Thr 155 – Lys 158 | TGGK | I' | 73.5 | 40.0 | - | 83.8 | 5.4 | - | 5.6 | Yes |
| 13. | Tyr 163 – Ser 166 | YEAS | IV | -102.7 | -60.5 | 57.3 | -67.2 | -34.9 | - | 6.2 | Yes |
| 14. | Ile 189 – Leu 192 | ISIL | IV | -109.2 | -20.1 | -57.5 | -105.9 | -22.5 | -61.3 | 5.9 | Yes |
| 15. | Ala 222 – Lys 225 | ASAK | IV | -173.9 | -50.4 | 51.7 | -57.3 | -39.3 | - | 6.4 | Yes |
| 16. | Ser 223 – Phe 226 | SAKF | I | -57.3 | -39.3 | - | -61.8 | -38.4 | -74.5 | 5.5 | Yes |
| 17. | Ala 224 – Val 227 | AKFV | I | -61.8 | -38.4 | -74.5 | -68.6 | -38.2 | -47.7 | 4.9 | Yes |
| 18. | Asn 232 – Asn 235 | NSDN | IV | -76.7 | -52.5 | 45.7 | -162.7 | 51.8 | -156.6 | 6.5 | Yes |
| 19. | Val 243 – Phe 246 | VYIF | I | -54.3 | -41.2 | -57.5 | -91.3 | -15.2 | 45.3 | 6.7 | No |
| 20. | Leu 257 – Tyr 260 | LTGY | IV | -84.9 | 161.4 | 73.6 | 82.0 | 5.9 | - | 6.8 | No |
| 21. | Thr 270 – Ala 273 | TKNA | IV | -93.9 | 0.3 | 68.7 | 169.9 | 107.6 | 168.1 | 6.0 | Yes |
| 22. | Ala 273 – Asn 276 | ADRN | I | -40.3 | -56.2 | -52.1 | -108.6 | -26.3 | 100.3 | 5.3 | Yes |
| 23. | Thr 304 – Pro 307 | TSIP | I | -63.1 | -53.8 | 179.7 | -112.3 | 21.4 | 60.9 | 5.9 | Yes |
| 24. | Pro 307 – Leu 310 | PNLL | I | -86.1 | -6.4 | 47.8 | -100.4 | -1.2 | -56.3 | 6.0 | No |
| 25. | Asn 308 – Ser 311 | NLLS | IV | -100.4 | -1.2 | -56.3 | -82.0 | -19.0 | -178.0 | 6.1 | Yes |
| 26. | Leu 309 – Glu 312 | LLSE | IV | -82.0 | -19.0 | -178.0 | -177.4 | 30.8 | 46.0 | 6.4 | Yes |
| 27. | Glu 312 – Asp 315 | EDND | VIII | -66.3 | -44.9 | 171.9 | -125.5 | 132.4 | -160.1 | 6.0 | Yes |
| 28. | Phe 329 – Arg 332 | FKNR | I | -73.1 | -19.1 | -172.1 | -113.2 | -3.5 | -65.0 | 5.3 | Yes |
| 29. | Met 374 – Ser 377 | MPFS | I | -70.4 | -5.1 | -27.3 | -124.5 | 23.6 | -169.3 | 5.5 | No |
| 30. | Asn 384 – Asp 387 | NSQD | II | -65.9 | 124.7 | -165.5 | 88.8 | 38.9 | -160.9 | 5.5 | No |
| 31. | Ala 404 – Tyr 407 | ALTY | I | -69.1 | -19.2 | -58.6 | -103.3 | -2.9 | 59.1 | 5.6 | Yes |
| 32. | Ala 440 – Ser 443 | ARKS | IV | -125.4 | 12.7 | -58.4 | -140.2 | -22.5 | -58.2 | 6.3 | Yes |
| 33. | Gly 447 – Asn 450 | GPFN | VIII | -63.2 | -23.0 | -28.9 | -135.8 | 130.4 | 174.7 | 6.2 | Yes |
| 34. | Leu 451 – Tyr 454 | LGRY | IV | -93.7 | -69.8 | - | -94.5 | 12.9 | -64.2 | 5.6 | Yes |
| 35. | Tyr 479 – Thr 482 | YPIT | IV | -74.6 | 61.3 | -21.9 | 61.0 | 85.4 | -37.5 | 5.1 | Yes |
| 36. | Pro 480 – Ser 483 | PITS | IV | 61.0 | 85.4 | -37.5 | 79.7 | -54.0 | -63.4 | 6.5 | Yes |
| 37. | Thr 485 – Tyr 488 | TLNY | I | -41.1 | -39.9 | -50.9 | -99.0 | -1.5 | -58.6 | 4.9 | No |
| 38. | Asn 487 – Pro 490 | NYTP | IV | -66.5 | -50.8 | -178.8 | -63.9 | -40.8 | -46.5 | 5.7 | Yes |
| 39. | Tyr 488 – Val 491 | YTPV | I | -63.9 | -40.8 | -46.5 | -65.2 | -31.1 | -27.3 | 5.2 | Yes |

A beta turn is defined for 4 consecutive residues (denoted by i, i+1, i+2 and i+3) if the distance between the C-alpha atom of residue i and the C-alpha atom of residue i+3 is less than 7Å and if the central two residues are not helical (either using the Kabsch and Sander criteria or using author defined criteria) ^16^. The following data about each beta turn are shown: The residue numbers of residues i and i+3 in the turn, the one-letter amino acid code of residues i, i+1, i+2 and i+3 in the turn, and the turn type. For each of the central two residues (i+1 and i+2) phi, psi and chi1 are recorded. The final columns show the distance between the C-alpha atoms of residues i and i+3 and whether or not a hydrogen bond exists between these two residues. The phi and psi angles are allowed to vary by +/- 30 degrees from these ideal values with the added flexibility of one angle being allowed to deviate by as much as 40 degrees. Types VIa1, VIa2 and VIb turns are subject to the additional condition that residue i must be a *cis*-proline. Turns which do not fit any of the above criteria are classified as type IV.

## Table S10. Gamma turns of 3D structure of *CsgabP*-1 as predicted using PDBsum Pictorial database

| **No.** | **Start** | **End** | **Sequence** | **Turn type** | **Residue i+1** | | | **i to i+2**  **CA-dist** |
| --- | --- | --- | --- | --- | --- | --- | --- | --- |
|  |  |  |  |  | **Phi** | **Psi** | **Chi1** |  |
| 1. | Cys 95 | Ser 97 | CSS | Inverse | -78.5 | 72.0 | 55.2 | 5.5 |
| 2. | Thr 100 | Gly 102 | TSG | Inverse | -69.0 | 74.8 | -58.0 | 5.6 |
| 3. | Gly 103 | Tyr 105 | GLY | Classic | 66.6 | -27.2 | -175.2 | 5.5 |
| 4. | Ala 165 | Lys 167 | ASK | Inverse | -87.4 | 99.1 | -168.8 | 6.0 |
| 5. | Pro 188 | Ser 190 | PIS | Inverse | -72.5 | 65.6 | -47.3 | 5.5 |
| 6. | Ser 335 | Val 337 | SGV | Classic | 74.1 | -41.3 | - | 5.5 |
| 7. | Ile 388 | Ile 390 | IPI | Inverse | -72.6 | 89.5 | 22.6 | 5.6 |
| 8. | Tyr 479 | Ile 481 | YPI | Inverse | -74.6 | 61.3 | -21.9 | 4.8 |
| 9. | Ile 481 | Ser 483 | ITS | Classic | 79.7 | -54.0 | -63.4 | 5.9 |

A Gamma turn is defined for 3 residues i, i+1, i+2 if a hydrogen bond exists between residues i and i+2 and the phi and psi angles of residue i+1 fall within 40 degrees of one of the following 2 classes ^17,18^: Classic type (phi_(i+1)_= 75.0 and psi_(i+1)_= -64.0) and Inverse type (phi_(i+1)_= -79.0 and psi_(i+1)_= 69.0). The start and end residues of the gamma turn (residues i and i+2), the amino acid sequence of the residues in the turn and the turn type. Phi, psi and chi1 dihedral angles are given for the central residue (i+1). The final column gives the distance between the C-alpha atoms of residues i and i+2.

## Table S11. Helices of 3D structure of *CsgabP*-2 as predicted using PDBsum Pictorial database

| **No.** | **Start** | **End** | **Type** | **No.**  **resid** | **Length** | **Unit**  **rise** | **Residues**  **per turn** | **Pitch** | **Deviation**  **from ideal** | **Sequence** |
| --- | --- | --- | --- | --- | --- | --- | --- | --- | --- | --- |
| 1. | Met 40 | Ser 47 | H | 8 | 12.16 | 1.51 | 3.61 | 5.44 | 13.4 | MLSNFAFS |
| 2. | Phe 48 | Ile 50 | G | 3 | - | - | - | - | - | FSI |
| 3. | Thr 63 | Asn 66 | H | 4 | 6.79 | 1.53 | 3.61 | 5.53 | 39.3 | TGLN |
| 4. | Pro 70 | Ala 82 | H | 13 | 19.59 | 1.48 | 3.59 | 5.32 | 6.3 | PISLVYGWLIAGA |
| 5. | Phe 86 | Cys 95 | H | 10 | 15.87 | 1.52 | 3.65 | 5.56 | 9.0 | FVGSSMAEIC |
| 6. | Tyr 105 | Leu 111 | H | 7 | 10.83 | 1.54 | 3.72 | 5.73 | 8.0 | YYWSAKL |
| 7. | Trp 116 | Trp 133 | H | 18 | 26.58 | 1.48 | 3.62 | 5.36 | 21.8 | WAPFASWMTGWFNIVGQW |
| 8. | Val 139 | Thr 155 | H | 17 | 25.82 | 1.51 | 3.62 | 5.46 | 6.7 | VDFSLAQMIQVIILLST |
| 9. | Gly 162 | Ala 165 | H | 4 | 4.92 | 1.00 | 4.26 | 4.24 | 41.7 | GYEA |
| 10. | Lys 167 | Asn 185 | H | 19 | 28.33 | 1.48 | 3.65 | 5.41 | 9.1 | KYVVIAFHGGILLLHAIIN |
| 11. | Leu 192 | Trp 202 | H | 11 | 16.05 | 1.42 | 3.72 | 5.29 | 9.2 | LSFFGQLAAAW |
| 12. | Leu 204 | Glu 220 | H | 17 | 26.35 | 1.52 | 3.68 | 5.60 | 9.0 | LVGVMVLMILIPSVSTE |
| 13. | Asp 237 | Ile 239 | G | 3 | - | - | - | - | - | DGI |
| 14. | Phe 246 | Gly 249 | G | 4 | 8.16 | 2.09 | 3.15 | 6.59 | 38.9 | FVLG |
| 15. | Leu 250 | Tyr 255 | H | 6 | 9.73 | 1.50 | 3.51 | 5.28 | 5.1 | LLMSQY |
| 16. | Asp 261 | Thr 267 | G | 7 | 12.27 | 1.75 | 3.36 | 5.86 | 24.8 | DASAHMT |
| 17. | Asn 276 | Thr 300 | H | 25 | 36.07 | 1.43 | 3.70 | 5.29 | 10.5 | NGPKGIISAIGISIIFGWGYILGIT |
| 18. | Ile 321 | Phe 329 | H | 9 | 14.12 | 1.54 | 3.65 | 5.61 | 8.5 | IAEIFYLAF |
| 19. | Gly 339 | Met 364 | H | 26 | 39.95 | 1.52 | 3.66 | 5.55 | 19.5 | GIVCLGVVAVAIFFCGMSSVTSNSRM |
| 20. | Ala 367 | Asp 371 | H | 5 | 8.12 | 1.56 | 3.45 | 5.37 | 19.8 | AFSRD |
| 21. | Ser 377 | Phe 379 | G | 3 | - | - | - | - | - | SSF |
| 22. | Ile 390 | Cys 402 | H | 13 | 19.71 | 1.48 | 3.67 | 5.42 | 4.9 | INAVWLSAFISFC |

The data displayed in the table for each helix includes the helix number (assigned sequentially starting with 1 at the N-terminus of the protein), the residue numbers corresponding to the start and end of the helices, the helix type; **H**: alpha helix (has 3.6 residues per helical turn in a 13-atom ring) or **G**: 3_10_ helix (has 3 residues per helical turn in a 10-atom ring). This is followed by the number of residues in the helix and information about the geometry of the helix as follows: length and unit rise (both in Angstroms), the number of residues per turn (ideally 3.6) for alpha helices), the helix pitch in Angstroms and a measure of the deviation of the helix geometry from an ideal helix (in degrees). This latter value should be 0 for a perfect helix. The geometrical parameters are not calculated for helices with fewer than four residues. The final column in the table gives the helix's amino acid sequence.

## Table S12. Helix-helix interactions of 3D structure of *CsgabP*-2 as predicted using PDBsum Pictorial database

| **No.** | **Helices** | | **Helix**  **types** | | **Distance (Å)** | **Angle (°)** | **Interaction**  **type** | | **No. interacting residues** | | |
| --- | --- | --- | --- | --- | --- | --- | --- | --- | --- | --- | --- |
|  |  |  |  |  |  |  |  |  | **Total** | **Helix 1** | **Helix 2** |
| 1. | A1 | A16 | H | G | 10.0 | 149.6 | C | I | 4 | 3 | 2 |
| 2. | A1 | A17 | H | H | 8.5 | -43.3 | I | I | 7 | 2 | 6 |
| 3. | A3 | A4 | H | H | 7.9 | 106.8 | C | N | 2 | 1 | 2 |
| 4. | A3 | A15 | H | H | 9.1 | -129.0 | C | N | 2 | 1 | 2 |
| 5. | A3 | A17 | H | H | 9.7 | -80.9 | I | I | 4 | 3 | 3 |
| 6. | A3 | A18 | H | H | 7.7 | 76.2 | N | N | 1 | 1 | 1 |
| 7. | A4 | A15 | H | H | 10.2 | -45.8 | I | I | 3 | 3 | 1 |
| 8. | A4 | A17 | H | H | 9.5 | 164.6 | I | I | 9 | 6 | 6 |
| 9. | A5 | A6 | H | H | 9.9 | 83.0 | I | I | 4 | 3 | 2 |
| 10. | A5 | A16 | H | G | 7.7 | -38.6 | I | I | 7 | 3 | 4 |
| 11. | A5 | A17 | H | H | 11.5 | 147.3 | I | I | 2 | 2 | 1 |
| 12. | A6 | A7 | H | H | 6.9 | 143.0 | I | N | 7 | 3 | 5 |
| 13. | A6 | A16 | H | G | 10.2 | 87.1 | N | I | 2 | 1 | 2 |
| 14. | A6 | A20 | H | H | 10.6 | -54.3 | N | N | 2 | 1 | 2 |
| 15. | A7 | A8 | H | H | 2.8 | -7.4 | C | N | 4 | 3 | 2 |
| 16. | A7 | A10 | H | H | 11.3 | 119.5 | C | I | 1 | 1 | 1 |
| 17. | A7 | A15 | H | H | 12.7 | -114.8 | I | C | 3 | 3 | 1 |
| 18. | A7 | A19 | H | H | 10.3 | -160.7 | C | I | 6 | 4 | 4 |
| 19. | A7 | A20 | H | H | 10.3 | -140.3 | I | N | 4 | 4 | 1 |
| 20. | A7 | A22 | H | H | 9.8 | -54.3 | I | I | 4 | 2 | 3 |
| 21. | A8 | A9 | H | H | 7.5 | 161.7 | C | N | 6 | 5 | 2 |
| 22. | A8 | A10 | H | H | 9.6 | 112.1 | I | I | 8 | 5 | 4 |
| 23. | A8 | A18 | H | H | 8.5 | -32.6 | N | N | 5 | 3 | 4 |
| 24. | A8 | A19 | H | H | 8.6 | -158.7 | I | I | 18 | 9 | 8 |
| 25. | A9 | A10 | H | H | 5.3 | -75.5 | C | I | 7 | 4 | 4 |
| 26. | A10 | A11 | H | H | 12.0 | 134.3 | C | N | 1 | 1 | 1 |
| 27. | A10 | A19 | H | H | 10.5 | -57.9 | I | I | 9 | 5 | 5 |
| 28. | A10 | A22 | H | H | 10.3 | 172.5 | N | C | 6 | 4 | 4 |
| 29. | A11 | A12 | H | H | 1.4 | -9.5 | C | N | 7 | 4 | 3 |
| 30. | A11 | A19 | H | H | 8.7 | -146.6 | I | I | 7 | 4 | 6 |
| 31. | A12 | A17 | H | H | 8.6 | 56.3 | I | I | 5 | 5 | 3 |
| 32. | A12 | A18 | H | H | 10.4 | 44.2 | I | I | 8 | 5 | 5 |
| 33. | A12 | A19 | H | H | 8.7 | -138.2 | N | I | 6 | 3 | 5 |
| 34. | A14 | A15 | G | H | 0.6 | 24.6 | C | N | 7 | 3 | 4 |
| 35. | A17 | A18 | H | H | 8.8 | 55.0 | C | I | 4 | 3 | 2 |
| 36. | A18 | A19 | H | H | 10.0 | 161.5 | I | N | 5 | 3 | 3 |
| 37. | A19 | A20 | H | H | 5.3 | -20.7 | C | N | 2 | 1 | 2 |
| 38. | A19 | A22 | H | H | 7.9 | 129.4 | C | N | 5 | 3 | 3 |

Pairs are included in the table if one or both of the helices is in the current polypeptide chain. The helix numbers for each helix, the distance of closest approach (in Angstroms) and the interaction angle (omega) are given. The interaction type describes where in each of the helices the distance of closest approach occurs (**C**: beyond the C terminus of the helix, **N**: beyond the N terminus of the helix, **I**: internal to the helix). The number of interacting pairs of residues and the number of residues in each of the two helices involved in the interaction are also given.

## Table S13. Beta turns of 3D structure of *CsgabP*-2 as predicted using PDBsum Pictorial database

| **No.** | **Turn** | **Sequence** | **Turn**  **type** | **Residue i+1** | | | **Residue i+2** | | | **i to i+3**  **CA-dist** | **H-bond** |
| --- | --- | --- | --- | --- | --- | --- | --- | --- | --- | --- | --- |
|  |  |  |  | **Phi** | **Psi** | **Chi1** | **Phi** | **Psi** | **Chi1** |  |  |
| 1. | Ile 51 – Leu 54 | ISVL | IV | -119.5 | -23.4 | -69.9 | -84.9 | -7.9 | 96.4 | 6.1 | Yes |
| 2. | Ser 52 – Thr 55 | SVLT | IV | -84.9 | -7.9 | 96.4 | -102.9 | -91.7 | -37.9 | 5.5 | Yes |
| 3. | Val 53 – Gly 56 | VLTG | IV | -102.9 | -91.7 | -37.9 | -90.0 | -13.2 | 60.3 | 6.9 | Yes |
| 4. | Gly 56 – Thr 59 | GITT | I | -55.6 | -39.6 | -69.3 | -96.2 | -3.8 | 46.4 | 5.8 | No |
| 5. | Ile 57 – Leu 60 | ITTL | IV | -96.2 | -3.8 | 46.4 | -128.0 | -71.5 | 45.1 | 6.0 | Yes |
| 6. | Thr 58 – Tyr 61 | TTLY | IV | -128.0 | -71.5 | 45.1 | -58.9 | -42.7 | -138.9 | 6.1 | Yes |
| 7. | Thr 59 – Asn 62 | TLYN | I | -58.9 | -42.7 | -138.9 | -118.0 | 23.0 | -57.7 | 5.2 | No |
| 8. | Ser 97 – Thr 100 | SYPT | IV | -62.6 | 117.1 | -54.7 | 127.2 | 1.4 | -37.1 | 6.1 | No |
| 9. | Pro 99 – Gly 102 | PTSG | IV | -23.1 | -51.3 | -44.5 | -71.4 | 74.1 | -56.3 | 6.7 | Yes |
| 10. | Gly 102 – Tyr 105 | GGLY | I' | 42.4 | 55.8 | - | 66.6 | -25.6 | -169.7 | 5.7 | No |
| 11. | Ala 134 – Thr 137 | AVTT | IV | -76.4 | 121.0 | -65.5 | -133.0 | 69.5 | 60.4 | 6.4 | No |
| 12. | Val 135 – Ser 138 | VTTS | IV | -133.0 | 69.5 | 60.4 | -127.8 | 8.7 | 47.3 | 6.4 | Yes |
| 13. | Thr 155 – Lys 158 | TGGK | I' | 73.3 | 38.6 | - | 84.1 | 3.6 | - | 5.6 | Yes |
| 14. | Ile 189 – Leu 192 | ISIL | IV | -107.8 | -21.2 | -57.6 | -103.8 | -23.7 | -60.7 | 5.9 | Yes |
| 15. | Ala 222 – Lys 225 | ASAK | IV | -173.8 | -50.7 | 52.4 | -57.1 | -38.8 | - | 6.4 | Yes |
| 16. | Ser 223 – Phe 226 | SAKF | I | -57.1 | -38.8 | - | -61.3 | -38.2 | -74.3 | 5.5 | Yes |
| 17. | Ala 224 – Val 227 | AKFV | I | -61.3 | -38.2 | -74.3 | -70.3 | -36.6 | -47.7 | 5.0 | Yes |
| 18. | Asn 232 – Asn 235 | NSDN | IV | -76.5 | -52.6 | 45.9 | -163.6 | 55.3 | -157.5 | 6.5 | Yes |
| 19. | Val 243 – Phe 246 | VYIF | I | -57.4 | -41.1 | -163.6 | -94.7 | -3.6 | 61.5 | 6.6 | No |
| 20. | Leu 257 – Tyr 260 | LTGY | II | -77.6 | 149.5 | 66.3 | 92.9 | 2.1 | - | 6.5 | No |
| 21. | Thr 267 – Thr 270 | TEET | I | -62.3 | -39.8 | -92.5 | -73.4 | -17.2 | -51.4 | 5.2 | Yes |
| 22. | Thr 270 – Ala 273 | TKNA | IV | -95.2 | 2.8 | 68.8 | 169.9 | 110.8 | 167.5 | 6.1 | Yes |
| 23. | Ala 273 – Asn 276 | ADRN | I | -42.6 | -55.5 | -51.1 | -111.3 | -24.8 | 97.9 | 5.4 | Yes |
| 24. | Thr 300 – Val 303 | TFAV | I | -65.3 | -38.9 | -170.5 | -117.6 | 18.8 | - | 5.0 | Yes |
| 25. | Asn 308 – Ser 311 | NLLS | I | -75.3 | -11.3 | -69.8 | -77.0 | -8.4 | -174.3 | 5.7 | No |
| 26. | Asp 313 – Ala 316 | DNDA | I | -65.8 | -35.7 | -66.5 | -76.4 | -22.2 | -69.2 | 5.3 | Yes |
| 27. | Asn 314 – Gly 317 | NDAG | I | -76.4 | -22.2 | -69.2 | -98.5 | 13.5 | - | 4.5 | No |
| 28. | Phe 329 – Arg 332 | FKNR | I | -73.6 | -19.0 | -172.5 | -113.3 | -2.7 | -66.2 | 5.3 | Yes |
| 29. | Met 374 – Ser 377 | MPFS | I | -61.4 | -30.7 | -28.7 | -104.8 | 16.8 | -169.6 | 5.6 | No |
| 30. | Asn 384 – Asp 387 | NSQD | II | -65.8 | 125.3 | -165.4 | 87.0 | 39.8 | -160.9 | 5.5 | No |

A beta turn is defined for 4 consecutive residues (denoted by i, i+1, i+2 and i+3) if the distance between the C-alpha atom of residue i and the C-alpha atom of residue i+3 is less than 7Å and if the central two residues are not helical (either using the Kabsch and Sander criteria or using author defined criteria) ^16^. The following data about each beta turn are shown: The residue numbers of residues i and i+3 in the turn, the one-letter amino acid code of residues i, i+1, i+2 and i+3 in the turn, and the turn type. For each of the central two residues (i+1 and i+2) phi, psi and chi1 are recorded. The final columns show the distance between the C-alpha atoms of residues i and i+3 and whether or not a hydrogen bond exists between these two residues. The phi and psi angles are allowed to vary by +/- 30 degrees from these ideal values with the added flexibility of one angle being allowed to deviate by as much as 40 degrees. Types VIa1, VIa2 and VIb turns are subject to the additional condition that residue i must be a *cis*-proline. Turns which do not fit any of the above criteria are classified as type IV.

## Table S14. Gamma turns of 3D structure of *CsgabP*-2 as predicted using PDBsum Pictorial database

| **No.** | **Start** | **End** | **Sequence** | **Turn**  **type** | **Residue i+1** | | | **i to i+2**  **CA-dist** |
| --- | --- | --- | --- | --- | --- | --- | --- | --- |
|  |  |  |  |  | **Phi** | **Psi** | **Chi1** |  |
| 1. | Cys 95 | Ser 97 | CSS | Inverse | -81.5 | 61.0 | 59.7 | 5.5 |
| 2. | Thr 100 | Gly 102 | TSG | Inverse | -71.4 | 74.1 | -56.3 | 5.6 |
| 3. | Ala 165 | Lys 167 | ASK | Inverse | -89.1 | 99.2 | -171.4 | 6.1 |
| 4. | Pro 188 | Ser 190 | PIS | Inverse | -72.6 | 65.3 | -47.8 | 5.5 |
| 5. | Ser 335 | Val 337 | SGV | Classic | 74.8 | -41.2 | - | 5.6 |
| 6. | Ile 388 | Ile 390 | IPI | Inverse | -72.1 | 89.2 | 22.9 | 5.6 |

A Gamma turn is defined for 3 residues i, i+1, i+2 if a hydrogen bond exists between residues i and i+2 and the phi and psi angles of residue i+1 fall within 40 degrees of one of the following 2 classes ^17,18^: Classic type (phi_(i+1)_= 75.0 and psi_(i+1)_= -64.0) and Inverse type (phi_(i+1)_= -79.0 and psi_(i+1)_= 69.0). The start and end residues of the gamma turn (residues i and i+2), the amino acid sequence of the residues in the turn and the turn type. Phi, psi and chi1 dihedral angles are given for the central residue (i+1). The final column gives the distance between the C-alpha atoms of residues i and i+2.

## Table S15. Predicted functional partners of *CsgabP* as predicted through protein-protein interaction networks functional enrichment analysis using STRING Consortium (Version 11.0)

| **GenBank accession No.** | **Description** | **Abbreviation** | **aa** | **Score** |
| --- | --- | --- | --- | --- |
| XP_006464439.1 | Cationic amino acid transporter 9, chloroplastic | *CsCAT9* | 578 | 0.693 |
| XP_006490894.1 | Arginase 1, mitochondrial-like isoform X1 | *CsARG1* | 339 | 0.613 |
| XP_006478102.1 | Glutamate decarboxylase 5-like | *CsGAD5* | 498 | 0.554 |
| XP_006469385.1 | Glyoxylate/succinic semialdehyde reductase 1 isoform X1 | *CsGLYR* | 291 | 0.532 |
| XP_006492278.1 | Glutamate decarboxylase-like | *CsGAD* | 494 | 0.496 |
| XP_006493749.1 | Succinate-semialdehyde dehydrogenase, mitochondrial isoform X2 | *CsSSADH* | 530 | 0.489 |
| XP_006481368.1 | Gamma aminobutyrate transaminase 3, chloroplastic-like | *CsgabT* | 514 | 0.463 |
| XP_006471571.1 | Urea-proton symporter DUR3-like isoform X1 | *CsDUR3* | 707 | 0.451 |
| XP_006476644.1 | δ-1-pyrroline-5-carboxylate dehydrogenase 12A1, mitochondrial-like isoform X1 | *CsALDH12A1* | 553 | 0.446 |
| XP_006492842.1 | Thioredoxin-like 3-1, chloroplastic-like isoform X1 | *CsTXNL1* | 189 | 0.420 |

## Table S16. Predicted functional partners of *CsgabP*-1 as predicted through protein-protein interaction networks functional enrichment analysis using STRING Consortium (Version 12.0)

| **Accession No.** | **Description** | **Abbreviation** | **Score** |
| --- | --- | --- | --- |
| A0A067F3T9 | Uncharacterized protein; Belongs to the cytochrome P450 family | - | 0.373 |
| A0A067CZM5 | SOD_Fe_N domain-containing protein | *SOD-Fe* | 0.361 |
| A0A067DEQ7 | SOD_Fe_C domain-containing protein | *SOD-Fe* | 0.361 |
| A0A067DJB0 | SOD_Fe_C domain-containing protein | *SOD-Fe* | 0.361 |
| A0A067DTM3 | Uncharacterized protein | *-* | 0.361 |
| A0A067DVA7 | SOD_Fe_N domain-containing protein | *SOD-Fe* | 0.361 |
| A0A067F2M6 | Superoxide dismutase | *SOD* | 0.361 |
| A0A067F3F0 | Superoxide dismutase | *SOD* | 0.361 |
| A0A067G2E9 | Superoxide dismutase | *SOD* | 0.361 |
| A0A067G6X1 | 3-Dehydroquinate synthase domain-containing protein | *DHQ_synthase* | 0.361 |

## Table S17. Docking score and RMSD-refine of GABA, succinic semialdehyde, and succinic acid against putative *CsgabP* proteins from *C. sinensis*

| **Tested compounds** | ***CsgabP*-1** | | ***CsgabP*-2** | |
| --- | --- | --- | --- | --- |
|  | **Docking (Affinity) score**  (Kcal·mol^−1^) | **RMSD-Refine**  (Å) | **Docking (Affinity) score**  (Kcal·mol^−1^) | **RMSD-Refine**  (Å) |
| GABA | -4.26 | 2.72 | -4.10 | 2.70 |
| Succinic semialdehyde | -4.08 | 1.97 | -4.07 | 1.82 |
| Succinic acid | -4.35 | 2.03 | -4.23 | 2.42 |

RMSD: root-mean-square deviations of atomic locations.

# References

1. Flores-Gonzalez, M. *et al.* Citrusgreening.org: An open access and integrated systems biology portal for the Huanglongbing (HLB) disease complex. *bioRxiv* 868364 at https://doi.org/10.1101/868364 (2019).

2. Michaeli, S. *et al.* A mitochondrial GABA permease connects the GABA shunt and the TCA cycle, and is essential for normal carbon metabolism. *Plant J.* **67**, 485–498 (2011).

3. Altschul, S. F. *et al.* Protein database searches using compositionally adjusted substitution matrices. *FEBS J.* **272**, 5101–5109 (2005).

4. Altschul, S. F. *et al.* Gapped BLAST and PSI-BLAST: a new generation of protein database search programs. *Nucleic Acids Res.* **25**, 3389–402 (1997).

5. Papadopoulos, J. S. & Agarwala, R. COBALT: Constraint-based alignment tool for multiple protein sequences. *Bioinformatics* **23**, 1073–1079 (2007).

6. Larkin, M. A. *et al.* Clustal W and Clustal X version 2.0. *Bioinformatics* **23**, 2947–2948 (2007).

7. Madeira, F. *et al.* The EMBL-EBI Job Dispatcher sequence analysis tools framework in 2024. *Nucleic Acids Res.* **52**, W521–W525 (2024).

8. Jones, D. T., Taylor, W. R. & Thornton, J. M. The rapid generation of mutation data matrices from protein sequences. *Bioinformatics* **8**, 275–282 (1992).

9. Kumar, S., Stecher, G., Li, M., Knyaz, C. & Tamura, K. MEGA X: Molecular evolutionary genetics analysis across computing platforms. *Mol. Biol. Evol.* **35**, 1547–1549 (2018).

10. Gasteiger, E. *et al.* Protein Identification and Analysis Tools on the ExPASy Server. in *The Proteomics Protocols Handbook* (ed. John M. Walker) 571–607 (Humana Totowa, NJ, 2005).

11. Bailey, T. L. & Elkan, C. Fitting a mixture model by expectation maximization to discover motifs in biopolymers. (1994).

12. Blum, M. *et al.* The InterPro protein families and domains database: 20 years on. *Nucleic Acids Res.* **49**, D344–D354 (2021).

13. Combet, C., Blanchet, C., Geourjon, C. & Deléage, G. NPS@: network protein sequence analysis. *Trends Biochem. Sci.* **25**, 147–150 (2000).

14. Geourjon, C. & Deléage, G. SOPMA: significant improvements in protein secondary structure prediction by consensus prediction from multiple alignments. *Comput. Appl. Biosci.* **11**, 681–684 (1995).

15. Laskowski, R. A., Chistyakov, V. V. & Thornton, J. M. PDBsum more: new summaries and analyses of the known 3D structures of proteins and nucleic acids. *Nucleic Acids Res.* **33**, D266–D268 (2005).

16. Lewis, P. N., Momany, F. A. & Scheraga, H. A. Chain reversals in proteins. *Biochim. Biophys. Acta* **303**, 211–229 (1973).

17. Rose, G. D., Glerasch, L. M. & Smith, J. A. Turns in peptides and proteins. *Adv. Protein Chem.* **37**, 1–109 (1985).

18. Milner-White, E. J., Ross, B. M., Ismail, R., Belhadj-Mostefa, K. & Poet, R. One type of gamma-turn, rather than the other gives rise to chain-reversal in proteins. *J. Mol. Biol.* **204**, 777–782 (1988).

19. Biasini, M. *et al.* SWISS-MODEL: modelling protein tertiary and quaternary structure using evolutionary information. *Nucleic Acids Res.* **42**, W252–W258 (2014).

20. Pettersen, E. F. *et al.* UCSF Chimera?A visualization system for exploratory research and analysis. *J. Comput. Chem.* **25**, 1605–1612 (2004).

21. Jumper, J. *et al.* Highly accurate protein structure prediction with AlphaFold. *Nat. 2021 5967873* **596**, 583–589 (2021).

22. Bryant, P., Pozzati, G. & Elofsson, A. Improved prediction of protein-protein interactions using AlphaFold2. *Nat. Commun. 2022 131* **13**, 1–11 (2022).

23. Mirdita, M. *et al.* ColabFold: making protein folding accessible to all. *Nat. Methods 2022 196* **19**, 679–682 (2022).

24. Kelley, L. A., Mezulis, S., Yates, C. M., Wass, M. N. & Sternberg, M. J. E. The Phyre2 web portal for protein modeling, prediction and analysis. *Nat. Protoc.* **10**, 845–858 (2015).

25. Sonnhammer, E. L. L., Von Heijne, G. & Krogh, A. A hidden Markov model for predicting transmembrane helices in protein sequences. (1998).

26. Szklarczyk, D. *et al.* STRING v11: protein–protein association networks with increased coverage, supporting functional discovery in genome-wide experimental datasets. *Nucleic Acids Res.* **47**, D607–D613 (2019).

27. Madhavi Sastry, G., Adzhigirey, M., Day, T., Annabhimoju, R. & Sherman, W. Protein and ligand preparation: parameters, protocols, and influence on virtual screening enrichments. *J. Comput. Aided. Mol. Des.* **27**, 221–234 (2013).

28. Grosdidier, A., Zoete, V. & Michielin, O. Fast docking using the CHARMM force field with EADock DSS. *J. Comput. Chem.* **32**, 2149–2159 (2011).

29. Trott, O. & Olson, A. J. AutoDock Vina: Improving the speed and accuracy of docking with a new scoring function, efficient optimization, and multithreading. *J. Comput. Chem.* **31**, 455–461 (2010).

30. Tatineni, S. *et al.* In planta distribution of ‘Candidatus Liberibacter asiaticus’ as revealed by polymerase chain reaction (PCR) and real-time PCR. *Phytopathology* **98**, 592–9 (2008).

31. Nehela, Y. & Killiny, N. Melatonin is involved in citrus response to the pathogen huanglongbing via modulation of phytohormonal biosynthesis. *Plant Physiol.* **184**, 2216–2239 (2020).

32. Nehela, Y. & Killiny, N. Gamma-Aminobutyric Acid Accumulation Contributes to Citrus sinensis Response against ‘Candidatus Liberibacter Asiaticus’ via Modulation of Multiple Metabolic Pathways and Redox Status. *Plants 2023, Vol. 12, Page 3753* **12**, 3753 (2023).

33. Nehela, Y. & Killiny, N. Gamma-Aminobutyric Acid Supplementation Boosts the Phytohormonal Profile in ‘Candidatus Liberibacter asiaticus’-Infected Citrus. *Plants* **12**, 3647 (2023).

34. Hijaz, F., Nehela, Y. & Killiny, N. Application of gamma-aminobutyric acid increased the level of phytohormones in Citrus sinensis. *Planta* **248**, 909–918 (2018).

35. Hijaz, F. & Killiny, N. The use of deuterium-labeled gamma-aminobutyric (D6-GABA) to study uptake, translocation, and metabolism of exogenous GABA in plants. *Plant Methods* **16**, (2020).

36. Mafra, V. *et al.* Reference genes for accurate transcript normalization in citrus genotypes under different experimental conditions. *PLoS One* **7**, e31263 (2012).

37. Wei, X. *et al.* Novel expression patterns of carotenoid pathway-related genes in citrus leaves and maturing fruits. *Tree Genet. Genomes* **10**, 439–448 (2014).
